# Integrating palliative care and heart failure: a protocol for a realist synthesis (PalliatHeartSynthesis)

**DOI:** 10.1136/bmjopen-2021-058848

**Published:** 2022-01-07

**Authors:** Tracey McConnell, John Burden, Claire Duddy, Loreena Hill, Clare Howie, Bob Jones, Bob Ruane, Geoff Wong, Joanne Reid

**Affiliations:** 1 School of Nursing and Midwifery, Queen's University Belfast, Belfast, UK; 2 Marie Curie Hospice, Belfast, UK; 3 Patient and Public Involvement Group, British Heart Foundation, London, UK; 4 Nuffield Department of Primary Care Health Sciences, University of Oxford, Oxford, UK

**Keywords:** heart failure, palliative care, organisation of health services

## Abstract

**Introduction:**

Heart failure affects over 26 million people worldwide with prevalence expected to grow due to an ageing global population. Palliative care can address the holistic needs of patients with heart failure, and integrated palliative care in heart failure management has been indicated to improve outcomes for patients. Despite known benefits for integrated palliative care in heart failure management, implementation is poor across the majority of global health services. Recent systematic reviews have identified the benefits of integrating palliative care into heart failure management and highlighted barriers to implementation. However, there was heterogeneity in terms of countries, healthcare settings, delivery by differing staff across multidisciplinary teams, modes of delivery and different intervention components.

**Methods and analysis:**

The aim of this study is to identify how integrated palliative care and heart failure interventions produce desired outcomes, in which contexts, and for which patients. We will undertake a realist synthesis to identify this, using Pawson’s five iterative steps. We will recruit an international stakeholder group comprised of healthcare providers and patients with heart failure to advise and provide feedback throughout the review. Our initial realist programme theory sets out the necessary steps needed to accomplish the final intended outcome(s) from the implementation of integrated palliative care and heart failure. This initial programme theory will be shaped through an iterative process of testing and refinement.

**Ethics and dissemination:**

Ethical approval is not required for this study. With our stakeholder group, we will coproduce a user guide that outlines practical advice to optimise, tailor and implement interventions designed to integrate palliative care and heart failure, taking into consideration local context, alongside user-friendly summaries of the synthesis findings using short animations to convey complex findings. We will draw on the expertise within the stakeholder group to identify key stakeholders for disseminating to relevant audiences, ensuring outputs are tailored for their respective needs.

**PROSPERO registration number:**

CRD42021240185.

**Strengths and limitations of this study**

- This is the first realist synthesis examining the integration of palliative care into routine heart failure management.
- This synthesis will be supported by a large multidisciplinary, international stakeholder group of policy, practice and patient experts to maximise the potential for widespread implementation, reduce healthcare costs and improve outcomes for patients and informal carers.
- Potential limitations include lack of relevance and rigour in the available literature.

## Introduction

### Background

Cardiovascular disease is the most common cause of death worldwide.^1^ Heart failure characterises the final phenotype of many cardiovascular diseases, estimated to currently affect at least 26 million people worldwide,^2^ with this number expected to grow due to ageing populations.^3^ Patients with advanced heart failure or those classified as experiencing New York Heart Association class III or class IV symptoms, account for over one million hospitalisations per year in the USA and Europe,^4^ with an overall estimated economic cost of US$108 billion per annum.^5^ Integrating palliative care with routine management of heart failure has been shown to significantly reduce healthcare costs overall compared with usual care^6^ and significantly reduces the number of hospital visits and duration of inpatient stays.^7 8^ The National Audit Office review of end-of-life care recommended palliative care for patients with heart failure, due to a possible cost savings by reducing utilisation of acute services.^9^

There is evidence of improved patient and family outcomes when palliative care is integrated in advanced heart failure management. A review of carers’ needs identified that integrated palliative care in heart failure management led to an improvement in satisfaction with care from both the patient and their informal carer.^10^ Informal carers are typically defined as those who provide unpaid care to individuals with whom they have a relationship, that is, family members or spouses.^11^ Informal carers are crucial to facilitating independent living and supporting quality of life (QoL) for patients with heart failure and palliative care can address caregivers’ needs and help them care for their loved one.^12^ Integrated palliative care in heart failure management can benefit QoL, symptom burden and levels of depression in patients with the condition.^13^

Integrated palliative care aims to achieve continuity of care by integrating administrative, organisational and clinical services that make up the patients care network.^14^ Examples of integrated palliative care and heart failure interventions include collaborations and shared goal setting between palliative care and clinical cardiology teams to ameliorate symptoms with palliative care goals, alongside heart failure management.^15^ In addition, social work led palliative care services alongside heart failure management^16^ aimed to improve the physical, psychological, social, spiritual and end-of-life outcomes of patients. In 2020, the European Association for Palliative Care Task Force^17^ concluded that the inclusion of palliative care within the regular clinical framework for people with heart failure provides improvement in QoL as well as comfort and dignity. This was echoed in a position paper by the Heart Failure Association (HFA) of the European Society of Cardiology (ESC)^18^ who stated that many patients with heart failure would benefit from earlier integration of a palliative approach into the care provided by the multidisciplinary team involved.

However, although two decades have passed since the first publication on the benefits of palliative care for patients with advanced heart failure,^19^ the HFA Atlas identified only 10 out of 42 European countries with designated palliative care units for patients with advanced heart failure.^20^ The poor integration of palliative care into advanced heart failure management can be explained by a number of factors, including uncertainty around the heart failure disease trajectory and complexities of communicating this uncertainty to patient and family members. Heart failure is an unpredictable illness, with periods of stability of symptoms, interjected with numerous exacerbations, of which many can lead to a temporary improvement in health status; others can lead to a progressive decline towards death. Many patients with advanced heart failure overestimate survival by up to 40%, further adding to cardiology providers’ reluctance to initiate difficult conversations around prognosis. This difficulty is further compounded by lack of patient and practitioner knowledge around what palliative care is; misunderstanding that palliative care equals end-of-life care only. The fragmentation of inpatient and outpatient services also creates a barrier to the holistic need’s assessment required for an integrated palliative care approach.^21^

### Overview of existing evidence

Until recently, the lack of evidence from clinical trials demonstrating benefits of palliative care for people with advanced heart failure posed an additional barrier. However, there has been an exponential increase in published literature since the turn of the century, increasing from 10 publications on average in 2000 to over 100 publications per year in 2017 (23). McIlvennan and Allen^21^ published a review summarising the evolving role of palliative care for patients with heart failure, along with the barriers and opportunities for its integration into routine practice. Findings from the review highlighted the need for evidence on how best to integrate palliative care and heart failure given the cultural and environmental differences in how palliative care services are delivered. Three systematic reviews of palliative care interventions for patients with heart failure by Diop *et al*,^22^ Datla *et al*^13^ and Sahlollbey *et al*^23^ all highlighted the benefits of palliative care in heart failure management for patient-centred outcomes and reducing hospital utilisation. A recent scoping review examining elements of integrated palliative care in heart failure management^24^ identified the need for a multidisciplinary approach to integration and for cardiology staff to champion the benefits of palliative care. This review also highlighted the need for research with robust theoretical underpinnings given the complex behaviour changes required for sustaining integrated care in practice.

A recent editorial^25^ exploring the phenomenon of inconsistent implementation of integrated palliative care and heart failure interventions proposed a realist approach could provide a sound theoretical understanding of the barriers and facilitators to routine implementation. The research to date has focused on ‘trying to evidence effectiveness through a linear cause and effect approach, which fails to ignore the messy, non-linear world of real-life practice’ (25 p.1). Overall, Datla *et al*^13^ concluded that there is no clear consensus around: (1) the core components of integrated palliative care and heart failure interventions, (2) the ideal configuration for the multidisciplinary team and (3) the most effective service provision model to ensure that generalist and/or specialist palliative care is tailored to patient needs.

The issue of heterogeneity was further highlighted in a narrative literature review aimed at identifying the key characteristics of integrated palliative care and heart failure interventions.^26^ Of the nine studies included, all integrated palliative care and heart failure interventions were implemented in different countries with different models of health service provision for citizens (USA, Sweden, Hong Kong), different settings (inpatient, outpatient and home based), delivered by a heterogeneous mix of multidisciplinary teams (cardiologists, heart failure nurses, general practitioners, community nurses, occupational therapists), using different modes of delivery (face to face, telemedicine) and involving different intervention components (symptom management, advance care planning). Therefore, we still do not know which intervention produces the best outcomes for patients and their informal carers (what works: specialist vs primary care, etc?), when best to initiate palliative care (for whom; at what stage in the disease trajectory?), or the optimal delivery method (in what circumstances; required infrastructure, staff competencies, etc?).

### Rationale explaining why this research is important now

Globally the population is living longer than ever before, and there are now 703 million individuals aged 65 or over in the world.^27^ Projections estimate that those over 60 years will increase from 12% of the world’s population to 22% by 2050, with one in six individuals in the world being aged 60 or over by 2030.^28^ Although we can celebrate this achievement in life expectancy, it comes with significant challenges for healthcare now and in the future. Older people have complex health needs, with on average 4.5 comorbidities. Heart failure often dominates their physical and psychological needs,^21^ along with being the costliest aspect of their care due to high rates of hospitalisation and futile pharmaceutical and surgical interventions as their heart failure progresses.^29^ Older people with advanced heart failure have undeniably had their needs overlooked, with calls for more attention to, and research for this vulnerable group to ensure they receive appropriate, effective treatment and care.^21 30^ The 2021 ESC guidelines for the diagnosis and treatment of acute and chronic heart failure highlighted the need for studies to determine specific options for palliative care in the treatment of heart failure.^31^

Although we have some promising examples of integrated palliative care and heart failure interventions,^15 16^ there is heterogeneity in terms of countries, healthcare settings, delivery by mix of multidisciplinary teams, modes of delivery and different intervention components.^32^ Hence, this review is vital for identifying which model works best, for whom or in what circumstances.

## Aims and objectives

### Aim

To understand how integrated palliative care and heart failure interventions may work in different healthcare settings for example, inpatient/outpatient and for which groups of people, so we can recommend strategies to maximise the potential for widespread implementation, reduce healthcare costs and improve QoL for patients and informal carers

### Objectives

1. To conduct a realist synthesis to build an understanding of which integrated palliative care and heart failure interventions work best, in which contexts and for which patients and informal carers.
2. To coproduce recommendations with an expert stakeholder group, to maximise potential for widespread implementation through a user guide for healthcare providers and user friendly summaries for patients and the public.

### Review questions

1. What are the mechanisms by which integrated palliative care and heart failure interventions work in order to produce their intended outcomes?
2. What are the contexts, which determine whether integrated palliative care and heart failure interventions produce their intended or unintended outcomes?
3. In what settings are integrated palliative care and heart failure interventions likely to be effective?

## Methods and analysis

### Objective 1: conduct a realist synthesis

**Figure 1:**
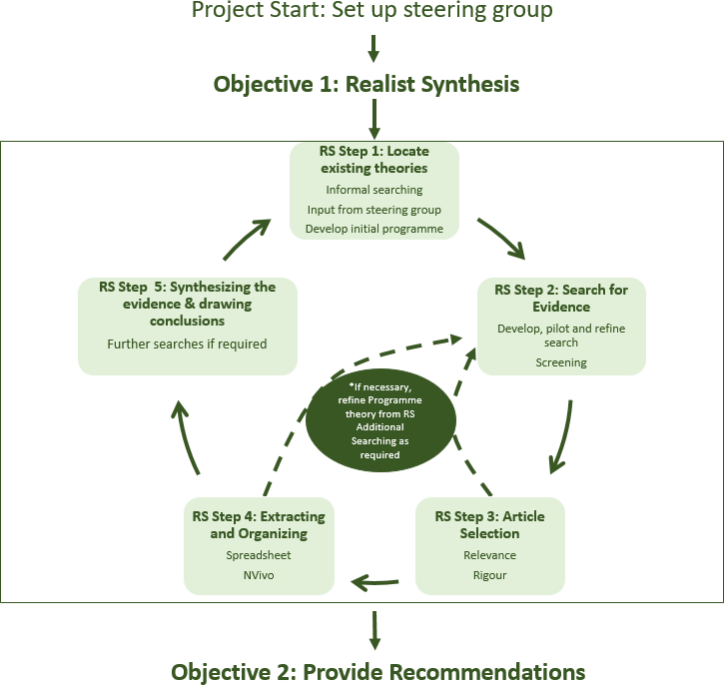
Project flow diagram using Pawson’s (2005) five iterative steps. RS, Realist synthesis.

This realist synthesis will follow Pawson’s (2005) five iterative steps for realist synthesis (see figure 1). Realist syntheses are particularly suited to research on integrated palliative care and heart failure as they focus on making sense of the contextual factors that determine the outcomes of an intervention. Like other interventions that seek to propagate behavioural change, implementation of integrated palliative care and heart failure is highly context dependent, that is, implementation of the same intervention will vary in its success depending, for example, on who delivers it and how it is delivered, the characteristics of the health care professionals (HCPs) involved, the circumstances surrounding it, and the tools and techniques used. Research designs that seek to ‘strip away’ these contexts limit our understanding of ‘how, when and for whom’ the intervention will be effective. A realist synthesis takes context as central to any explanation by exploring how an intervention manipulates context to trigger mechanisms that cause behavioural change.

**Figure 2:**
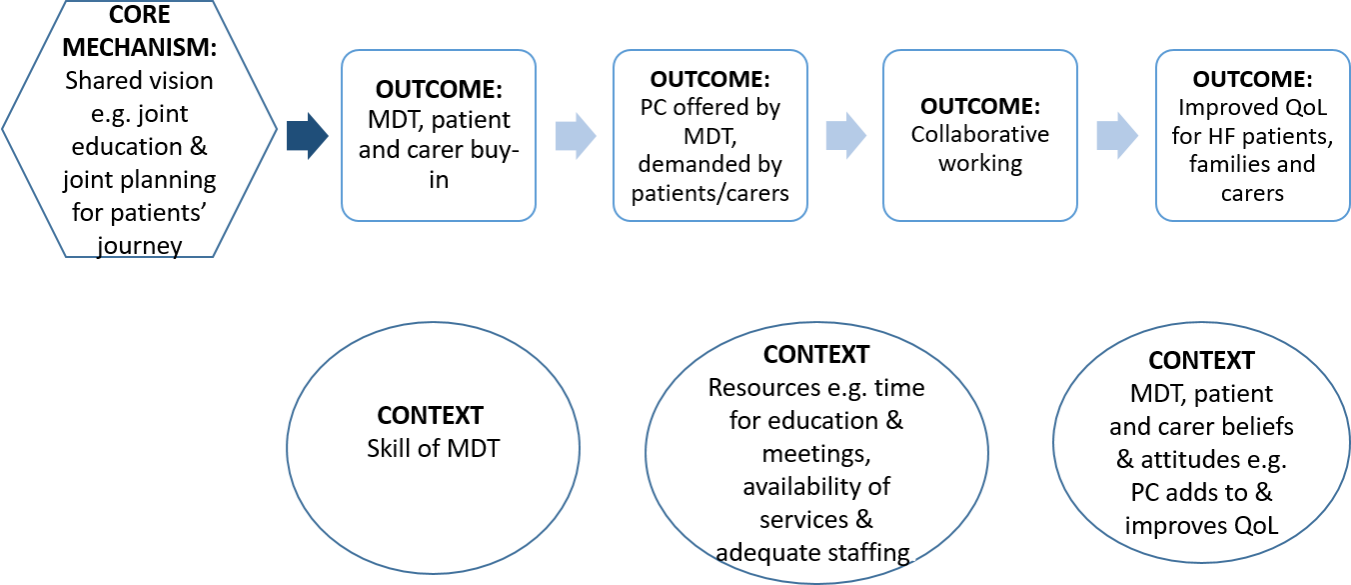
Preliminary initial programme theory. HF, heart failure; QoL, quality of life; PC, palliative care; MDT, multidisciplinary team.

The explanation building will start with the development of an initial programme theory of ‘how integrated palliative care and heart failure interventions produce desired outcomes (or not)’. To achieve this, our preliminary initial realist programme theory sets out the necessary steps needed to accomplish the final intended outcome(s) from implementation of integrated palliative care and heart failure (see figure 2). This initial programme theory will be shaped through an iterative process of testing—that is, parts of it are confirmed, refuted or refined against a range of relevant data from existing literature (see steps 1–5 below). This protocol has been registered with PROSPERO. The realist synthesis will adhere to the relevant quality and publication standards.^32^ Figure 1 provides the project overview and key steps are outlined below in more detail.

### Patient and public involvement

We will consult with UK-wide and international stakeholders with both content and lived experience expertise, including National Health Service (NHS) managers and HCPs involved in the delivery of palliative care and heart failure management, relevant charities and patient and public involvement (PPI). Our initial programme theory will be refined throughout the course of the project based on regular consultation, via five virtual meetings and email correspondence with our stakeholder group. Our stakeholders, including PPI will also provide their perspectives on findings and recommendations, provide support with dissemination and contribute to all project materials to ensure they meet the needs of patients and the wider public.

### Step 1: locate existing theories

The purpose of this step is to locate existing theories that explain why, how, in what contexts, for whom and to what extent does integrated palliative care and heart failure work to bring about improved outcomes for advanced heart failure patients and their informal carers. While we have already established there is limited theory underlying integrated palliative care and heart failure interventions, the realist synthesis approach allows for the literature net to be cast wider to include literature from other fields and other professions where potentially shared mechanisms may be in operation.

To identify these theories, we shall iteratively: (1) consult with key content experts in our stakeholder group and (2) informally search the literature to identify existing theories. The informal searches conducted in step 1 differ from the more formal searching that will be carried in step 2 as their purpose is to quickly identify the kinds of theory that may be relevant; thus exploratory and informal search methods including, citation tracking and snow-balling based on known existing studies will be used. Once the theories have been identified, we shall build an initial programme theory (drawing on the preliminary initial programme theory—refer figure 2) to test in the synthesis. Programme theory development will necessitate iterative discussions within the research team to bring together the different theories into an initial programme theory.

### Step 2: search for evidence

#### Formal search

The goal of step 2 is to find a body of relevant literature in order to further develop and refine the initial programme theory developed in step 1. The searches will be designed, piloted and carried out by an experienced information specialist (CD) with experience of carrying out iterative searches for realist synthesis. We will search academic databases including CINAHL, PsycINFO, PubMed, MEDLINE, AMED and EMBASE. A search for grey literature will also be carried out in sources including Google, OpenGrey, National Institute for Health and Care Excellence and Health Management Information Consortium. Citation searching will also be undertaken including ‘cited by’ searches and searches of citations in the reference lists of relevant documents. We shall also ask the research team and steering group to identify any literature they may think is relevant. The databases will be searched using free-text keywords and controlled vocabulary where appropriate, using terms such as advanced heart failure AND palliative care. The strategy will be informed by our informal scoping searches, search strategies employed in existing reviews and knowledge of the project team. Any literature that is likely to provide conceptually rich data, including grey literature, will be considered for inclusion in the synthesis.

#### Screening

When screening the identified literature, the inclusion (table 1) and exclusion criteria will be deliberately broad as we seek to identify quantitative, qualitative, mixed-methods and non-empirical documents.

**Table 1:** Inclusion criteria for identified literature

Categories	Inclusion criteria
Document types	All documents focused on palliative care for advanced heart failure patients.
Study design	All study designs. Non-empirical data (eg, from opinion/commentary pieces) which help direct/shape theory development.
Types of settings	All documents about inpatient or outpatient or home-based care settings.
Types of participant	All adult patients (18 years and over). The majority of research has involved patients with advanced heart failure (defined as NYHA class III and IV).
Types of intervention	Any combination of palliative care strategies for the management of patients with advanced heart failure.
Outcome measures	All integrated palliative care and heart failure related outcome measures.

NYHA, New York Heart Association.

Table 1 shows the categories and details for inclusion criteria for identified literature.

The screening process will be piloted with small samples of documents until agreement on the application of the inclusion criteria is reached within the research team. CH will undertake screening of all titles and abstracts, and TM will independently review a 10% random sample of retrieved citations for quality control. If there are disagreements these will be resolved through discussion between members of the research team. In the event of disagreements remaining, these will be discussed and resolved by majority vote among the project research team. Documents included after title and abstract screening will then be screened again against inclusion criteria based on the full text. The same quality assurance process as described above will be used.

#### Additional searching

A vital part of conducting a realist synthesis involves the option to search for additional data to explain particular parts of the programme theory. Therefore, more searches will be conducted if we need more data to develop and test specific areas of the programme theory. Based on our understanding of integrated palliative care and heart failure to date, these could include areas like clinician/patient/informal carers attitude towards and understanding of palliative care, non-specialist palliative care education and training, having difficult conversations. These additional topics will increase the quantity of relevant data available for us to test the programme theory. The searches will be developed, piloted and refined by the Information Specialist (CD), along with the research team. These searches will differ from the ‘formal searches’ outlined above through being more exploratory and purposive, and from a range of different disciplines. Each additional search instigated, along with the inclusion and exclusion criteria, will be discussed by the research team to ensure alignment with objectives.

### Step 3: article selection

We are aware that there will be evidence that is of variable quality. However, it is because of this potential challenge that we have chosen to use a realist review approach. For example, full text documents will be selected based on relevance (whether data can contribute to theory building and/or testing) and rigour (whether methods used to generate the relevant data are credible and trustworthy). Assessments will also be made of the plausibility and coherence at the level of the programme theory.^33^ To ensure consistency, a random sample of 10% of included papers will be assessed for relevance and discussed between TM and CH. CH will make decisions on the relevance and rigour of the remaining 90%. In the event of any uncertainty, the review team will discuss the relevance or rigour of the articles and any disagreements will be resolved through research team member discussions. A majority vote among the research team will be used to resolve any remaining disagreements.

### Step 4: extracting and organising data

CH will undertake organisation and data extraction. Full texts of the included articles will be imported into NVivo (a qualitative data management software tool). Relevant sections of texts relating to one or more parts of the programme theory will be coded in NVivo first by conceptual ‘themes’ and then as the synthesis progresses these will be developed into context–mechanism–outcome (CMO) configurations (CMOCs) (see step 5 below), the appropriateness of which is outlined in the literature.^32^ Data on the characteristics of the documents will be extracted separately into an Excel spreadsheet. As a quality assurance process, a random 10% of the coding will be independently checked by JR, with any disagreement resolved by discussion within the team.

### Step 5: synthesis the evidence and drawing conclusions

A realist logic of analysis will be used to analyse the data from included documents. Interpretive cross-case comparison will be used to understand and explain how and why actual outcomes have happened, for example, by comparing integrated palliative care and heart failure interventions that have been successful against those which have not, in order to understand how context has influenced reported findings.^32^ CH will be supported by GW, CD and TM to undertake this step using the following reasoning processes typically used in synthesising evidence in realist synthesis:

1. Juxtaposition of sources of evidence, for example, where evidence about behaviour change in one source allows insights into evidence about outcomes in another source.
2. Reconciling of sources of evidence—where results differ in similar situations, these will be further examined to find explanations for these differences.
3. Adjudication of sources of evidence—centred on methodological strengths or weaknesses.
4. Consolidation of sources of evidence—where different outcomes occur in similar contexts, a reason can be developed as to how and why these outcomes happen differently.

In all realist reviews, there is always a risk that the review team will be swamped by the sheer breadth and detail they could cover. As is usual and expected practice in realist reviews (as seen in Item 3 of the RAMESES (Realist And MEta-narrative Evidence Syntheses: Evolving Standards) quality standards for realist syntheses: https://www.ramesesproject.org/media/RS_qual_standards_researchers.pdf), we will progressively focus the review.

This progressive focusing will help prioritise those aspects of the programme theory and/or CMOCs that are most important to stakeholders and/or provide the most useful findings that can be more readily actioned in the NHS. Such issues will be brought to stakeholder group meetings and combined with feedback and advice from the content and methodological expertise of the project team.

During the review, we will seek out data on costs and sustainability. Where such data exist, if possible and relevant we will develop CMOCs for these aspects. Any such findings we develop on costs and sustainability of integrated palliative and heart failure services will be provided at the last two stakeholder meetings along with recommendations for commissioners and providers.

### Objective 2

To provide recommendations for commissioners, practitioners, patients, informal carers and the public.

Our programme theory will be used to provide recommendations for the optimal configuration and implementation of integrated palliative care and heart failure services across those NHS organisations currently implementing or wishing to implement an integrated palliative care and heart failure intervention. We will coproduce recommendations with our key stakeholders (commissioners, local, national, international content experts, multidisciplinary practitioners, patients and the public) as outlined below under our dissemination strategy, to ensure they are feasible, acceptable and meaningful in practice. Recommendations will also inform specific guidelines for local, national and international professional bodies,^34 35^ which recommend an integrated palliative care and heart failure approach, on what works, for whom, how, why and in what contexts to bring about the desired outcomes.

## Ethics and dissemination

### Dissemination

Our dissemination strategy will build on the coproduction approach (involving PPI) that we have used throughout the synthesis process, which has included stakeholder involvement and engagement during the development of this research proposal and review process. Our approach will be consolidative, appreciating the varied forms of knowledge required to generate findings that are useful for informing decision making for policy, practice and public audiences^36^ follows:

1. Policy-makers, decision-makers and commissioners of heart failure services.
2. NHS leaders, managers and practitioners involved in palliative care and heart failure provision.
3. Members of the public, including those impacted by advanced heart failure.

Different strategies will be employed based on the needs of each audience as follows:

1. Academic outputs, that is, journal publications.
2. A coproduced ‘How to’ user guide that outlines practical advice to optimise, tailor and implement interventions designed to integrate palliative care and heart failure that takes the local context into consideration.
3. User-friendly summaries of the synthesis findings using short animations to creatively convey complex findings. The intended impact of this creative approach is to widen the reach of the ‘headline’ findings of the realist synthesis to relevant stakeholders in a visually engaging way.

We will draw on the expertise within the stakeholder group to identify the key people for disseminating to each audience, ensuring outputs are tailored and relevant for their respective needs.

## Supplementary Material

Reviewer comments

Author's manuscript

## References

[1] World Health Organisation. Who global status report on noncommunicable diseases 2014, 2014. Available: http://www.who.int/nmh/publications/ncd-status-report2014/en/

[2] Ponikowski P, Anker SD, AlHabib KF, et al. Heart failure: preventing disease and death worldwide. ESC Heart Fail 2014;1:4–25. 10.1002/ehf2.12005 28834669

[3] Savarese G, Lund LH. Global public health burden of heart failure. Card Fail Rev 2017;3:7–11. 10.15420/cfr.2016:25:2 28785469 PMC5494150

[4] Ambrosy AP, Fonarow GC, Butler J, et al. The Global Health and Economic Burden of Hospitalizations for Heart Failure. J Am Coll Cardiol 2014;63:1123–33. 10.1016/j.jacc.2013.11.053 24491689

[5] Cook C, Cole G, Asaria P, et al. The annual global economic burden of heart failure. Int J Cardiol 2014;171:368–76. 10.1016/j.ijcard.2013.12.028 24398230

[6] Morrison RS, Dietrich J, Ladwig S, et al. Palliative care consultation teams cut hospital costs for Medicaid beneficiaries. Health Aff 2011;30:454–63. 10.1377/hlthaff.2010.0929 21383364

[7] Gonzalez-Jaramillo V, Fuhrer V, Gonzalez-Jaramillo N, et al. Impact of home-based palliative care on health care costs and hospital use: a systematic review. Palliat Support Care 2021;19:474–87. 10.1017/S1478951520001315 33295269

[8] Sahlen K-G, Boman K, Brännström M. A cost-effectiveness study of person-centered integrated heart failure and palliative home care: based on a randomized controlled trial. Palliat Med 2016;30:296–302. 10.1177/0269216315618544 26603186

[9] End of Life Care. Report by the Comptroller and Auditor General [HC 1043 Session 2007-2008. London: National Audit Office, 2008.

[10] Doherty LC, Fitzsimons D, McIlfatrick SJ. Carers' needs in advanced heart failure: a systematic narrative review. Eur J Cardiovasc Nurs 2016;15:203–12. 10.1177/1474515115585237 25922473

[11] Broese van Groenou MI, De Boer A. Providing informal care in a changing society. Eur J Ageing 2016;13:271–9. 10.1007/s10433-016-0370-7 27610055 PMC4992501

[12] Fitzsimons D, Doherty LC, Murphy M, et al. Inadequate communication exacerbates the support needs of current and bereaved caregivers in advanced heart failure and impedes shared decision-making. J Cardiovasc Nurs 2019;34:11–19. 10.1097/JCN.0000000000000516 30157055

[13] Datla S, Verberkt CA, Hoye A, et al. Multi-Disciplinary palliative care is effective in people with symptomatic heart failure: a systematic review and narrative synthesis. Palliat Med 2019;33:1003–16. 10.1177/0269216319859148 31307276

[14] Siouta N, van Beek K, Preston N, et al. Towards integration of palliative care in patients with chronic heart failure and chronic obstructive pulmonary disease: a systematic literature review of European guidelines and pathways. BMC Palliat Care 2016;15:18. 10.1186/s12904-016-0089-4 26872741 PMC4752742

[15] Rogers JG, Patel CB, Mentz RJ, et al. Palliative care in heart failure. J Am Coll Cardiol 2017;70:331–41. 10.1016/j.jacc.2017.05.030 28705314 PMC5664956

[16] O'Donnell AE, Schaefer KG, Stevenson LW, et al. Social Worker-Aided palliative care intervention in high-risk patients with heart failure (SWAP-HF): a pilot randomized clinical trial. JAMA Cardiol 2018;3:516–9. 10.1001/jamacardio.2018.0589 29641819 PMC6128511

[17] Sobanski PZ, Alt-Epping B, Currow DC, et al. Palliative care for people living with heart failure: European association for palliative care Task force expert position statement. Cardiovasc Res 2020;116:12–27. 10.1093/cvr/cvz200 31386104

[18] Hill L, Prager Geller T, Baruah R, et al. Integration of a palliative approach into heart failure care: a European Society of cardiology heart failure association position paper. Eur J Heart Fail 2020;22:2327–39. 10.1002/ejhf.1994 32892431

[19] Anderson H, Ward C, Eardley A, et al. The concerns of patients under palliative care and a heart failure clinic are not being Met. Palliat Med 2001;15:279–86. 10.1191/026921601678320269 12054145

[20] Seferović PM, Jankowska E, Coats AJS, et al. The heart failure association atlas: rationale, objectives, and methods. Eur J Heart Fail 2020;22:638–45. 10.1002/ejhf.1768 32125085

[21] McIlvennan CK, Allen LA. Palliative care in patients with heart failure. BMJ 2016;353:i1010. 10.1136/bmj.i1010 27079896

[22] Diop MS, Rudolph JL, Zimmerman KM, et al. Palliative care interventions for patients with heart failure: a systematic review and meta-analysis. J Palliat Med 2017;20:84–92. 10.1089/jpm.2016.0330 27912043 PMC5177994

[23] Sahlollbey N, Lee CKS, Shirin A, et al. The impact of palliative care on clinical and patient-centred outcomes in patients with advanced heart failure: a systematic review of randomized controlled trials. Eur J Heart Fail 2020;22:2340–6. 10.1002/ejhf.1783 32176831

[24] Singh GK, Ivynian SE, Davidson PM, et al. Elements of integrated palliative care in chronic heart failure across the care continuum: a scoping review. Heart Lung Circ 2021;31:32–41. 10.1016/j.hlc.2021.08.012 34593316

[25] McConnell T, Diffin J, Fitzsimons D, et al. Palliative care and heart failure: can implementation science help where the evidence alone has failed? Eur J Cardiovasc Nurs 2020;19:190–1. 10.1177/1474515119894215 31840529

[26] Lewin WH, Schaefer KG. Integrating palliative care into routine care of patients with heart failure: models for clinical collaboration. Heart Fail Rev 2017;22:517–24. 10.1007/s10741-017-9599-2 28191605

[27] United Nations Department of Economic and Social Affairs Population Division. World population ageing 2019, 2019. Available: http://www.un.org/esa/population/publications/worldageing19502050/pdf/65executivesummaryspanish.pdf%0Ahttp://link.springer.com/chapter/10.1007/978-94-007-5204-7_6

[28] World Health Organisation. Ageing and health, 2018. Available: https://www.who.int/news-room/fact-sheets/detail/ageing-and-health

[29] Braunschweig F, Cowie MR, Auricchio A. What are the costs of heart failure? Europace 2011;13 Suppl 2:ii13–17. 10.1093/europace/eur081 21518742

[30] Hill L, Carson MA, Vitale C. Care plans for the older heart failure patient. Eur Hear J Suppl 2019;21:L32–5. 10.1093/eurheartj/suz243 PMC6926412 31885511

[31] McDonagh TA, Metra M, Adamo M, et al. 2021 ESC guidelines for the diagnosis and treatment of acute and chronic heart failure. Eur Heart J 2021;42:3599–726. 10.1093/eurheartj/ehab368 34447992

[32] Wong G, Greenhalgh T, Westhorp G, et al. RAMESES publication standards: realist syntheses. BMC Med 2013;11:21. 10.1186/1741-7015-11-21 23360677 PMC3558331

[33] Emmel N, Greenhalgh J, Manzano A. Doing realist research. London: Sage, 2018.

[34] National Institute for Care and Excellence (NICE). Chronic heart failure in adults. NICE Quality Standard [QS9, 2011. 30645061

[35] Braun LT, Grady KL, Kutner JS, et al. Palliative care and cardiovascular disease and stroke: a policy statement from the American heart Association/American stroke association. Circulation 2016;134:e198–225. 10.1161/CIR.0000000000000438 27503067

[36] Bowen S, Graham I. Integrated Knowledge Translation. In: Knowledge translation in health care. John Wiley & Sons, 2013: 14–23.

