# Supplementary material for: Integrating palliative care and heart failure: a protocol for a realist synthesis (PalliatHeartSynthesis)
Source: BMJ Open. 2022 Jan 7;12(1):e058848. doi: 10.1136/bmjopen-2021-058848 (PMC8744113; doi:10.1136/bmjopen-2021-058848)
Supplement: Author's manuscript [file bmjopen-2021-058848.draft_revisions.pdf]

BMJ Open is committed to open peer review. As part of this commitment we make the peer review history of every article we publish publicly available.

When an article is published we post the peer reviewers' comments and the authors' responses online. We also post the versions of the paper that were used during peer review. These are the versions that the peer review comments apply to.

The versions of the paper that follow are the versions that were submitted during the peer review process. They are not the versions of record or the final published versions. They should not be cited or distributed as the published version of this manuscript.

BMJ Open is an open access journal and the full, final, typeset and author-corrected version of record of the manuscript is available on our site with no access controls, subscription charges or pay-per-view fees (http://bmjopen.bmj.com).

If you have any questions on BMJ Open's open peer review process please email info.bmjopen@bmj.com

BMJ Open

Integrating Palliative Care and Heart Failure: a protocol for a realist synthesis (PalliatHeartSynthesis)

Journal:	*BMJ Open*
Manuscript ID	bmjopen-2021-058848
Article Type:	Protocol
Date Submitted by the Author:	29-Oct-2021
Complete List of Authors:	McConnell, Tracey ; Queen's University Belfast School of Nursing and Midwifery; Marie Curie Hospice Belfast<br>Burden, John; British Heart Foundation, Patient and Public Involvement Group<br>Duddy, Claire; University of Oxford, Nuffield Department of Primary Care Health Sciences<br>Hill, Loreena; Queen's University Belfast, School of Nursing and Midwifery<br>Howie, Clare; Queen's University Belfast, School of Nursing and Midwifery<br>Jones, Bob; British Heart Foundation, Patient and Public Involvement Group<br>Ruane, Bob; British Heart Foundation, Patient and Public Involvement Group<br>Wong, Geoff; Oxford University, Nuffield Department of Primary Care Health Sciences<br>Reid, Joanne; Queen's University Belfast, School of Nursing and Midwifery
Keywords:	Heart failure < CARDIOLOGY, PALLIATIVE CARE, Organisation of health services < HEALTH SERVICES ADMINISTRATION & MANAGEMENT

SCHOLARONE™
Manuscripts

BMJ

I, the Submitting Author has the right to grant and does grant on behalf of all authors of the Work (as defined in the below author licence), an exclusive licence and/or a non-exclusive licence for contributions from authors who are: i) UK Crown employees; ii) where BMJ has agreed a CC-BY licence shall apply, and/or iii) in accordance with the terms applicable for US Federal Government officers or employees acting as part of their official duties; on a worldwide, perpetual, irrevocable, royalty-free basis to BMJ Publishing Group Ltd ("BMJ") its licensees and where the relevant Journal is co-owned by BMJ to the co-owners of the Journal, to publish the Work in this journal and any other BMJ products and to exploit all rights, as set out in our licence.

The Submitting Author accepts and understands that any supply made under these terms is made by BMJ to the Submitting Author unless you are acting as an employee on behalf of your employer or a postgraduate student of an affiliated institution which is paying any applicable article publishing charge ("APC") for Open Access articles. Where the Submitting Author wishes to make the Work available on an Open Access basis (and intends to pay the relevant APC), the terms of reuse of such Open Access shall be governed by a Creative Commons licence – details of these licences and which Creative Commons licence will apply to this Work are set out in our licence referred to above.

Other than as permitted in any relevant BMJ Author's Self Archiving Policies, I confirm this Work has not been accepted for publication elsewhere, is not being considered for publication elsewhere and does not duplicate material already published. I confirm all authors consent to publication of this Work and authorise the granting of this licence.

For peer review only

Integrating Palliative Care and Heart Failure: a protocol for a realist synthesis (PalliatHeartSynthesis)

Tracey McConnell[1,2], John Burden[3], Claire Duddy[4], Loreena Hill[1], Clare Howie[1], Bob Jones[3], Bob Ruane[3], Geoff Wong[4] & Joanne Reid[1].

[1]School of Nursing and Midwifery, Queen's University Belfast, UK

[2] Marie Curie Marie Curie Hospice, Belfast, Kensington Road, Belfast BT5 6NF

[3]British Heart Foundation London Heart Failure Group Member, UK

[4]Nuffield Department of Primary Care Health Sciences, Radcliffe Observatory Quarter, Oxford, UK

Corresponding author: Tracey McConnell, School of Nursing and Midwifery, Queen's University Belfast, UK, Marie Curie Marie Curie Hospice, Belfast, Kensington Road, Belfast BT5 6NF, t.mcconnell@qub.ac.uk, 07549437132

Abstract

Introduction

Heart failure affects over 26 million people worldwide with prevalence expected to grow due to an ageing global population. Palliative care can address the holistic needs of patients with heart failure, and integrated palliative care in heart failure management has been indicated to improve outcomes for patients. Despite known benefits for integrated palliative care in heart failure management, implementation is poor across the majority of global health services. Recent systematic reviews have identified the benefits of integrating palliative care into heart failure management and highlighted barriers to implementation. However, there was heterogeneity in terms of countries, healthcare settings, delivery by differing staff across multidisciplinary teams, modes of delivery and different intervention components.

Methods & Analysis

The aim of this study is to identify how integrated palliative care and heart failure interventions produce desired outcomes, in which contexts, and for which patients. We will undertake a realist synthesis to identify this, using Pawson's five iterative steps. We will

recruit an international stakeholder group comprised of healthcare providers and patients with heart failure to advise and provide feedback throughout the review. Our initial realist programme theory sets out the necessary steps needed to accomplish the final intended outcome(s) from the implementation of integrated palliative care and heart failure. This initial programme theory will be shaped through an iterative process of testing and refinement.

Ethics and dissemination.

Ethical approval is not required for this study. With our stakeholder group, we will co-produce a user guide that outlines practical advice to optimise, tailor and implement interventions designed to integrate palliative care and heart failure, taking into consideration local context, alongside user-friendly summaries of the synthesis findings using short animations to convey complex findings. We will draw on the expertise within the stakeholder group to identify key stakeholders for disseminating to relevant audiences, ensuring outputs are tailored for their respective needs.

Prospero Registration number CRD42021240185.

Article Summary: Strengths and limitations of this study

- This is the first realist synthesis examining the integration of palliative care into routine heart failure management.
- This synthesis will be supported by a large multidisciplinary, international stakeholder group of policy, practice and patient experts to maximise the potential for widespread implementation, reduce healthcare costs, and improve outcomes for patients and informal carers
- Potential limitations include lack of relevance and rigour in the available literature

Keywords: Heart failure, palliative care, organisation of health services

Word count: 3924 words

Figures:2

Introduction

Background

Cardiovascular disease is the most common cause of death worldwide [1]. Heart failure characterises the final phenotype of many cardiovascular diseases, estimated to currently affect at least 26 million people worldwide [2], with this number expected to grow due to ageing populations [3]. Patients with advanced heart failure or those classified as experiencing New York Heart Association (NYHA) Class III or Class IV symptoms, account for over one million hospitalisations per year in the United States and Europe [4], with an overall estimated economic cost of $108 billion per annum [5]. Integrating palliative care with routine management of heart failure has been shown to significantly reduce healthcare costs overall compared to usual care [6] and significantly reduces the number of hospital visits and duration of in-patient stays [7,8]. The National Audit Office review of end of life care recommended palliative care for patients with heart failure, due to a possible cost savings by reducing utilisation of acute services [9].

There is evidence of improved patient and family outcomes when palliative care is integrated in advanced heart failure management. A review of carers' needs identified that integrated palliative care in heart failure management led to an improvement in satisfaction with care from both the patient and their informal carer [10]. Informal carers are typically defined as those who provide unpaid care to individuals with whom they have a relationship, i.e. family members or spouses [11]. Informal carers are crucial to facilitating independent living and supporting quality of life for patients with heart failure and palliative care can address caregivers' needs and help them care for their loved one [12]. Integrated palliative care in heart failure management can benefit quality of life, symptom burden and levels of depression in patients with the condition [13].

Integrated palliative care aims to achieve continuity of care by integrating administrative, organizational, and clinical services that make up the patients care network [14]. Examples of integrated palliative care and heart failure interventions include collaborations and shared goal setting between palliative care and clinical cardiology teams to ameliorate symptoms with palliative care goals. alongside heart failure management [15]. In addition social work led palliative care services alongside heart failure management [16] aimed to improve the physical, psychological, social, spiritual, and end of life outcomes of patients. In 2020, the European Association for Palliative Care Task Force [17] concluded that the inclusion of

For peer review only

palliative care within the regular clinical framework for people with heart failure provides improvement in quality of life as well as comfort and dignity. This was echoed in a position paper by the Heart Failure Association of the European Society of Cardiology [18] who stated that many patients with heart failure would benefit from earlier integration of a palliative approach into the care provided by the multidisciplinary team involved.

However, although two decades have passed since the first publication on the benefits of palliative care for patients with advanced heart failure [19], the Heart Failure Association (HFA) Atlas identified only 10 out of 42 European countries with designated palliative care units for patients with advanced heart failure [20]. The poor integration of palliative care into advanced heart failure management can be explained by a number of factors, including uncertainty around the heart failure disease trajectory and complexities of communicating this uncertainty to patient and family members. Heart failure is an unpredictable illness, with periods of stability of symptoms, interjected with numerous exacerbations, of which many can lead to a temporary improvement in health status; others can lead to a progressive decline towards death. Many patients with advanced heart failure overestimate survival by up to 40%, further adding to cardiology providers' reluctance to initiate difficult conversations around prognosis. This difficulty is further compounded by lack of patient and practitioner knowledge around what palliative care is; misunderstanding that palliative care equals end-of-life care only. The fragmentation of inpatient and outpatient services also creates a barrier to the holistic need's assessment required for an integrated palliative care approach [21].

Overview of existing evidence

Until recently, the lack of evidence from clinical trials demonstrating benefits of palliative care for people with advanced heart failure posed an additional barrier. However, there has been an exponential increase in published literature since the turn of the century, increasing from 10 publications on average in 2000 to over 100 publications per year in 2017 (23). McIlvennan and Allen [21] published a review summarising the evolving role of palliative care for patients with heart failure, along with the barriers and opportunities for its integration into routine practice. Findings from the review highlighted the need for evidence on how best to integrate palliative care and heart failure given the cultural and environmental differences in how palliative care services are delivered. Three systematic reviews of palliative care interventions for patients with heart failure by Diop et al [22], Datla et al [13] and Sahlollbey et al [23] all highlighted the benefits of palliative care in heart failure management for

For peer review only

patient-centred outcomes and reducing hospital utilisation. A recent scoping review examining elements of integrated palliative care in heart failure management[24] identified the need for a multidisciplinary approach to integration and for cardiology staff to champion the benefits of palliative care. This review also highlighted the need for research with robust theoretical underpinnings given the complex behaviour changes required for sustaining integrated care in practice.

A recent editorial [25] exploring the phenomenon of inconsistent implementation of integrated palliative care and heart failure interventions proposed a realist approach could provide a sound theoretical understanding of the barriers and facilitators to routine implementation. The research to date has focused on “trying to evidence effectiveness through a linear cause and effect approach, which fails to ignore the messy, non-linear world of real-life practice” ([25], p.1). Overall, Datla et al. [26] concluded that there is no clear consensus around: 1) the core components of integrated palliative care and heart failure interventions, 2) the ideal configuration for the multi-disciplinary team, and 3) the most effective service provision model to ensure that generalist and/or specialist palliative care is tailored to patient needs

The issue of heterogeneity was further highlighted in a narrative literature review aimed at identifying the key characteristics of integrated palliative care and heart failure interventions [27]. Of the nine studies included, all integrated palliative care and heart failure interventions were implemented in different countries with different models of health service provision for citizens (USA, Sweden, Hong Kong), different settings (inpatient, outpatient and home-based), delivered by a heterogenous mix of multidisciplinary teams (cardiologists, heart failure-nurses, General Practitioners, community nurses, occupational therapists), using different modes of delivery (face-to-face, telemedicine), and involving different intervention components (symptom management, advance care planning). Therefore, we still do not know which intervention produces the best outcomes for patients and their informal carers (what works: specialist vs primary care etc.?), when best to initiate palliative care (for whom; at what stage in the disease trajectory?), or the optimal delivery method (in what circumstances; required infrastructure, staff competencies etc.?).

Rationale explaining why this research is important now.

Globally the population is living longer than ever before, and there are now 703 million individuals aged 65 or over in the world [28]. Projections estimate that those over 60 years

For peer review only

will increase from 12% of the world's population to 22% by 2050, with 1 in 6 individuals in the world being aged 60 or over by 2030 [29]. Although we can celebrate this achievement in life expectancy, it comes with significant challenges for healthcare now and in the future. Older people have complex health needs, with on average 4.5 comorbidities. Heart failure often dominates their physical and psychological needs [21], along with being the costliest aspect of their care due to high rates of hospitalisation and futile pharmaceutical and surgical interventions as their heart failure progresses [30]. Older people with advanced heart failure have undeniably had their needs overlooked, with calls for more attention to, and research for this vulnerable group to ensure they receive appropriate, effective treatment and care [21,31]. The 2021 European Society of Cardiology (ESC) Guidelines for the diagnosis and treatment of acute and chronic heart failure highlighted the need for studies to determine specific options for palliative care in the treatment of heart failure [32].

Although we have some promising examples of integrated palliative care and heart failure interventions [15,16], there is heterogeneity in terms of countries, healthcare settings, delivery by mix of multidisciplinary teams, modes of delivery and different intervention components [33]. Hence, this review is vital for identifying which model works best, for whom, or in what circumstances.

Aims and objectives

Aim

To understand how integrated palliative care and heart failure interventions may work in different healthcare settings e.g. inpatient/outpatient, and for which groups of people, so we can recommend strategies to maximise the potential for widespread implementation, reduce healthcare costs, and improve QoL for patients and informal carers

Objectives

1. To conduct a realist synthesis to build an understanding of which integrated palliative care and heart failure interventions work best, in which contexts and for which patients and informal carers
2. To co-produce recommendations with an expert stakeholder group, to maximise potential for widespread implementation through a user guide for healthcare providers and user friendly summaries for patients and the public.

For peer review only

Review questions

1. What are the mechanisms by which integrated palliative care and heart failure interventions work in order to produce their intended outcomes?

2. What are the contexts, which determine whether integrated palliative care and heart failure interventions produce their intended or unintended outcomes?

3. In what settings are integrated palliative care and heart failure interventions likely to be effective?

For peer review only

Methods and Analysis

Objective 1. Conduct a realist synthesis

This realist synthesis will follow Pawson's (2005) five iterative steps for realist synthesis (see figure 1). Realist syntheses are particularly suited to research on integrated palliative care and heart failure as they focus on making sense of the contextual factors that determine the outcomes of an intervention. Like other interventions that seek to propagate behavioural change, implementation of integrated palliative care and heart failure is highly context dependent, i.e. implementation of the same intervention will vary in its success depending, for example, on who delivers it and how it is delivered, the characteristics of the health care professionals involved, the circumstances surrounding it, and the tools and techniques used. Research designs that seek to "strip away" these contexts limit our understanding of "how, when and for whom" the intervention will be effective. A realist synthesis takes context as central to any explanation by exploring how an intervention manipulates context to trigger mechanisms that cause behavioural change.

The explanation building will start with the development of an initial programme theory of "how integrated palliative care and heart failure interventions produce desired outcomes (or not)". To achieve this, our preliminary initial realist programme theory sets out the necessary steps needed to accomplish the final intended outcome(s) from implementation of integrated palliative care and heart failure (see figure 2). This initial programme theory will be shaped through an iterative process of testing - i.e. parts of it are confirmed, refuted or refined against a range of relevant data from existing literature (see steps 1-5 below). This protocol has been registered with PROSPERO. The realist synthesis will adhere to the relevant quality and publication standards [33]. Figure 1 provides the project overview and key steps are outlined below in more detail.

Figure 1. Project flow diagram using Pawson's (2005) five iterative steps

INSERT FIGURE 1 HERE

For peer review only - http://bmjopen.bmj.com/site/about/guidelines.xhtml

Patient and public involvement

We will consult with UK wide and international stakeholders with both content and lived experience expertise, including NHS managers, and healthcare professionals (HCPs) involved in the delivery of palliative care and heart failure management, relevant charities, and patient and public involvement (PPI). Our initial programme theory will be refined throughout the course of the project based on regular consultation, via five virtual meetings and email correspondence with our stakeholder group. Our stakeholders, including PPI will also provide their perspectives on findings and recommendations, provide support with dissemination, and contribute to all project materials to ensure they meet the needs of patients and the wider public.

Step 1: Locate existing theories

The purpose of this step is to locate existing theories that explain why, how, in what contexts, for whom and to what extent does integrated palliative care and heart failure work to bring about improved outcomes for advanced heart failure patients and their informal carers. While we have already established there is limited theory underlying integrated palliative care and heart failure interventions, the realist synthesis approach allows for the literature net to be cast wider to include literature from other fields and other professions where potentially shared mechanisms may be in operation.

To identify these theories, we shall iteratively: a) consult with key content experts in our stakeholder group, and b) informally search the literature to identify existing theories. The informal searches conducted in step 1 differ from the more formal searching that will be carried in step 2 as their purpose is to quickly identify the kinds of theory that may be relevant; thus exploratory and informal search methods including, citation tracking and snow-balling based on known existing studies will be used. Once the theories have been identified, we shall build an initial programme theory (drawing on the preliminary initial programme theory- Refer Figure 2) to test in the synthesis. Programme theory development will necessitate iterative discussions within the research team to bring together the different theories into an initial programme theory.

For peer review only

Figure 2. Preliminary initial programme theory

INSERT FIGURE 2 HERE

Step 2: search for evidence

Formal search

The goal of Step 2 is to find a body of relevant literature in order to further develop and refine the initial programme theory developed in Step 1. The searches will be designed, piloted and carried out by an experienced Information Specialist (CD) with experience of carrying out iterative searches for realist synthesis. We will search academic databases including CINAHL, PsycINFO, PubMed, MEDLINE, AMED and EMBASE. A search for grey literature will also be carried out in sources including Google, OpenGrey, NICE, and Health Management Information Consortium (HMIC). Citation searching will also be undertaken including 'cited by' searches and searches of citations in the reference lists of relevant documents. We shall also ask the research team and steering group to identify any literature they may think is relevant. The databases will be searched using free text keywords and controlled vocabulary where appropriate, using terms such as advanced heart failure AND palliative care. The strategy will be informed by our informal scoping searches, search strategies employed in existing reviews and knowledge of the project team. Any literature that is likely to provide conceptually rich data, including grey literature, will be considered for inclusion in the synthesis.

For peer review only

Screening

When screening the identified literature, the inclusion (table 1) and exclusion criteria will be deliberately broad as we seek to identify quantitative, qualitative, mixed-methods and non-empirical documents.

Table 1. Inclusion criteria for identified literature

**Categories**	**Inclusion criteria**
Document types	All documents focused on palliative care for advanced heart failure patients.
Study design	All study designs. Non-empirical data (e.g. from opinion/commentary pieces) which help direct/shape theory development.
Types of settings	All documents about inpatient or outpatient or home-based care settings.
Types of participant	All adult patients (18 years and over). The majority of research has involved patients with advanced heart failure (defined as NYHA class III and IV).
Types of intervention	Any combination of palliative care strategies for the management of patients with advanced heart failure.
Outcome measures	All integrated palliative care and heart failure related outcome measures.

Table 1 shows the categories and details for inclusion criteria for identified literature.

The screening process will be piloted with small samples of documents until agreement on the application of the inclusion criteria is reached within the research team. CH will undertake screening of all titles and abstracts, and TM will independently review a 10% random sample of retrieved citations for quality control. If there are disagreements these will be resolved through discussion between members of the research team. In the event of disagreements remaining, these will be discussed and resolved by majority vote amongst the project research team. Documents included after title and abstract screening will then be screened again against inclusion criteria based on the full text. The same quality assurance process as described above will be used.

For peer review only

Additional searching

A vital part of conducting a realist synthesis involves the option to search for additional data to explain particular parts of the programme theory. Therefore, more searches will be conducted if we need more data to develop and test specific areas of the programme theory. Based on our understanding of integrated palliative care and heart failure to date, these could include areas like clinician/patient/informal carers attitude towards and understanding of palliative care, non-specialist palliative care education and training, having difficult conversations. These additional topics will increase the quantity of relevant data available for us to test the programme theory. The searches will be developed, piloted and refined by the Information Specialist (CD), along with the research team. These searches will differ from the 'formal searches' outlined above through being more exploratory and purposive, and from a range of different disciplines. Each additional search instigated, along with the inclusion and exclusion criteria, will be discussed by the research team to ensure alignment with objectives.

Step 3: Article selection

We are aware that there will be evidence that is of variable quality. However, it is because of this potential challenge that we have chosen to use a realist review approach. For example, full text documents will be selected based on relevance (whether data can contribute to theory building and/or testing) and rigour (whether methods used to generate the relevant data are credible and trustworthy). Assessments will also be made of the plausibility and coherence at the level of the programme theory [34]. To ensure consistency, a random sample of 10% of included papers will be assessed for relevance and discussed between TM and CH. CH will make decisions on the relevance and rigour of the remaining 90%. In the event of any uncertainty, the review team will discuss the relevance or rigour of the articles and any disagreements will be resolved through research team member discussions. A majority vote among the research team will be used to resolve any remaining disagreements.

Step 4: Extracting and organising data

CH will undertake organisation and data extraction. Full texts of the included articles will be imported into NVivo (a qualitative data management software tool). Relevant sections of texts relating to one or more parts of the programme theory will be coded in NVivo firstly by conceptual 'themes' and then as the synthesis progresses these will be developed into context-mechanism-outcome (CMO) configurations (see Step 5 below), the appropriateness

For peer review only

of which is outlined in the literature [33] . Data on the characteristics of the documents will be extracted separately into an Excel spreadsheet. As a quality assurance process, a random 10% of the coding will be independently checked by JR, with any disagreement resolved by discussion within the team.

Step 5: Synthesis the evidence and drawing conclusions

A realist logic of analysis will be used to analyse the data from included documents. Interpretive cross-case comparison will be used to understand and explain how and why actual outcomes have happened e.g. by comparing integrated palliative care and heart failure interventions that have been successful against those which have not, in order to understand how context has influenced reported findings [33]. CH will be supported by GW, CD and TM to undertake this step using the following reasoning processes typically used in synthesising evidence in realist synthesis:

a) Juxtaposition of sources of evidence e.g. where evidence about behaviour change in one source allows insights into evidence about outcomes in another source

b) Reconciling of sources of evidence – where results differ in similar situations, these will be further examined to find explanations for these differences

c) Adjudication of sources of evidence – centred on methodological strengths or weaknesses

d) Consolidation of sources of evidence – where different outcomes occur in similar contexts, a reason can be developed as to how and why these outcomes happen differently.

In all realist reviews there is always a risk that the review team will be swamped by the sheer breadth and detail they could cover. As is usual and expected practice in realist reviews (as seen in Item 3 of the RAMESES quality standards for realist syntheses: https://www.ramesesproject.org/media/RS_qual_standards_researchers.pdf), we will progressively focus the review.

This progressive focussing will help prioritise those aspects of the programme theory and/or CMOCs that are most important to stakeholders and/or provide the most useful findings that can be more readily actioned in the NHS. Such issues will be brought to stakeholder group meetings and combined with feedback and advice from the content and methodological expertise of the project team.

For peer review only

During the review we will seek out data on costs and sustainability. Where such data exist, if possible and relevant we will develop CMOCs for these aspects. Any such findings we develop on costs and sustainability of integrated palliative and heart failure services will be provided at the last two stakeholder meetings along with recommendations for commissioners and providers.

Objective 2.

To provide recommendations for commissioners, practitioners, patients, informal carers and the public.

Our programme theory will be used to provide recommendations for the optimal configuration and implementation of integrated palliative care and heart failure services across those NHS organisations currently implementing or wishing to implement an integrated palliative care and heart failure intervention. We will co-produce recommendations with our key stakeholders (commissioners, local, national, international content experts, multidisciplinary practitioners, patients and the public) as outlined below under our dissemination strategy, to ensure they are feasible, acceptable, and meaningful in practice. Recommendations will also inform specific guidelines for local, national and international professional bodies [35,36], which recommend an integrated palliative care and heart failure approach, on what works, for whom, how, why and in what contexts to bring about the desired outcomes.

For peer review only

Ethics and Dissemination

Dissemination

Our dissemination strategy will build on the co-production approach (involving PPI) that we have used throughout the synthesis process, which has included stakeholder involvement and engagement during the development of this research proposal and review process. Our approach will be consolidative, appreciating the varied forms of knowledge required to generate findings that are useful for informing decision making for policy, practice, and public audiences [37] follows:

1. Policymakers, decision-makers and commissioners of heart failure services.

2. NHS leaders, managers and practitioners involved in palliative care and heart failure provision

3. Members of the public, including those impacted by advanced heart failure

Different strategies will be employed based on the needs of each audience as follows:

1. Academic outputs, i.e. journal publications
2. A co-produced 'How to' user guide that outlines practical advice to optimise, tailor and implement interventions designed to integrate palliative care and heart failure that takes the local context into consideration.
3. User-friendly summaries of the synthesis findings using short animations to creatively convey complex findings. The intended impact of this creative approach is to widen the reach of the 'headline' findings of the realist synthesis to relevant stakeholders in a visually engaging way.

We will draw on the expertise within the stakeholder group to identify the key people for disseminating to each audience, ensuring outputs are tailored and relevant for their respective needs.

Ethics

Ethical approval is not required for this project and has been confirmed by the Research Governance and Ethics department at Queen's University Belfast.

For peer review only

Author Contributions

Tracey McConnell and Joanne Reid conceived the study. Tracey McConnell and Joanne Reid wrote the original protocol for this study with input from Loreena Hill, Geoff Wong and Claire Duddy. Clare Howie prepared the protocol as a manuscript for publication. John Burden, Bob Jones and Bob Ruane as members of the Patient and Public Involvement group provided input on drafting and editing of this protocol. All authors have read and approved the final manuscript.

Funding

This project is funded by the National Institute for Health Research (NIHR)[NIHR131800 Integrating Palliative Care and Heart Failure: a Realist Synthesis (PalliatHeartSynthesis)]**.**

Disclaimer

The views expressed are those of the authors and not necessarily those of the NIHR or the Department of Health and Social Care.

Competing interests statement

Geoff Wong is Deputy Chair of the United Kingdom's National Institute of Health Research Health Technology Assessment Prioritisation Committee: Integrated Community Health and Social Care Panel (A) and a member of Methods Group (A). All other authors declare they have no competing interests.

For peer review only

References

1 World Health Organisation. WHO Global Status Report on Noncommunicable Diseases 2014. 2014. http://www.who.int/nmh/publications/ncd-status-report2014/en/

2 Ponikowski P, Anker SD, AlHabib KF, *et al.* Heart failure: preventing disease and death worldwide. *ESC Hear Fail* 2014;**1**:4–25. doi:10.1002/ehf2.12005

3 Savarese G, Lund LH. Global Public Health Burden of Heart Failure. *Card Fail Rev* 2017;**3**:7–11. doi:10.1007/s11886-002-0048-y

4 Ambrosy, Andrew P, Fonarow, Gregg C, Javed B, *et al.* The Global Health and Economic Burden of Hospitalizations for Heart Failure. *J Am Coll Cardiol* 2014;**63**:1123–33. doi:10.1016/j.jacc.2013.11.053

5 Cook C, Cole G, Asaria P, *et al.* The annual global economic burden of heart failure. *Int J Cardiol* 2014;**171**:368–76. doi:https://doi.org/10.1016/j.ijcard.2013.12.028

6 Morrison RS, Dietrich J, Ladwig S, *et al.* Palliative Care Consultation Teams Cut Hospital Costs For Medicaid Beneficiaries. *Health Aff* 2011;**30**:454–63. doi:10.1377/hlthaff.2010.0929

7 Gonzalez-Jaramillo V, Fuhrer V, Gonzalez-Jaramillo N, *et al.* Impact of home-based palliative care on health care costs and hospital use: A systematic review. *Palliat Support Care* 2021;**19**:474–87. doi:DOI: 10.1017/S1478951520001315

8 Sahlen K-G, Boman K, Brännström M. A cost-effectiveness study of person-centered integrated heart failure and palliative home care: Based on a randomized controlled trial. *Palliat Med* 2015;**30**:296–302. doi:10.1177/0269216315618544

9 End of Life Care. Report by the Comptroller and Auditor General [HC 1043 Session 2007-2008] London (2008) National Audit Office.

10 Doherty LC, Fitzsimons D, McIlfatrick SJ. Carers' needs in advanced heart failure: A systematic narrative review. *Eur J Cardiovasc Nurs* 2016;**15**:203–12. doi:10.1177/1474515115585237

11 Broese van Groenou MI, De Boer A. Providing informal care in a changing society. *Eur J Ageing* 2016;**13**:271–9. doi:10.1007/s10433-016-0370-7

12 Fitzsimons D, Doherty LC, Murphy M, *et al.* Inadequate Communication Exacerbates

For peer review only

the Support Needs of Current and Bereaved Caregivers in Advanced Heart Failure and Impedes Shared Decision-making. *J Cardiovasc Nurs* 2019;**34**.https://journals.lww.com/jcnjournal/Fulltext/2019/01000/Inadequate_Communication_Exacerbates_the_Support.5.aspx

13 Datla S, Verberkt CA, Hoye A, *et al.* Multi-disciplinary palliative care is effective in people with symptomatic heart failure: A systematic review and narrative synthesis. *Palliat Med* 2019;**33**:1003–16. doi:10.1177/0269216319859148

14 Siouta N, van Beek K, Preston N, *et al.* Towards integration of palliative care in patients with chronic heart failure and chronic obstructive pulmonary disease: a systematic literature review of European guidelines and pathways. *BMC Palliat Care* 2016;**15**:18. doi:10.1186/s12904-016-0089-4

15 Rogers JG, Chetan PB, Mentz RJ, *et al.* Palliative Care in Heart Failure. *J Am Coll Cardiol* 2017;**70**:331–41. doi:10.1016/j.jacc.2017.05.030

16 O'Donnell AE, Schaefer KG, Stevenson LW, *et al.* Social Worker–Aided Palliative Care Intervention in High-risk Patients With Heart Failure (SWAP-HF): A Pilot Randomized Clinical Trial. *JAMA Cardiol* 2018;**3**:516–9. doi:10.1001/jamacardio.2018.0589

17 Sobanski PZ, Alt-Epping B, Currow DC, *et al.* Palliative care for people living with heart failure: European Association for Palliative Care Task Force expert position statement. *Cardiovasc Res* 2020;**116**:12–27. doi:10.1093/cvr/cvz200

18 Hill L, Prager Geller T, Baruah R, *et al.* Integration of a palliative approach into heart failure care: a European Society of Cardiology Heart Failure Association position paper. *Eur J Heart Fail* 2020;**22**:2327–39. doi:10.1002/ejhf.1994

19 Anderson H, Ward C, Eardley A, *et al.* The concerns of patients under palliative care and a heart failure clinic are not being met. *Palliat Med* 2001;**15**:279–86. doi:10.1191/026921601678320269

20 Seferović PM, Jankowska E, Coats AJS, *et al.* The Heart Failure Association Atlas: rationale, objectives, and methods. *Eur J Heart Fail* 2020;**22**:638–45. doi:10.1002/ejhf.1768

21 McIlvennan CK, Allen LA. Palliative care in patients with heart failure. *Br Med J*

For peer review only

2016;**353**:i1010. doi:10.1136/bmj.i1010

22 Diop MS, Rudolph JL, Zimmerman KM, *et al.* Palliative Care Interventions for Patients with Heart Failure: A Systematic Review and Meta-Analysis. *J Palliat Med* 2016;**20**:84–92. doi:10.1089/jpm.2016.0330

23 Sahlollbey N, Kai Seng Lee C, Shirin A, *et al.* The impact of palliative care on clinical and patient-centred outcomes in patients with advanced heart failure: a systematic review of randomized controlled trials. *Eur J Heart Fail* 2020;**22**:2340–6.

24 Singh GK, Ivynian SE, Davidson PM, *et al.* Elements of Integrated Palliative Care in Chronic Heart Failure Across the Care Continuum: A Scoping Review. *Hear Lung Circ* 2021;:1–10. doi:10.1016/j.hlc.2021.08.012

25 McConnell T, Diffin J, Fitzsimons D, *et al.* Palliative care and heart failure: Can implementation science help where the evidence alone has failed? *Eur J Cardiovasc Nurs* 2019;**19**:190–1. doi:10.1177/1474515119894215

26 Datla S, Verberkt CA, Hoye A, *et al.* Multi-disciplinary palliative care is effective in people with symptomatic heart failure: A systematic review and narrative synthesis. *Palliat Med* 2019;**33**:1003–16. doi:10.1177/0269216319859148

27 Lewin WH, Schaefer KG. Integrating palliative care into routine care of patients with heart failure: models for clinical collaboration. *Heart Fail Rev* 2017;**22**:517–24. doi:10.1007/s10741-017-9599-2

28 United Nations Department of Economic and Social Affairs Population Division. *World Population Ageing 2019*. 2019. http://www.un.org/esa/population/publications/worldageing19502050/pdf/65executive summary spanish.pdf%0Ahttp://link.springer.com/chapter/10.1007/978-94-007-5204-7_6

29 Ageing and Health. World Health Organisation. 2018.https://www.who.int/news-room/fact-sheets/detail/ageing-and-health

30 Braunschweig F, Cowie MR, Auricchio A. What are the costs of heart failure? *EP Eur* 2011;**13**:ii13–7. doi:10.1093/europace/eur081

31 Hill L, Carson MA, Vitale C. Care plans for the older heart failure patient. *Eur Hear J Suppl* 2019;**21**:L32–5. doi:10.1093/eurheartj/suz243

32 McDonagh TA, Metra M, Adamo M, *et al.* 2021 ESC Guidelines for the diagnosis and treatment of acute and chronic heart failure. *Eur Heart J* 2021;:1–128. doi:10.1093/eurheartj/ehab368

33 Wong G, Greenhalgh T, Westhorp G, *et al.* RAMESES publication standards: realist syntheses. *BMC Med* 2013;**11**:21. doi:10.1186/1741-7015-11-21

34 Emmel N, Greenhalgh J, Manzano A, Monaghan M, Dalkin S. *Doing Realist Research.* London: : Sage 2018.

35 National Institute for Care and Excellence (NICE). Chronic heart failure in adults. NICE Quality Standard [QS9]. 2011.

36 Braun LT, Grady KL, Kutner JS, *et al.* Palliative Care and Cardiovascular Disease and Stroke: A Policy Statement From the American Heart Association/American Stroke Association. *Circulation* 2016;**134**:e198–225. doi:10.1161/CIR.0000000000000438

37 Bowen S, Graham I. Integrated Knowledge Translation. In: *Knowledge Translation in Health Care*. John Wiley & Sons 2013. 14–23.

For peer review only

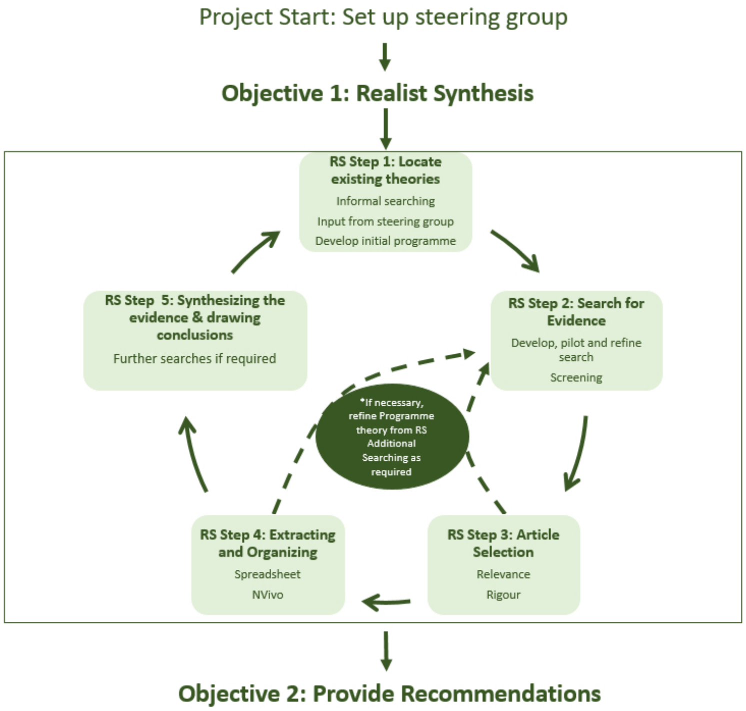

Figure 1. Project flow diagram using Pawson's (2005) five iterative steps

656x476mm (38 x 38 DPI)

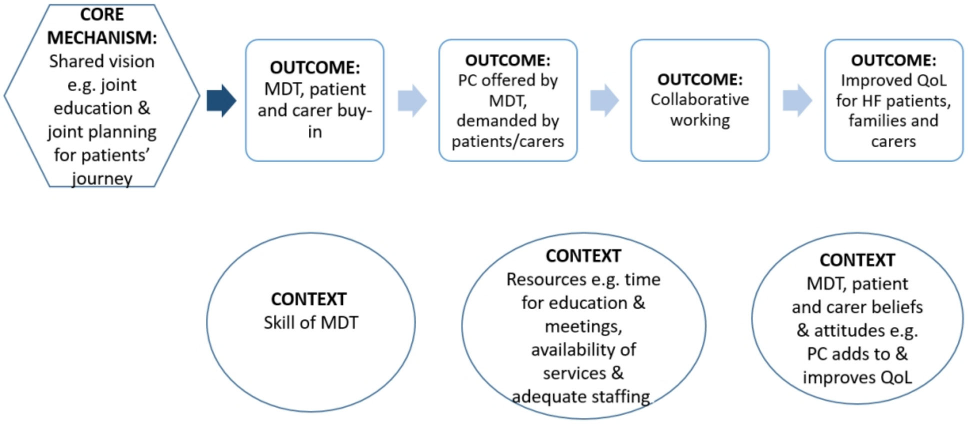

Figure 2. Preliminary initial programme theory

940x425mm (38 x 38 DPI)

Wong et al. BMC Medicine 2013, 11:21
http://www.biomedcentral.com/1741-7015/11/21

BMC Medicine

GUIDELINE Open Access

RAMESES publication standards: realist syntheses

Geoff Wong[1*], Trish Greenhalgh[1], Gill Westhorp[2], Jeanette Buckingham[3] and Ray Pawson[4]

Abstract

Background: There is growing interest in realist synthesis as an alternative systematic review method. This approach offers the potential to expand the knowledge base in policy-relevant areas - for example, by explaining the success, failure or mixed fortunes of complex interventions. No previous publication standards exist for reporting realist syntheses. This standard was developed as part of the RAMESES (Realist And MEta-narrative Evidence Syntheses: Evolving Standards) project. The project's aim is to produce preliminary publication standards for realist systematic reviews.

Methods: We (a) collated and summarized existing literature on the principles of good practice in realist syntheses; (b) considered the extent to which these principles had been followed by published syntheses, thereby identifying how rigor may be lost and how existing methods could be improved; (c) used a three-round online Delphi method with an interdisciplinary panel of national and international experts in evidence synthesis, realist research, policy and/or publishing to produce and iteratively refine a draft set of methodological steps and publication standards; (d) provided real-time support to ongoing realist syntheses and the open-access RAMESES online discussion list so as to capture problems and questions as they arose; and (e) synthesized expert input, evidence syntheses and real-time problem analysis into a definitive set of standards.

Results: We identified 35 published realist syntheses, provided real-time support to 9 on-going syntheses and captured questions raised in the RAMESES discussion list. Through analysis and discussion within the project team, we summarized the published literature and common questions and challenges into briefing materials for the Delphi panel, comprising 37 members. Within three rounds this panel had reached consensus on 19 key publication standards, with an overall response rate of 91%.

Conclusion: This project used multiple sources to develop and draw together evidence and expertise in realist synthesis. For each item we have included an explanation for why it is important and guidance on how it might be reported. Realist synthesis is a relatively new method for evidence synthesis and as experience and methodological developments occur, we anticipate that these standards will evolve to reflect further methodological developments. We hope that these standards will act as a resource that will contribute to improving the reporting of realist syntheses.
To encourage dissemination of the RAMESES publication standards, this article is co-published in the Journal of Advanced Nursing and is freely accessible on Wiley Online Library (http://www.wileyonlinelibrary.com/journal/jan).
Please see related article http://www.biomedcentral.com/1741-7015/11/20 and http://www.biomedcentral.com/1741-7015/11/22

Keywords: realist synthesis, realist review, publication standards

Background

Academics and policymakers are increasingly interested in 'policy-friendly' approaches to evidence synthesis. Such approaches seek to illuminate issues and understand contextual influences on whether, why and how interventions might work [1,2]. A number of different approaches have been used to try to achieve this goal. At present there is lack of clarity on which methods are best suited for which questions or problems and this has been the subject of debate [3-6] and further research [7]. Realist synthesis is a theory-driven approach that is becoming increasingly popular.

* Correspondence: grckwong@gmail.com
[1]Centre for Primary Care and Public Health, Queen Mary University of London, 58 Turner Street, London E1 2AB, UK
Full list of author information is available at the end of the article

BioMed Central
© 2013 Wong et al; licensee BioMed Central Ltd. This is an open access article distributed under the terms of the Creative Commons Attribution License (http://creativecommons.org/licenses/by/2.0), which permits unrestricted use, distribution, and reproduction in any medium, provided the original work is properly cited.

For peer review only

Wong et al. BMC Medicine 2013, 11:21
http://www.biomedcentral.com/1741-7015/11/21

What is a realist synthesis?

In this section we briefly describe the realist synthesis method. The realist research question is often summarized as "What works for whom under what circumstances, how and why?" Realist inquiry is based on a realist philosophy of science and considers the interaction between context, mechanism and outcome. From a realist perspective, intervention X is not thought of as having effect size Y with confidence interval Z. Rather, intervention X (for example, a program introduced by policymakers who seek to create a particular outcome) alters context (for example, by making new resources available), which then triggers mechanism(s), which produce both intended and unintended outcomes. Intervention X may work well in one context but poorly or not at all in another context.

Realist inquiry seeks to unpack the context - mechanism - outcome relationship, thereby explaining examples of success, failure and various eventualities in between. Theoretical explanations of this kind are referred to as "middle-range theories" (that is, ones which "...involve abstraction... but [are] close enough to observed data to be incorporated in propositions that permit empirical testing" [8].

The basis of realist inquiry is a realist philosophy, whose key tenets are as follows:

1. There is a [social] reality that cannot be measured directly (because it is processed through our brains, language, culture and so on), but can be known indirectly.

Realism thus sits, broadly speaking, between positivism ('there is a real world which we can apprehend directly through observation') and constructivism ('given that all we can know has been interpreted through human senses and the human brain, we cannot know for sure what the nature of reality is').

2. Social programs (including complex interventions) may change the macro social context (for example, by introducing legislation). They may also change the resources or opportunities available to participants and, in that sense, change the meso- or micro-level context for those participants.

3. To understand the relationship between context and outcome, realism uses the concept of mechanisms, one definition of which is "...underlying entities, processes, or [social] structures which operate in particular contexts to generate outcomes of interest" [9].

In common with other theory-driven review methods, the realist approach offers the potential for insights that go beyond the narrowly experimental paradigm of the randomized controlled trial [10-12]. It can do so in relation to complex, complicated or simpler interventions (for example, even a simple intervention, such as a drug, is prescribed, dispensed and taken - or not - in a particular social, cultural and economic context).

"Realist synthesis" was first described by Ray Pawson in 2002 [13], updated in an ESRC (Economic and Social Research Council) commissioned monograph in 2004 [14], published as a book in 2006 [1] and summarizsed in a short methods paper in 2005 [15]. Since this paper is deliberately focused on publication standards, we strongly recommend that those unfamiliar with the realist approach consult these or other relevant methodological sources.

A realist synthesis (or realist review - these terms are synonymous) applies realist philosophy to the synthesis of findings from primary studies that have a bearing on a single research question or set of questions. Methodologically, reviewers may begin by eliciting from the literature the main ideas that went into the making of a class of interventions (the program theory). This program theory sets out how and why a class of intervention is thought to 'work' to generate the outcome(s) of interest. The pertinence and effectiveness of each constituent idea is then tested using relevant evidence (qualitative, quantitative, comparative, administrative and so on) from the primary literature on that class of programs. In this testing, the ideas within a program theory are re-cast and conceptualized in realist terms.

For each idea, reviewers seek out the contextual (C) influences that are hypothesized to have triggered the relevant mechanism(s) (M) to generate the outcome(s) (O) of interest. Synthesis consists of comparing 'how the programme was supposed to operate' to the 'empirical evidence on the actuality in different situations' - all along C-M-O lines. Analytic purchase comes from the ability to describe and understand the many contingencies that affect the likelihood of such interventions generating their intended outcomes. This in turn provides guidance about what policy makers or practitioners might put in place to change the context or provide resources in such a way as to most likely trigger the right mechanism(s) to produce the desired outcome.

Why are publication standards needed?

Publication standards are common (and, increasingly, expected) - in health services research - see, for example, CONSORT for randomized controlled trials [16], AGREE for clinical guidelines [17], PRISMA for Cochrane-style systematic reviews [18] and SQUIRE for quality improvement studies [19]. For realist syntheses, publication standards are particularly important as this method is relatively new and concerns have been expressed about the rigor with which some realist reviews have been carried out and reported [20]. Publication standards are needed to ensure that users of reviews are provided with relevant and necessary information to enable them to assess the quality and rigor of a review.

In our experience, there is considerable confusion among researchers, journal editors, peer reviewers and

For peer review only

funders about what counts as a high quality realist review and what, conversely, counts as a flawed review. Even though experts still differ on detailed conceptual methodological issues, the increasing popularity of this method prompted a study to develop baseline standards from which, we anticipate, further developments in theory and methodology of this approach will occur.

Aim

The aim of this paper is to produce preliminary publication standards for realist syntheses.

Methods

The methods we used to develop these reporting standards have already been published [20]. In brief, we purposively recruited an international group of experts to our online Delphi panel. Aiming to achieve maximum variety in the relevant sectors, disciplines and expert perspectives represented, we sought panel members working in realist research, evidence synthesis, publication, reviewer training and health policy. Prior to the start of our Delphi panel, with input from an expert informaticist (JB), we collated and summarized existing literature on the principles of good practice in realist synthesis, created a database of such published syntheses, and built relationships with teams who were undertaking ongoing syntheses. Through discussion within the project team, we considered the extent to which the principles had been followed by published and in-progress reviews, thereby identifying how rigor may be lost and how existing methods could be improved.

Our analysis of existing realist syntheses formed the basis of the briefing materials for the first round of the Delphi panel. In addition, we drew on our collective experience in training and supporting realist syntheses teams and an email discussion list on realist and meta-narrative methodology [21] to further inform the contents of our briefing document. Both the research team and panel members contributed draft items for the publication standards, and these were refined using the online Delphi process as previously described [20]. We ran the Delphi panels between September 2011 and March 2012.

Description of panel and items

In all, we recruited 37 individuals from 27 organizations in 6 countries. These comprised: researchers in public or population health researchers (8); evidence synthesis (6); health services research (8); international development (2); education (2); and also research methodologists (6), publishing (1), nursing (2) and policy and decision making (2). In round 1, 22 Delphi panel members provided suggestions of items that should be included in the publication standards. In rounds 2 and 3 our panel members were asked to rate each potential item for relevance and clarity. The response rates across all items for rounds 2 and 3 were 93% and 89%, respectively. Consensus was reached within three rounds on both the content and wording of 19 items within the publication standards. Table 1 provides an overview of these items.

Scope of the publication standards

These publication standards are intended to help researchers, authors, journal editors, and policy and decision makers to know and understand what should be reported in the write-up of a realist synthesis. They are not intended to provide detailed guidance on how to conduct such a synthesis; for this, we direct interested readers to summary articles [15,22] or various publications on methods [1,11,14,23]. This publication standard applies only to realist syntheses. A list of publication guidelines for other review methods can be found on the EQUATOR Network's website [24], but at present none of these relate specifically to realist syntheses. As part of the RAMESES project we are also developing quality standards and training materials for realist syntheses, which will be submitted as a separate publication. Publication standards for meta-narrative reviews (also covered in the RAMESES project) have been addressed in a separate article.

How to use these publication standards

The layout of this document has drawn on previous methodological publications and, in particular, on the 'Explanations and Elaborations' document of the PRISMA statement [18]. Each item is followed by an example drawn from published reviews and a rationale for its inclusion. The purpose of the example text is to illustrate how an item might be reported in a write up. However, potentially relevant contextual information may have been omitted, so it may be necessary to consult the original paper from which the example text was drawn. The standards set out what might be expected for each item, but authors will still need to exercise judgement about how much information to include. The purpose of the details reported should be to ensure that the description and explanation provided is coherent and plausible, both against the guidance set out within an item and for the overall purpose of the realist synthesis.

While this publication standard is modeled on the PRISMA statement, the items within are not identical. This publication standard, developed to apply only to realist syntheses, has some overlap with the PRISMA statement. Items 1 to 3, 15, 16 and 19 in this statement broadly match the purpose of items 1 to 3, 24, 25 and 27 in the PRISMA statement. For items 4 to 14, while there is some overlap in purpose with some PRISMA statement items, different or additional reporting is needed due to the nature of realist syntheses. Other items (5, 12, 13, 15, 16, 19

For peer review only

Table 1 List of items to be included when reporting a realist synthesis

**TITLE**	
1	In the title, identify the document as a realist synthesis or review
ABSTRACT	
2	While acknowledging publication requirements and house style, abstracts should ideally contain brief details of: the study's background, review question or objectives; search strategy; methods of selection, appraisal, analysis and synthesis of sources; main results; and implications for practice.
INTRODUCTION	
3 Rationale for review	Explain why the review is needed and what it is likely to contribute to existing understanding of the topic area.
4 Objectives and focus of review	State the objective(s) of the review and/or the review question(s). Define and provide a rationale for the focus of the review.
METHODS	
5 Changes in the review process	Any changes made to the review process that was initially planned should be briefly described and justified.
6 Rationale for using realist synthesis	Explain why realist synthesis was considered the most appropriate method to use.
7 Scoping the literature	Describe and justify the initial process of exploratory scoping of the literature.
8 Searching processes	While considering specific requirements of the journal or other publication outlet, state and provide a rationale for how the iterative searching was done. Provide details on all the sources accessed for information in the review. Where searching in electronic databases has taken place, the details should include, for example, name of database, search terms, dates of coverage and date last searched. If individuals familiar with the relevant literature and/or topic area were contacted, indicate how they were identified and selected.
9 Selection and appraisal of documents	Explain how judgements were made about including and excluding data from documents, and justify these.
10 Data extraction	Describe and explain which data or information were extracted from the included documents and justify this selection.
11 Analysis and synthesis processes	Describe the analysis and synthesis processes in detail. This section should include information on the constructs analyzed and describe the analytic process.
RESULTS	
12 Document flow diagram	Provide details on the number of documents assessed for eligibility and included in the review with reasons for exclusion at each stage as well as an indication of their source of origin (for example, from searching databases, reference lists and so on). You may consider using the example templates (which are likely to need modification to suit the data) that are provided.
13 Document characteristics	Provide information on the characteristics of the documents included in the review.
14 Main findings	Present the key findings with a specific focus on theory building and testing.
DISCUSSION	
15 Summary of findings	Summarize the main findings, taking into account the review's objective(s), research question(s), focus and intended audience(s).
16 Strengths, limitations and future research directions	Discuss both the strengths of the review and its limitations. These should include (but need not be restricted to) (a) consideration of all the steps in the review process and (b) comment on the overall strength of evidence supporting the explanatory insights which emerged. The limitations identified may point to areas where further work is needed.
17 Comparison with existing literature	Where applicable, compare and contrast the review's findings with the existing literature (for example, other reviews) on the same topic.
18 Conclusion and recommendations	List the main implications of the findings and place these in the context of other relevant literature. If appropriate, offer recommendations for policy and practice.
19 Funding	Provide details of funding source (if any) for the review, the role played by the funder (if any) and any conflicts of interests of the reviewers.

and 23) in the PRIMSA statement have no equivalent in the RAMESES publication standards for realist reviews.

The order in which items are reported may vary. Realist syntheses are not 'linear' reviews. Some of the processes that are listed may legitimately take place in parallel or have to be revisited at a later date as a review progresses. As a general rule, if a recommended item is excluded from the write-up of a realist synthesis, a justification should be provided.

The RAMESES publication standards for realist syntheses

Item 1: Title

In the title, identify the document as a realist synthesis or review.

For peer review only - http://bmjopen.bmj.com/site/about/guidelines.xhtml

Example

"Human resource management interventions to improve health workers' performance in low and middle income countries: a realist review." [25]

Explanation

Our background searching has shown that some realist reviews are not flagged as such in the title and may also be inconsistently indexed and, hence, are more difficult to locate during searching. The terms 'realist synthesis' and 'realist review' are both in widespread use. We asked our Delphi panel if they had a preferred term - 'realist synthesis' or 'review'. No consensus was reached by our Delphi panel on whether 'review' or 'synthesis' should be the preferred term, and there seemed no good reason to impose one or other term.

Item 2: Abstract

While acknowledging that requirements and house style may differ between journals, abstracts should ideally contain brief details of the study's background, review question or objectives; search strategy; methods of selection, appraisal, analysis and synthesis of sources; main results; and implications for practice.

Example

"Background

Legislation is one of the most powerful weapons for improving population health and is often used by policy and decision makers. Little research exists to guide them as to whether legislation is feasible and/or will succeed. We aimed to produce a coherent and transferable evidence based framework of threats to legislative interventions to assist the decision making process and to test this through the 'case study' of legislation to ban smoking in cars carrying children.

Methods

We conceptualised legislative interventions as complex social interventions and so used the realist synthesis method to systematically review the literature for evidence. 99 articles were found through searches on five electronic databases (MEDLINE, HMIC, EMBASE, PsychINFO, Social Policy and Practice) and iterative purposive searching. Our initial searches sought any studies that contained information on smoking in vehicles carrying children. Throughout the review we continued where needed to search for additional studies of any type that would conceptually contribute to helping build and/or test our framework.

Results

Our framework identified a series of transferable threats to public health legislation. When applied to smoking bans in vehicles; problem misidentification, public support; opposition; and enforcement issues were particularly prominent threats. Our framework enabled us to understand and explain the nature of each threat and to infer the most likely outcome if such legislation were to be proposed in a jurisdiction where no such ban existed. Specifically, the micro-environment of a vehicle can contain highly hazardous levels of second hand smoke. Public support for such legislation is high amongst smokers and non-smokers and their underlying motivations were very similar - wanting to practice the Millian principle of protecting children from harm. Evidence indicated that the tobacco industry was not likely to oppose legislation and arguments that such a law would be 'unenforceable' were unfounded.

Conclusion

It is possible to develop a coherent and transferable evidence based framework of the ideas and assumptions behind the threats to legislative intervention that may assist policy and decision makers to analyse and judge if legislation is feasible and/or likely to succeed." [26]

Explanation

Apart from the title, an abstract is the only source of information accessible to searchers unless the full paper is obtained. The information in it must allow reviewers and/or users to decide if the review is relevant to their needs.

Introduction section

The following items should be reported in the introduction section.

Item 3: Rationale for review

Explain why the review is needed and what it is likely to contribute to existing understanding of the topic area.

Example

"A number of reviews on the subject have tried to examine evidence to improve the operationalization of interventions by CHWs [community health workers], including for child health. Lehmann *et al.* (Reference x1) and Lewin *et al.* (Reference x1) have reviewed evidence on CHW interventions in LMIC [low-middle income countries] and Haines *et al.* (Reference x1) have particularly so for child health. Lewin *et al.* (Reference x1) found lay health workers to be effective in specific areas in child health, when compared to usual care. Haines *et al.* (Reference x1) highlight the contextual nature of CHW's performance. Both caution that CHW interventions are not the panacea for all that ails the health systems in LMIC and that large scale CHW programmes should be initiated with great caution. Both raise questions about the applicability of findings to different settings and about the conditions under which CHW interventions should be implemented." [27]

For peer review only

Wong et al. BMC Medicine 2013, 11:21
http://www.biomedcentral.com/1741-7015/11/21

Explanation
As with all research, a background section explaining what is already known and what the researchers considered to be the 'knowledge gaps' is a helpful orientation.

Item 4: Objectives and focus of review
State the objective(s) of the review and/or the review question(s). Define and provide a rationale for the focus of the review.

Example
"The overriding question for the review was: Does moving from high-poverty neighborhoods to lower-poverty neighborhoods improve health? More specifically: What were the key health outcomes? Who experienced these outcomes? What appeared to be the mechanisms and associated context leading to the outcomes? As the review proceeded, it became clear that one of the only relatively consistent and statistically significant positive health outcomes was an improvement in mental health for adult women, children and adolescent girls. In this paper a review of mental health outcomes of MTO [Moving To Opportunity] is presented, along with some insights about the mechanisms and contexts through which the intervention appears to have impacted mental health." [28]

Explanation
A realist research question contains some or all of the elements of 'What works, how, why, for whom, to what extent and in what circumstances, in what respect and over what duration?' and applies realist logic to address the question (see Item 11).

Because a realist synthesis may generate a large number of avenues that might be explored and explained, and because resources and timescale are invariably finite, the expectation is that the review must be 'contained' by progressively focusing both its breadth (how wide an area?) and depth (how much detail?). This important process may involve discussion and negotiation with, for example, content experts, funders and/or users. It is typical and legitimate for the synthesis' objectives, question and/or the breadth and depth of the review to evolve as the review progresses. How and why it evolved is usually worth reporting.

Methods section
The following items should be reported in the methods section.

Item 5: Changes in the review process
Any changes made to the review that was initially planned should be briefly described and justified.

Example
"As the review progressed we became aware of various data suitability limitations (see Discussion) and the emergence of two prominent demi-regularities prompted us to narrow our review focus to the two candidate theories discussed below." [29]

Explanation
A realist synthesis can (and, in general, should) evolve over the course of the review. For example, changes to the research question or its scope are likely to have an impact on many of the synthesis' subsequent processes. However, this does not mean the synthesis can meander uncontained. An accessible summary of what was originally planned (for example, as described in an initial protocol) and how and why this differed from what was done should be provided as this may assist interpretation.

Item 6: Rationale for using realist synthesis
Explain why realist synthesis was considered the most appropriate method to use.

Example
"Previous reviews sought to understand PR [participatory research] and provide practical recommendations (References x6) and to assess the value of PR to research goals, health status, and systems change (References x6). Nonetheless, the assessment of outcomes remains weak (Reference x4), partly because the methodologies used have generally failed to embrace the complexity of programs or address mechanisms of change (Reference x1). ...

To handle such complexity, we chose a realist approach (Reference x1) because it provides a rationale and tools for synthesizing complex, difficult-to-interpret evidence from community-based programs." [30]

Explanation
Realist synthesis is a theory-driven method that is firmly rooted in a realist philosophy of science. It places particular emphasis on understanding causation (in this case, understanding how programs and policies generate outcomes through human decisions) and how causal mechanisms are shaped and constrained by social context. This makes it particularly suitable for reviews of certain topics and questions - for example, complex social programs that involve human decisions and actions. It also makes realist synthesis *less* suitable than other review methods for certain topics and questions - for example, those which seek primarily to determine the average effect size of a simpler intervention administered in a single or limited range of conditions. In our analysis of 37 published realist syntheses, the most common limitation was inadequate engagement with realist explanatory principles and the implications these have, first, for understanding programs and how they work, and second, for cumulating evidence and explanation.

Some realist syntheses published to date have deliberately adapted the method as first described by Pawson.

For peer review only

Wong *et al. BMC Medicine* 2013, **11**:21
http://www.biomedcentral.com/1741-7015/11/21

Sometimes, adaptations may be entirely justifiable, but at other times they may indicate a poor grasp of realist methodology. To enable judgement to be made on adaptations, the description and rationale for adaptations should be provided. Such information will allow criticism, debate and counter criticism among review teams and users on the suitability of such adaptations, and may well facilitate methodological development.

Item 7: Scoping the literature

Describe and justify the initial process of exploratory scoping of the literature.

Example

"To develop our framework on the threats to the programme theory of public health legislation we started out by conducting a rapid review of broad areas of public health legislation (covering everything from gun amnesties to food labelling) trying to uncover what had been the sticking points in legislation and how (if at all) they had been circumvented. This outline review led to the construction of a provisional framework for reviewing the family of legislative interventions (as described in Figure 1). .... . Beginning with this framework and through discussions (and with reference to other interested stakeholders) we focused on a subset of themes that seemed most relevant in respect to the intervention in question. In our case, we deliberately sought input from the NICE officer seconded to our project." [26]

Explanation

This step is used to build an understanding of the topic area. For example, this step may be used to identify provisional program theories, the names/titles of programs within scope and key authors in the area. Initial attempts to make sense of a topic area may involve informal 'browsing' of the literature and also consulting with experts and stakeholders.

Item 8: Searching process

While considering specific requirements of the journal or other publication outlet, state and provide a rationale for how the iterative searching was done. Provide details on all the sources accessed for information in the synthesis. For example, where electronic databases have been searched, details should include, for example, the name of the database, search terms, dates of coverage and date last searched. If individuals familiar with the relevant literature and/or topic area were contacted, indicate how they were identified and selected.

Example

"...the literature search was iterative and ongoing throughout the project. An initial search was conducted of various academic databases, such as Academic Search Premier, Arts and Humanities Citation Index, Canadian Research Index, as well as through various search engines, such as Prowler, Novanet, Google and Google Scholar. Search terms included: Moving to Opportunity [MTO]; housing intervention; housing mobility; housing health effects; low-

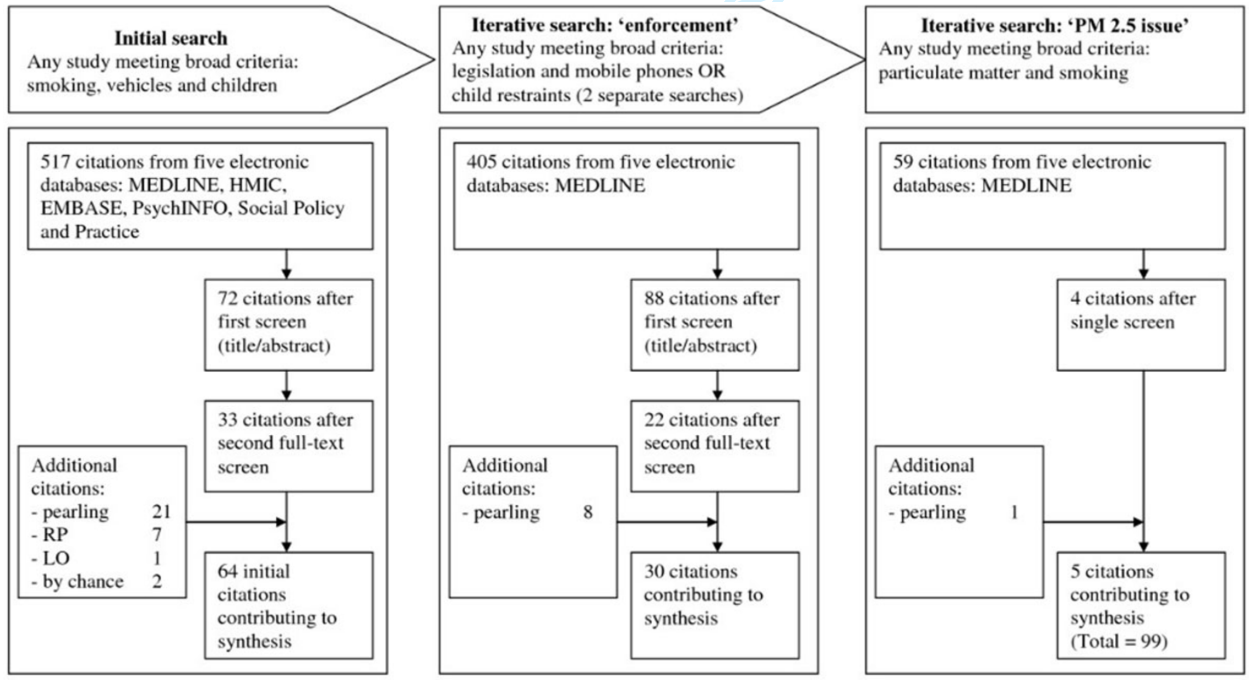

Figure 1 Flow diagram illustrating search process and article disposition [26].

For peer review

For peer review only - http://bmjopen.bmj.com/site/about/guidelines.xhtml

poverty neighborhood/community; high-poverty neighborhood/community; neighborhood/community health; poverty neighborhood/community; poverty community effects; poverty housing; poverty health; and housing health. A "snowball" approach was used in which one reference led to others. Other evaluations were revealed through correspondence with Dr. Jeffrey Kling, one of the principal MTO researchers." [28]

Explanation

Searching should be guided by the objectives and focus of the synthesis, and revised iteratively in the light of emerging data. Data relevant to a realist synthesis may lie in a broad range of sources that may cross traditional disciplinary, program and sector boundaries. The search phase is thus likely to involve searching for different sorts of data, or studies from different domains, with which to test different aspects of any provisional theory.

Search methods using forward and backward citation tracking may be particularly valuable in finding the documents necessary to develop and then test provisional theories. Realist syntheses do not exclude sources solely on the basis of their study design; hence, 'methodological filters' (for example, to identify randomized controlled trials) may add little to the search and could potentially miss relevant papers.

Searching is likely to be iterative because, as the synthesis progresses, new or refined elements of theory may be required to explain particular findings, or to examine specific aspects of particular processes. As new elements of theory are included, searches for evidence to support, refute or refine those elements may be required. If undertaken, the process used for any such additional searches should be clearly documented. A single pre-defined search is unlikely to be sufficient and may suggest insufficient reflection on emerging findings.

Sufficient detail should be given to enable the reader to judge whether searching was likely to have located sources needed for theory building and/or testing.

Item 9: Selection and appraisal of documents

Explain how judgements were made about including and excluding data from documents, and justify these.

Example

"Three tools were developed (for identification, selection, and appraisal) in March, June, and October 2009, respectively. Modifications were made during each stage after piloting. Each stage processed a different type of data: citations in identification; full-text papers in selection; and sets of publications in appraisal.

...The identification tool consisted of three questions. This step funnelled the number of citations from 7,167 to 594.

The librarian (JH) retrieved the 594 full-text papers, which were read by two independent reviewers, using a selection tool initially comprised of six questions in June 2009, with an additional two questions added in October 2009. ...

Two hundred articles remained from 594 after filtering them through the selection tool. Due to the complexity of the dataset, we decided at this stage to further limit the scope of our review to community-based settings, and to participatory interventions. Our rationale was that: PR in all forms (community-based PR, organizational PR, action research) was too diverse to be assessed within one review; the complexity of PR benefits from community-based research provided a manageable set of studies; intervention research demonstrated more complexity of outcomes than non-intervention research, and would be best suited for analysis using realist review methods; and the pool of studies needed to be reduced to a manageable size for an in depth realist synthesis (analysis). Adding two questions reduced the pool to 83 studies.....

Contact with principal investigators of all full-text papers retained after selection was undertaken because descriptions of programs, methods and findings of PR interventions were found to be commonly described across a number of publications pertaining to the same intervention. It was thus necessary to confirm that we had complete sets of papers in order to fairly appraise projects according to the realist review approach. ... For each study, we then sent our list of papers to the corresponding author or PI, and asked them to confirm that we had the complete set, or to send us additional documents. ... Only those sets of studies in which the contacted researcher responded to our request were retained for appraisal.

.... The appraisal tool consisted of three questions. An additional 11 sets were eliminated after screening with the tool below, which left a total of 23 sets, comprising 276 documents that were retained for synthesis. See Appendix 4: ... for a complete breakdown of the number of cases retained at each stage." [30]

Explanation

Realist synthesis is not a technical process - that is, following a set protocol will not guarantee that a review will be robust. Rather, it requires a series of judgements about the relevance and robustness of particular data for the purposes of answering a specific question.

Within any document, there may be several pieces of data that serve different purposes, such as helping to build one theory, refining another theory and so on. Therefore, the selection (for inclusion or exclusion) and appraisal of the contribution of pieces of data within a document cannot be based on an overall assessment of study or

For peer review only - http://bmjopen.bmj.com/site/about/guidelines.xhtml

document quality. An appraisal of the contribution of any section of data (within a document) should be made on two criteria:

- *Relevance* - whether it can contribute to theory building and/or testing; and
- *Rigor* - whether the method used to generate that particular piece of data is credible and trustworthy.

A wide range of documents can potentially contribute to a realist synthesis. For example, outcome and impact studies, qualitative interviews, ethnography, questionnaire surveys, mixed-method case studies, and close reading of policies, business plans, websites, project initiation documents and 'gray literature' write-ups of programs may all contribute in different ways of identifying and elucidating program theories. Because of this range and realist review's focus on relevance and rigor, it can initially be difficult to 'whittle down' the number of documents that are potentially eligible for inclusion in a review. This process can only occur as the data sources are analyzed in detail. Thus, in practice, the selection and appraisal stage may need to run in parallel with the analysis stage.

It is unlikely that authors will be able to provide an in-depth description of each decision involved, but the broad processes used to determine relevance and assess rigor (for example, using quality standards appropriate to particular kinds of research to appraise documents or sections of documents; discussion and/or debate within a review team of a document's findings; or consulting experts about technical aspects of methods or findings) should be described. While the description of the processes followed will not allow the reader to draw firm conclusions about judgements made, it will give an indication of the coherence, plausibility and appropriateness of the processes used to inform those judgements.

Item 10: Data extraction

Describe and explain which data or information were extracted from the included documents and justify this selection.

Example

"In order to identify key elements of importance to the success or failure of an intervention in a certain context using a realist perspective, information was gathered on the intervention, the context and the actual "working of the intervention" or the mechanisms. As we intended to discuss the strength of the evidence and the usefulness of the application of realist principles to already published studies, we developed a process of data analysis that was comprehensive and as objective and transparent as possible. Therefore, a data analysis matrix was developed by the team of authors (see Annex 2). During the development of this matrix, the team extensively discussed and defined terms (such as context, mechanisms and outcome) and evaluation levels (such as process, output and outcome)." [31]

Explanation

In a realist synthesis, data extraction assists analysis and synthesis. Reporting on what was extracted and why can add to the transparency of the synthesis process.

The extracted data may consist of descriptions (for example, of the detail of what was done in a program), findings (for example, cure rates, mortality) or explanations about how and why the program may have worked in particular contexts. Of particular interest to the realist reviewer are data that support the use of realist logic to answer the review's question(s) - for example, data on context, mechanisms and outcome configurations, demi-regularities, middle-range and/or program theories. Realist synthesis is used for a wide range of research questions, so it is impossible to be prescriptive about what data should be extracted. However, the link between the research question and the category of data extracted should be clear.

Item 11: Analysis and synthesis processes

Describe the analysis and synthesis processes in detail. This section should include information on the constructs analyzed and describe the analytic process.

Example

"Data synthesis was undertaken either by RP and/or GW and synthesis results were regularly shared and discussed within the review team to ensure validity and consistency in the inferences made. Specifically (where relevant), we attempted to identify prominent recurrent patterns of contexts and outcomes (demi-regularities) in the data and then sought to explain these through the means (mechanisms) by which they occurred. For example, we noted that in our included articles self-reported public support for a ban on smoking in vehicles carrying children was often found to be high amongst smokers. During data synthesis we would then aim to provide an explanation of this demi-regularity through the identification of mechanism(s). As we delved further into our included articles and beyond (through our aforementioned purposive searching) for an explanation, data emerged that smokers harboured within them the wish to want to protect children from harm and also regret at having started smoking. We interpreted these as (realist) mechanisms and, for the former, were able to find substantive (middle-range) theory in the form of the Millean principle [Reference x1] to explain its interaction with context to influence outcomes. When additional studies were sought to enable programme theory testing, data handling processes .... were repeated." [26]

For peer review only - http://bmjopen.bmj.com/site/about/guidelines.xhtml

Explanation

In a realist synthesis, the analysis and synthesis processes occur iteratively and may be sequential or in parallel. At the center of any realist analysis is the application of a realist philosophical 'lens' to data. A realist analysis of data specifically seeks to analyze data using realist concepts. Specifically, realism adheres to a generative explanation for causation - that is, an outcome (O) of interest was generated by relevant mechanism(s) (M) being triggered in context (C). Within or across the included documents, recurrent patterns of outcomes (or demi-regularities) and their associated mechanisms and contexts (CMO configurations) are likely to occur.

During synthesis the goal is to make sense of the analyzed data using theory, at one of two levels. First, theory (or theories) may be sought, developed and/or refined to explain how it is that a program (or part of a program) achieves its outcomes (that is, the mechanism(s) operating within a program) and the contexts in which those mechanisms do and do not fire. This provides a realist program theory. Second, theory (or theories) may be sought, developed and/or refined to explain, at a somewhat more general level, the pattern of contexts, mechanisms and outcomes. A full realist analysis addresses both these levels and attempts to make sense of the relationship between these two levels. Syntheses which address only one level may also be considered realist syntheses assuming that they apply and demonstrate application of a realist philosophy of science. The level(s) of analysis chosen will depend on the review's focus. The theories used may have been developed and/or refined from the data and/or be refinement of existing substantive theory.

The key analytic process in realist review involves iterative testing and refinement of theoretically based explanations using empirical findings in data sources. Reviewers may draw on any appropriate analytic techniques to undertake this testing. Explanation and justification for the choice of techniques should be provided.

Ideally a description should be provided on how all the individuals involved in the review have been involved in the analysis and synthesis processes, and how these evolved as the review took shape.

Results section

The following items should be reported in the results section.

Item 12: Document flow diagram

Provide details on the number of documents assessed for eligibility and included in the review with reasons for exclusion at each stage, as well as an indication of their source of origin (for example, from searching databases, reference lists and so on). You may consider using the example provided (which is likely to need modification to suit the data) in Figure 1.

Example

"See Figure 1: Flow diagram illustrating search process and article disposition." [26]

Explanation

A flow diagram provides an accessible summary of the sequence of steps and gives an indication of the volume of data included and excluded at each step.

Item 13: Document characteristics

Provide information on the characteristics of the documents included in the synthesis.

Example

"Additional File 1 summarises ..., the context, the intervention, the mechanisms triggered and the reported outcomes. Additional File 1 shows that in all the trials, more than one type of intervention was applied to improve CHWs [community health workers] performance. It also shows that the outcomes are reported not in terms of CHW performance, but rather in terms of the consequences of their performance on specific health outcomes." [27]

Explanation

A clear summary of the characteristics of included sources can add to the transparency of the synthesis and some characteristics may help readers judge the coherence and plausibility of inferences. Examples of possibly relevant characteristics of documents that may be worth reporting include, where applicable: full citation, country of origin, study design, summary of key main findings, use made of document in the synthesis and relationship of documents to each other (for example, there may be more than one document reporting on an intervention). While considering specific requirements of any particular publication, reviewers may wish to tabulate key characteristics.

Item 14: Main findings

Present the key findings with a specific focus on theory building and testing.

Example

"Using this theoretical concept, we hypothesized that equitable partnerships, with the stakeholders' participation throughout the project, succeed largely through synergy. Through the synthesis process using CMO configuring, we refined the theory by demonstrating that synergy is both an outcome and a context for partnership development - so that when synergy generated positive outcomes (e.g., enhanced trust or improved data collection), those outcomes generated new synergy. Expanding this logic, we demonstrated how partnership synergy created momentum over time, producing resilience in the face of obstacles as well as sustaining health-related goals, extending

For peer review only

programs and infrastructure, and creating new and unexpected ideas and activities." [30]

Explanation

The defining feature of a realist synthesis is the nature of the theory(ies) it offers. Such a theory explains why a social program/intervention generates particular outcomes in particular contexts, in terms of one or more mechanisms - that is how the program's infrastructure and resources trigger particular decisions or behaviors in human participants. Program theories are usually 'middle-range' - that is, specific enough to generate propositions that can be tested about aspects of the program but sufficiently abstract to be applicable to other programs. Mechanisms are contingent: they are causal processes that have a tendency to occur in a particular set of conditions, but which do not always occur (because the circumstances have to be right for any particular mechanism to operate, and because many mechanisms can operate concurrently, sometimes cancelling each other out).

The validity of a review which is described as 'realist' and which talks about program theories or mechanisms but which expresses these as simple and linear relationships between variables should be questioned.

The findings of a realist synthesis consist largely of inferences about the links between context, mechanism and outcome and the theory(ies) that seek to account for these links. It is important that where inferences are made these are clearly articulated. Where possible, especially for key findings, it is important to include an explanation to show how these inferences were arrived at.

Transparency of the synthesis process can be demonstrated, for example, by including such things as a detailed worked example, verbatim quotes from primary sources, and (if appropriate) an exploration of disconfirming data (that is, findings which appeared to refute the program theory but which, on closer analysis, could be explained by other contextual influences).

When presenting inferences about context-mechanism-outcome configurations, reviewers should be clear about what they have categorized as context, what as mechanism and what as outcome. In a realist synthesis a mechanism involves the interaction between particular inputs (or resources) and human reasoning, which produces a particular outcome (or not).

More than one piece of data might be needed to support an inference. It is sometimes appropriate to build the argument for an inference as an unfolding narrative in which successive data sources increase the strength of the inference [32]. Provide enough details about each data item to identify its source and enable readers to make judgements about its relevance and rigor.

Discussion section

The following Items should be reported in the discussion section.

Item 15: Summary of findings

Summarize the main findings, taking into account the synthesis' objective(s), research question(s), focus and intended audience(s).

Example

"This realist review of 249 primary studies has produced two key findings which are important, if somewhat unsurprising. First, Internet-based courses must engage their target group of learners to use the technology. This is likely to occur only if the technology is perceived as 'useful' (e.g.increases access to learning or saves time) and 'easy to use', though benefits in the former can outweigh challenges in the latter. Second, 'interactivity' is highly valued by learners. Learners wanted to be able to enter into a dialogue with the course tutor, fellow students and/or a virtual tutorial and obtain ongoing feedback on their understanding and performance." [29]

Explanation

In order to place the findings in the context of the wider literature and any specific policy need, it is necessary to summarize briefly what has been found. This section should be succinct and balanced, explaining the relevance of one or more key theories that emerged from the analysis and highlighting the strength of evidence for the main inferences. This should be done with careful attention to the needs of the main users of the synthesis.

Item 16: Strengths, limitations and future research directions

Discuss both the strengths of the review and its limitations. These should include (but need not be restricted to) (a) consideration of all the steps in the synthesis process and (b) comment on the overall strength of evidence supporting the explanatory insights that emerged.

The limitations identified may point to areas where further work is needed.

Example

"We explicitly chose to do a realist review of the RCTs [randomized controlled trials] to see what they could additionally yield. While the CHWs [community health workers] were an important component of the interventions being tested in the RCTs, none of the RCTs under review explicitly focused on performance of the CHW as an outcome. The RCTs under review offered a fair amount of information about the interventions, only some information about context - allowing us to formulate only generic hypotheses. ...

... Authors seldom described or discussed the mechanisms that explained their study outcomes. We realise that

For peer review only

the RCT design, the exacting reporting requirements and word limits of journals, restrict authors from sharing all their operational experiences. In addition RCTs tend to report average effects and not differential effects of interventions, and less so of the context and rarely of the mechanisms triggered by their interactions. This makes the RCTs less useful for answering the questions regarding how interventions work. These generic hypotheses seem to be recurring in the literature, however they have not been explicitly tested across contexts." [27]

Explanation

Realist synthesis may be constrained by time and resources, by the skill mix and collective experience of the research team, by the scope of the review's questions or objectives and/or by anticipated or unanticipated challenges in the data. These should be made explicit so that readers can interpret the findings in the light of them. A common challenge in realist synthesis is that in order to focus the synthesis, some material is omitted at each successive stage. Some aspects of the topic area, therefore, end up being reviewed in detail and rich explanatory insights produced for these. Other aspects are neglected (relatively or absolutely). It is thus inevitable that in generating illumination, the synthesis will also cast shadows. These should be highlighted in the discussion so as to indicate areas where other syntheses might focus.

Strengths and/or limitations associated with any modifications made to the synthesis process should also be reported and justified.

Item 17: Comparison with existing literature

Where applicable, compare and contrast the synthesis' findings with the existing literature (for example, other reviews) on the same topic.

Example

"We were unable to find any comparable attempt at providing an evidence-based-policy framework such as ours. However, we acknowledge that some sections of our framework may be found in sources we have not uncovered and also as tacit knowledge within the heads of seasoned practitioners (e.g. advocates or legislators). We do however hope that our attempts to develop and test it on our one 'case study' will make a primordial tool that will be useful to policy and decisions makers less well versed in the arena of public health legislation." [26]

Explanation

Comparing and contrasting the findings from a synthesis with the existing literature may help readers to put these into context. For example, this item might cover questions such as: How does this synthesis compare to other reviews (for example, were they theory-driven?); What does this synthesis add?; Which body of work in particular does it add to?; Has this synthesis reached the same or different conclusion to previous reviews?; and Has it answered a question previously identified as important in the field?

Item 18: Conclusion and recommendations

List the main implications of the findings and place these in the context of other relevant literature. If appropriate, offer recommendations for policy and practice.

Example

"Our realist review was based on a housing intervention in the United States, but the results can potentially be applied to urban centers in other nations that implement housing interventions that involve moving families. When a family moves, the experience is likely to be different for each member of the household, and differences in mental health outcomes of moving may occur (Reference x1). All communities, rich or poor, and irrespective of geographic location, should be viewed as complex systems, and as composed of people with social relationships that influence the functioning and health of community members." [28]

Explanation

A clear line of reasoning is needed to link the findings (Results section) with the implications (Discussion and/or Conclusion). If the synthesis is small and preliminary, or if the coherence and plausibility of evidence behind the inferences is weak or moderate, statements about implications for practice and policy should be appropriately guarded.

If recommendations are given, these should take into account the focus of the synthesis and needs of the intended audience and be presented appropriately. The explanations in realist analysis are highly dependent on contextual influences. It follows that recommendations must be contingent (for example, only under certain contexts will a particular mechanism be triggered to generate the desired outcome) rather than statements that X should or should not be done.

Item 19: Funding

Provide details of funding source (if any) for the synthesis, the role played by the funder (if any) and any conflicts of interests of the reviewers.

Example

"We gratefully acknowledge a financial contribution from the Dutch Development Cooperation (DGIS)." [25]

Explanation

The source of funding for a synthesis and/or personal conflicts of interests may influence the research question, methods, data analysis and conclusions. No review is a 'view from nowhere', and readers will be better able to interpret the review if they know why it was done and for which sponsor.

For peer review only

Wong et al. BMC Medicine 2013, 11:21
http://www.biomedcentral.com/1741-7015/11/21

If a synthesis is published, the process for reporting funding and conflicts of interest as set out by the publication concerned should be followed.

Discussion

We have developed these publication standards for realist synthesis (which we view as synonymous with realist review) by drawing together a range of sources - namely, existing published evidence, a Delphi panel and comment, discussion and feedback from a mailing list, training sessions and workshops. We hope these standards will lead to greater consistency and rigor of reporting and, thereby, make the outputs of realist synthesis more accessible, usable and helpful to different stakeholders.

This publication standard is not a detailed guide of how to undertake a realist synthesis. Other resources, both published (see Background) and in preparation, are better suited for this purpose. These standards have been developed as a guide to assist the quality of reporting of realist syntheses and the work of publishers, editors and reviewers. As part of the RAMESES project, we will be developing and disseminating both training materials and quality standards for realist synthesis [20].

Because realist synthesis is used for a broad range of topics and questions, and because it involves making judgements and inferences rather then checking against or following a technical checklist, it is impossible to be prescriptive about what exactly must be done in a review. The guiding principle is that transparency is important, as this will help readers to decide for themselves if the arguments for the judgements made were reasonable, both for the chosen topic and from a methodological perspective. We strongly encourage review authors to provide detail on what they have done and how - in particular with respect to the analytic processes used. These standards are intended to supplement rather than replace the exercise of judgement by editors, reviewers, readers and users of realist syntheses. We have tried to indicate in each item where judgement needs to be exercised.

The explanatory and theory-driven focus of realist syntheses means that detailed data may need to be reported in order to provide enough support for inferences and/or judgments made. While developing these publication standards, it became apparent that in some cases the word count limitations imposed by journals did not enable review teams to fully explain aspects of their synthesis - such as how judgments were made or inferences arrived at. Alternative ways of providing the necessary detail may need to be found, such as online appendices or additional files available from authors on request.

Previous efforts to develop publication standards have sometimes been criticized for being too 'ivory-tower' and failing to take account of real-world problems faced by reviewers. In an effort to redress this problem in the RAMESES project, we sought from the outset to engage not just senior academics but also junior and mid-career researchers, practitioners, policymakers and publishers in the development of the standards and to capture real-life challenges of ongoing realist syntheses as these emerged.

Conclusions

We have developed these publication standards for realist syntheses by drawing on a range of sources. Our hope is that these standards will lead to greater consistency and rigor of reporting and make the outputs of realist syntheses more accessible, usable and helpful to different stakeholders. Realist synthesis is a relatively new approach to evidence synthesis and with increasing use and methodological development, changes are likely to be needed to any publication standards. We hope to continue capturing and improving these publication standards, through our email list [21] and wider links and discussions with researchers and those who commission, sponsor, publish and use realist syntheses.

Abbreviations

ESRC: Economic and Social Research Council; RAMESES: Realist And MEta-narrative Evidence Syntheses: Evolving Standards).

Acknowledgements

This project was funded by the National Institute for Health Research Health Services and Delivery Research Programme (NIHR HS&DR) - project number 10/1008/07.

We thank the following individuals for their participation in the RAMESES Group and contributions to the Delphi panel:

Dave Baker, Sinai Hospital of Baltimore (Baltimore, USA); Marcello Bertotti, University of East London (London, UK); Allan Best, InSource (Vancouver, Canada); Margaret Cargo, University of South Australia (Adelaide, Australia); Simon Carroll, University of Victoria (Victoria, Canada); Colleen Davison, Queens University, (Kingston, Canada); Marjolein Dieleman, Royal Tropical Institute (Amsterdam, Netherlands); Tim Dornan, Maastricht University (Maastricht, Netherlands); Ruth Garside, Peninsula College of Medicine and Dentistry (Exeter, UK); Bradford Gray, Milbank Quarterly (New York, USA); Joanne Greenhalgh, University of Leeds (Leeds, UK); Lois Jackson, Dalhousie University (Halifax, Canada); Justin Jagosh, McGill University (Montreal, Canada); Monika Kastner, University of Toronto (Toronto, Canada); James Lamerton, Sunshine Coast Division of General Practice (Cotton Tree, Australia); Fraser MacFarlane, Queen Mary, University of London (London, UK); Bruno Marchal, Institute of Tropical Medicine (Antwerp, Belgium); Tracey McConnell, Queen's University (Belfast, UK); Gemma Moss, Institute of Education (London, UK); Douglas Noble, Queen Mary, University of London (London, UK); Patricia O'Campo, University of Toronto (Toronto, Canada); Mark Pearson, Peninsula College of Medicine and Dentistry (Exeter, UK); Pierre Pluye McGill University (Montreal, Canada); Henry Potts, University College London (London, UK); Barbara Riley, University of Waterloo, (Waterloo, Canada); Glenn Robert, Kings College London (London, UK); Jessie Saul, North American Research & Analysis, Inc (Faribault, USA); Paul Shekelle, RAND Corporation (Santa Monica, USA); Neale Smith, University of British Columbia (Vancouver, Canada); Sanjeev Sridharan, University of Toronto (Toronto, Canada); Deborah Swinglehurst, Queen Mary, University of London (London, UK); Nick Tilley, University College London (London, UK); Kieran Walshe, University of Manchester (Manchester, UK).

All the authors (except JB) were also members of the Delphi panel.

Author details

[1]Centre for Primary Care and Public Health, Queen Mary University of London, 58 Turner Street, London E1 2AB, UK. [2]Community Matters, P.O. Box 443, Mount Torrens, SA 5244, Australia. [3]John W. Scott Health Sciences Library, University of

For peer review only - http://bmjopen.bmj.com/site/about/guidelines.xhtml

Alberta, Edmonton, AB T6G 2R7, Canada. [4]Department of Social Research Methodology, University of Leeds, Leeds LS2 9JT, UK.

Authors' contributions

GWo carried out the literature review. JB searched the literature for realist syntheses. GWo, TG, GWe and RP analyzed the findings from the review and produced the materials for the Delphi panel. They also analyzed the results of the Delphi panel. GWo, TG, GWe and RP conceived of the study and participated in its design. GWo coordinated the study and ran the Delphi panel. All authors read and approved the final manuscript.

Competing interests

The authors declare that they have no competing interests. The views and opinions expressed therein are those of the authors and do not necessarily reflect those of the HS&DR program, NIHR, NHS or the Department of Health.

Received: 27 September 2013 Accepted: 29 January 2013
Published: 29 January 2013

References

1. Pawson R: *Evidence-based Policy: A Realist Perspective* London: Sage; 2006.
2. Pearson M, Coomber R: **The challenge of external validity in policy-relevant systematic reviews: a case study from the field of substance misuse.** *Addiction* 2010, **105**:136-145.
3. Dixon-Woods M, Agarwal S, Jones D, Young B, Sutton A: **Synthesising qualitative and quantitative evidence: a review of possible methods.** *J Health Serv Res Policy* 2005, **10**:45-53.
4. Barnett-Page E, Thomas J: **Methods for the synthesis of qualitative research: a critical review.** *BMC Med Res Methodol* 2009, **9**:59.
5. Pope P, Mays N, Popay J: *Synthesizing Qualitative and Quantitative Health Research: A Guide to Methods* Maidenhead, UK: Open University Press; 2007.
6. Gough D, Oliver S, Thomas J: *An Introduction to Systematic Reviews* London: Sage; 2012.
7. Kastner M, Tricco A, Soobiah C, Lillie E, Perrier L, Horsley T, Welch V, Cogo E, Antony J, Straus S: **What is the most appropriate knowledge synthesis method to conduct a review? Protocol for a scoping review.** *BMC Med Res Methodol* 2012, **12**:114.
8. Merton R: *On Theoretical Sociology. Five Essays, Old and New* New York: The Free Press; 1967.
9. Astbury B, Leeuw FL: **Unpacking black boxes: mechanisms and theory building in evaluation.** *Am J Eval* 2010, **31**:363-381.
10. Shepperd S, Lewin L, Straus S, Clarke M, Eccles M, Fitzpatrick R, Wong G, Sheikh A: **Can we systematically review studies that evaluate complex interventions?** *PLoS Med* 2009, **6**:e1000086.
11. Rycroft-Malone J, McCormack B, Hutchinson AM, DeCorby K, Bucknall TK, Kent B, Schultz A, Snelgrove-Clarke E, Stetler CB, Titler M, Wallin L, Wilson V: **Realist synthesis: illustrating the method for implementation research.** *Implement Sci* 2012, **7**:33.
12. Robert E, Ridde V, Marchal B, Fournier P: **Protocol: a realist review of user fee exemption policies for health services in Africa.** *BMJ Open* 2012, **2**: e000706.
13. Pawson R: **Evidence-based policy: the promise of 'realist synthesis'.** *Evaluation* 2002, **8**:340-358.
14. Pawson R, Greenhalgh T, Harvey G, Walshe K: *Realist Synthesis - an Introduction. ESRC Working Paper Series* London: Economic and Social Research Council; 2004.
15. Pawson R, Greenhalgh T, Harvey G, Walshe K: **Realist review–a new method of systematic review designed for complex policy interventions.** *J Health Serv Res Policy* 2005, **10(Suppl 1)**:21-34.
16. Moher D, Hopewell S, Schulz K, Montori V, Gotzsche P, Devereaux P, Elbourne D, Egger M, Altman D, for the CONSORT Group: **CONSORT 2010 explanation and elaboration: updated guidelines for reporting parallel group randomised trial.** *BMJ* 2010, **340**:c869.
17. Brouwers M, Kho M, Browman G, Burgers J, Cluzeau F, Feder G, Fervers B, Graham I, Grimshaw J, Hanna S, Littlejohns P, Makarski J, Zitzelsberger L, AGREE Next Steps Consortium: **AGREE II: advancing guideline development, reporting and evaluation in healthcare.** *Can Med Assoc J* 2010, **182**:E839-842.
18. Liberati A, Altman DG, Tetzlaff J, Mulrow C, Gøtzsche PC, Ioannidis JP, Clarke M, Devereaux PJ, Kleijnen J, Moher D: **The PRISMA statement for reporting systematic reviews and meta-analyses of studies that evaluate healthcare interventions: explanation and elaboration.** *BMJ* 2009, **339**: b2700.
19. Davidoff F, Batalden P, Stevens D, Ogrinc G, Mooney S, SQUIRE Development Group: **Publication guidelines for improvement studies in health care: evolution of the SQUIRE project.** *Ann Intern Med* 2008, **149**:670-676.
20. Greenhalgh T, Wong G, Westhorp G, Pawson R: **Protocol - realist and meta-narrative evidence synthesis: evolving standards (RAMESES).** *BMC Med Res Methodol* 2011, **11**:115.
21. RAMESES JISCM@il: [www.jiscmail.ac.uk/RAMESES].
22. Wong G, Greenhalgh T, Westhorp G, Pawson R: **Realist methods in medical education research: what are they and what can they contribute?** *Med Educ* 2012, **46**:89-96.
23. Wong G: **The internet in medical education: a worked example of a realist review.** In *Synthesising Qualitative Research: Choosing the Right Approach.* Edited by: Hannes K, Lockwood C. Chicester, UK: Wiley-Blackwell; 2011:.
24. EQUATOR Network: [http://www.equator-network.org/home/].
25. Dieleman M, Gerretsen B, van der Wilt GJ: **Human resource management interventions to improve health workers' performance in low and middle income countries: a realist review.** *Health Res Policy Syst* 2009, **7**:7.
26. Wong G, Pawson R, Owen L: **Policy guidance on threats to legislative interventions in public health: a realist synthesis.** *BMC Public Health* 2011, **11**:222.
27. Kane SS, Gerretsen B, Scherpbier R, Dal Poz M, Dieleman M: **A realist synthesis of randomised control trials involving use of community health workers for delivering child health interventions in low and middle income countries.** *BMC Health Serv Res* 2010, **10**:286.
28. Jackson L, Langille L, Lyons R, Hughes J, Martin D, Winstanley V: **Does moving from a high-poverty to lower-poverty neighborhood improve mental health? A realist review of 'Moving to Opportunity'.** *Health Place* 2009, **15**:961-970.
29. Wong G, Greenhalgh T, Pawson R: **Internet-based medical education: a realist review of what works, for whom and in what circumstances.** *BMC Med Educ* 2010, **10**:12.
30. Jagosh J, Macaulay AC, Pluye P, Salsberg J, Bush PL, Henderson J, Sirett E, Wong G, Cargo M, Herbert CP, Seifer SD, Green LW, Greenhalgh T: **Uncovering the benefits of participatory research: implications of a realist review for health research and practice.** *Milbank Q* 2012, **90**:311-346.
31. Dieleman M, Kane S, Zwanikken P, Gerretsen B: *Realist Review and Synthesis of Retention Studies for Health Workers in Rural and Remote Areas* Geneva, Switzerland: World Health Organization; 2011.
32. Pawson R, Wong G, Owen L: **Known knowns, known unknowns, unknown unknowns: the predicament of evidence-based policy.** *Am J Eval* 2011.

doi:10.1186/1741-7015-11-21
Cite this article as: Wong *et al.*: RAMESES publication standards: realist syntheses. *BMC Medicine* 2013 **11**:21.

Submit your next manuscript to BioMed Central and take full advantage of:

- **Convenient online submission**
- **Thorough peer review**
- **No space constraints or color figure charges**
- **Immediate publication on acceptance**
- **Inclusion in PubMed, CAS, Scopus and Google Scholar**
- **Research which is freely available for redistribution**

Submit your manuscript at
www.biomedcentral.com/submit
BioMed Central

For peer review only

BMJ Open

Integrating Palliative Care and Heart Failure: a protocol for a realist synthesis (PalliatHeartSynthesis)

Journal:	*BMJ Open*
Manuscript ID	bmjopen-2021-058848.R1
Article Type:	Protocol
Date Submitted by the Author:	09-Nov-2021
Complete List of Authors:	McConnell, Tracey ; Queen's University Belfast School of Nursing and Midwifery; Marie Curie Hospice Belfast<br>Burden, John; British Heart Foundation, Patient and Public Involvement Group<br>Duddy, Claire; University of Oxford, Nuffield Department of Primary Care Health Sciences<br>Hill, Loreena; Queen's University Belfast, School of Nursing and Midwifery<br>Howie, Clare; Queen's University Belfast, School of Nursing and Midwifery<br>Jones, Bob; British Heart Foundation, Patient and Public Involvement Group<br>Ruane, Bob; British Heart Foundation, Patient and Public Involvement Group<br>Wong, Geoff; Oxford University, Nuffield Department of Primary Care Health Sciences<br>Reid, Joanne; Queen's University Belfast, School of Nursing and Midwifery
**Primary Subject Heading**:	Palliative care
Secondary Subject Heading:	Cardiovascular medicine, Health services research
Keywords:	Heart failure < CARDIOLOGY, PALLIATIVE CARE, Organisation of health services < HEALTH SERVICES ADMINISTRATION & MANAGEMENT

SCHOLARONE™ Manuscripts

I, the Submitting Author has the right to grant and does grant on behalf of all authors of the Work (as defined in the below author licence), an exclusive licence and/or a non-exclusive licence for contributions from authors who are: i) UK Crown employees; ii) where BMJ has agreed a CC-BY licence shall apply, and/or iii) in accordance with the terms applicable for US Federal Government officers or employees acting as part of their official duties; on a worldwide, perpetual, irrevocable, royalty-free basis to BMJ Publishing Group Ltd ("BMJ") its licensees and where the relevant Journal is co-owned by BMJ to the co-owners of the Journal, to publish the Work in this journal and any other BMJ products and to exploit all rights, as set out in our licence.

The Submitting Author accepts and understands that any supply made under these terms is made by BMJ to the Submitting Author unless you are acting as an employee on behalf of your employer or a postgraduate student of an affiliated institution which is paying any applicable article publishing charge ("APC") for Open Access articles. Where the Submitting Author wishes to make the Work available on an Open Access basis (and intends to pay the relevant APC), the terms of reuse of such Open Access shall be governed by a Creative Commons licence – details of these licences and which Creative Commons licence will apply to this Work are set out in our licence referred to above.

Other than as permitted in any relevant BMJ Author's Self Archiving Policies, I confirm this Work has not been accepted for publication elsewhere, is not being considered for publication elsewhere and does not duplicate material already published. I confirm all authors consent to publication of this Work and authorise the granting of this licence.

For peer review only

Integrating Palliative Care and Heart Failure: a protocol for a realist synthesis (PalliatHeartSynthesis)

Tracey McConnell[1,2], John Burden[3], Claire Duddy[4], Loreena Hill[1], Clare Howie[1], Bob Jones[3], Bob Ruane[3], Geoff Wong[4] & Joanne Reid[1].

[1]School of Nursing and Midwifery, Queen's University Belfast, UK

[2] Marie Curie Marie Curie Hospice, Belfast, Kensington Road, Belfast BT5 6NF

[3]British Heart Foundation London Heart Failure Group Member, UK

[4]Nuffield Department of Primary Care Health Sciences, Radcliffe Observatory Quarter, Oxford, UK

Corresponding author: Tracey McConnell, School of Nursing and Midwifery, Queen's University Belfast, UK, Marie Curie Marie Curie Hospice, Belfast, Kensington Road, Belfast BT5 6NF, t.mcconnell@qub.ac.uk, 07549437132

Abstract

Introduction

Heart failure affects over 26 million people worldwide with prevalence expected to grow due to an ageing global population. Palliative care can address the holistic needs of patients with heart failure, and integrated palliative care in heart failure management has been indicated to improve outcomes for patients. Despite known benefits for integrated palliative care in heart failure management, implementation is poor across the majority of global health services. Recent systematic reviews have identified the benefits of integrating palliative care into heart failure management and highlighted barriers to implementation. However, there was heterogeneity in terms of countries, healthcare settings, delivery by differing staff across multidisciplinary teams, modes of delivery and different intervention components.

Methods & Analysis

The aim of this study is to identify how integrated palliative care and heart failure interventions produce desired outcomes, in which contexts, and for which patients. We will undertake a realist synthesis to identify this, using Pawson's five iterative steps. We will

For peer review only

recruit an international stakeholder group comprised of healthcare providers and patients with heart failure to advise and provide feedback throughout the review. Our initial realist programme theory sets out the necessary steps needed to accomplish the final intended outcome(s) from the implementation of integrated palliative care and heart failure. This initial programme theory will be shaped through an iterative process of testing and refinement.

Ethics and dissemination.

Ethical approval is not required for this study. With our stakeholder group, we will co-produce a user guide that outlines practical advice to optimise, tailor and implement interventions designed to integrate palliative care and heart failure, taking into consideration local context, alongside user-friendly summaries of the synthesis findings using short animations to convey complex findings. We will draw on the expertise within the stakeholder group to identify key stakeholders for disseminating to relevant audiences, ensuring outputs are tailored for their respective needs.

Prospero Registration number CRD42021240185.

For peer review only

Article Summary: Strengths and limitations of this study

- This is the first realist synthesis examining the integration of palliative care into routine heart failure management.
- This synthesis will be supported by a large multidisciplinary, international stakeholder group of policy, practice and patient experts to maximise the potential for widespread implementation, reduce healthcare costs, and improve outcomes for patients and informal carers
- Potential limitations include lack of relevance and rigour in the available literature

Keywords: Heart failure, palliative care, organisation of health services

Word count: 3924 words

Figures:2

Introduction

Background

Cardiovascular disease is the most common cause of death worldwide [1]. Heart failure characterises the final phenotype of many cardiovascular diseases, estimated to currently affect at least 26 million people worldwide [2], with this number expected to grow due to ageing populations [3]. Patients with advanced heart failure or those classified as experiencing New York Heart Association (NYHA) Class III or Class IV symptoms, account for over one million hospitalisations per year in the United States and Europe [4], with an overall estimated economic cost of $108 billion per annum [5]. Integrating palliative care with routine management of heart failure has been shown to significantly reduce healthcare costs overall compared to usual care [6] and significantly reduces the number of hospital visits and duration of in-patient stays [7,8]. The National Audit Office review of end of life care recommended palliative care for patients with heart failure, due to a possible cost savings by reducing utilisation of acute services [9].

There is evidence of improved patient and family outcomes when palliative care is integrated in advanced heart failure management. A review of carers' needs identified that integrated palliative care in heart failure management led to an improvement in satisfaction with care from both the patient and their informal carer [10]. Informal carers are typically defined as those who provide unpaid care to individuals with whom they have a relationship, i.e. family members or spouses [11]. Informal carers are crucial to facilitating independent living and supporting quality of life for patients with heart failure and palliative care can address caregivers' needs and help them care for their loved one [12]. Integrated palliative care in heart failure management can benefit quality of life, symptom burden and levels of depression in patients with the condition [13].

Integrated palliative care aims to achieve continuity of care by integrating administrative, organizational, and clinical services that make up the patients care network [14]. Examples of integrated palliative care and heart failure interventions include collaborations and shared goal setting between palliative care and clinical cardiology teams to ameliorate symptoms with palliative care goals. alongside heart failure management [15]. In addition social work led palliative care services alongside heart failure management [16] aimed to improve the physical, psychological, social, spiritual, and end of life outcomes of patients. In 2020, the European Association for Palliative Care Task Force [17] concluded that the inclusion of

For peer review only

palliative care within the regular clinical framework for people with heart failure provides improvement in quality of life as well as comfort and dignity. This was echoed in a position paper by the Heart Failure Association of the European Society of Cardiology [18] who stated that many patients with heart failure would benefit from earlier integration of a palliative approach into the care provided by the multidisciplinary team involved.

However, although two decades have passed since the first publication on the benefits of palliative care for patients with advanced heart failure [19], the Heart Failure Association (HFA) Atlas identified only 10 out of 42 European countries with designated palliative care units for patients with advanced heart failure [20]. The poor integration of palliative care into advanced heart failure management can be explained by a number of factors, including uncertainty around the heart failure disease trajectory and complexities of communicating this uncertainty to patient and family members. Heart failure is an unpredictable illness, with periods of stability of symptoms, interjected with numerous exacerbations, of which many can lead to a temporary improvement in health status; others can lead to a progressive decline towards death. Many patients with advanced heart failure overestimate survival by up to 40%, further adding to cardiology providers' reluctance to initiate difficult conversations around prognosis. This difficulty is further compounded by lack of patient and practitioner knowledge around what palliative care is; misunderstanding that palliative care equals end-of-life care only. The fragmentation of inpatient and outpatient services also creates a barrier to the holistic need's assessment required for an integrated palliative care approach [21].

Overview of existing evidence

Until recently, the lack of evidence from clinical trials demonstrating benefits of palliative care for people with advanced heart failure posed an additional barrier. However, there has been an exponential increase in published literature since the turn of the century, increasing from 10 publications on average in 2000 to over 100 publications per year in 2017 (23). McIlvennan and Allen [21] published a review summarising the evolving role of palliative care for patients with heart failure, along with the barriers and opportunities for its integration into routine practice. Findings from the review highlighted the need for evidence on how best to integrate palliative care and heart failure given the cultural and environmental differences in how palliative care services are delivered. Three systematic reviews of palliative care interventions for patients with heart failure by Diop et al [22], Datla et al [13] and Sahlollbey et al [23] all highlighted the benefits of palliative care in heart failure management for

For peer review only

patient-centred outcomes and reducing hospital utilisation. A recent scoping review examining elements of integrated palliative care in heart failure management[24] identified the need for a multidisciplinary approach to integration and for cardiology staff to champion the benefits of palliative care. This review also highlighted the need for research with robust theoretical underpinnings given the complex behaviour changes required for sustaining integrated care in practice.

A recent editorial [25] exploring the phenomenon of inconsistent implementation of integrated palliative care and heart failure interventions proposed a realist approach could provide a sound theoretical understanding of the barriers and facilitators to routine implementation. The research to date has focused on "trying to evidence effectiveness through a linear cause and effect approach, which fails to ignore the messy, non-linear world of real-life practice" ([25], p.1). Overall, Datla et al. [26] concluded that there is no clear consensus around: 1) the core components of integrated palliative care and heart failure interventions, 2) the ideal configuration for the multi-disciplinary team, and 3) the most effective service provision model to ensure that generalist and/or specialist palliative care is tailored to patient needs

The issue of heterogeneity was further highlighted in a narrative literature review aimed at identifying the key characteristics of integrated palliative care and heart failure interventions [27]. Of the nine studies included, all integrated palliative care and heart failure interventions were implemented in different countries with different models of health service provision for citizens (USA, Sweden, Hong Kong), different settings (inpatient, outpatient and home-based), delivered by a heterogenous mix of multidisciplinary teams (cardiologists, heart failure-nurses, General Practitioners, community nurses, occupational therapists), using different modes of delivery (face-to-face, telemedicine), and involving different intervention components (symptom management, advance care planning). Therefore, we still do not know which intervention produces the best outcomes for patients and their informal carers (what works: specialist vs primary care etc.?), when best to initiate palliative care (for whom; at what stage in the disease trajectory?), or the optimal delivery method (in what circumstances; required infrastructure, staff competencies etc.?).

Rationale explaining why this research is important now.

Globally the population is living longer than ever before, and there are now 703 million individuals aged 65 or over in the world [28]. Projections estimate that those over 60 years

will increase from 12% of the world's population to 22% by 2050, with 1 in 6 individuals in the world being aged 60 or over by 2030 [29]. Although we can celebrate this achievement in life expectancy, it comes with significant challenges for healthcare now and in the future. Older people have complex health needs, with on average 4.5 comorbidities. Heart failure often dominates their physical and psychological needs [21], along with being the costliest aspect of their care due to high rates of hospitalisation and futile pharmaceutical and surgical interventions as their heart failure progresses [30]. Older people with advanced heart failure have undeniably had their needs overlooked, with calls for more attention to, and research for this vulnerable group to ensure they receive appropriate, effective treatment and care [21,31]. The 2021 European Society of Cardiology (ESC) Guidelines for the diagnosis and treatment of acute and chronic heart failure highlighted the need for studies to determine specific options for palliative care in the treatment of heart failure [32].

Although we have some promising examples of integrated palliative care and heart failure interventions [15,16], there is heterogeneity in terms of countries, healthcare settings, delivery by mix of multidisciplinary teams, modes of delivery and different intervention components [33]. Hence, this review is vital for identifying which model works best, for whom, or in what circumstances.

Aims and objectives

Aim

To understand how integrated palliative care and heart failure interventions may work in different healthcare settings e.g. inpatient/outpatient, and for which groups of people, so we can recommend strategies to maximise the potential for widespread implementation, reduce healthcare costs, and improve QoL for patients and informal carers

Objectives

1. To conduct a realist synthesis to build an understanding of which integrated palliative care and heart failure interventions work best, in which contexts and for which patients and informal carers
2. To co-produce recommendations with an expert stakeholder group, to maximise potential for widespread implementation through a user guide for healthcare providers and user friendly summaries for patients and the public.

For peer review only

Review questions

1. What are the mechanisms by which integrated palliative care and heart failure interventions work in order to produce their intended outcomes?

2. What are the contexts, which determine whether integrated palliative care and heart failure interventions produce their intended or unintended outcomes?

3. In what settings are integrated palliative care and heart failure interventions likely to be effective?

For peer review only

Methods and Analysis

Objective 1. Conduct a realist synthesis

This realist synthesis will follow Pawson's (2005) five iterative steps for realist synthesis (see figure 1). Realist syntheses are particularly suited to research on integrated palliative care and heart failure as they focus on making sense of the contextual factors that determine the outcomes of an intervention. Like other interventions that seek to propagate behavioural change, implementation of integrated palliative care and heart failure is highly context dependent, i.e. implementation of the same intervention will vary in its success depending, for example, on who delivers it and how it is delivered, the characteristics of the health care professionals involved, the circumstances surrounding it, and the tools and techniques used. Research designs that seek to "strip away" these contexts limit our understanding of "how, when and for whom" the intervention will be effective. A realist synthesis takes context as central to any explanation by exploring how an intervention manipulates context to trigger mechanisms that cause behavioural change.

The explanation building will start with the development of an initial programme theory of "how integrated palliative care and heart failure interventions produce desired outcomes (or not)". To achieve this, our preliminary initial realist programme theory sets out the necessary steps needed to accomplish the final intended outcome(s) from implementation of integrated palliative care and heart failure (see figure 2). This initial programme theory will be shaped through an iterative process of testing - i.e. parts of it are confirmed, refuted or refined against a range of relevant data from existing literature (see steps 1-5 below). This protocol has been registered with PROSPERO. The realist synthesis will adhere to the relevant quality and publication standards [33]. Figure 1 provides the project overview and key steps are outlined below in more detail.

Figure 1. Project flow diagram using Pawson's (2005) five iterative steps

INSERT FIGURE 1 HERE

For peer review only

Patient and public involvement

We will consult with UK wide and international stakeholders with both content and lived experience expertise, including NHS managers, and healthcare professionals (HCPs) involved in the delivery of palliative care and heart failure management, relevant charities, and patient and public involvement (PPI). Our initial programme theory will be refined throughout the course of the project based on regular consultation, via five virtual meetings and email correspondence with our stakeholder group. Our stakeholders, including PPI will also provide their perspectives on findings and recommendations, provide support with dissemination, and contribute to all project materials to ensure they meet the needs of patients and the wider public.

Step 1: Locate existing theories

The purpose of this step is to locate existing theories that explain why, how, in what contexts, for whom and to what extent does integrated palliative care and heart failure work to bring about improved outcomes for advanced heart failure patients and their informal carers. While we have already established there is limited theory underlying integrated palliative care and heart failure interventions, the realist synthesis approach allows for the literature net to be cast wider to include literature from other fields and other professions where potentially shared mechanisms may be in operation.

To identify these theories, we shall iteratively: a) consult with key content experts in our stakeholder group, and b) informally search the literature to identify existing theories. The informal searches conducted in step 1 differ from the more formal searching that will be carried in step 2 as their purpose is to quickly identify the kinds of theory that may be relevant; thus exploratory and informal search methods including, citation tracking and snow-balling based on known existing studies will be used. Once the theories have been identified, we shall build an initial programme theory (drawing on the preliminary initial programme theory- Refer Figure 2) to test in the synthesis. Programme theory development will necessitate iterative discussions within the research team to bring together the different theories into an initial programme theory.

For peer review only

Figure 2. Preliminary initial programme theory

INSERT FIGURE 2 HERE

Step 2: search for evidence

Formal search

The goal of Step 2 is to find a body of relevant literature in order to further develop and refine the initial programme theory developed in Step 1. The searches will be designed, piloted and carried out by an experienced Information Specialist (CD) with experience of carrying out iterative searches for realist synthesis. We will search academic databases including CINAHL, PsycINFO, PubMed, MEDLINE, AMED and EMBASE. A search for grey literature will also be carried out in sources including Google, OpenGrey, NICE, and Health Management Information Consortium (HMIC). Citation searching will also be undertaken including 'cited by' searches and searches of citations in the reference lists of relevant documents. We shall also ask the research team and steering group to identify any literature they may think is relevant. The databases will be searched using free text keywords and controlled vocabulary where appropriate, using terms such as advanced heart failure AND palliative care. The strategy will be informed by our informal scoping searches, search strategies employed in existing reviews and knowledge of the project team. Any literature that is likely to provide conceptually rich data, including grey literature, will be considered for inclusion in the synthesis.

For peer review only

Screening

When screening the identified literature, the inclusion (table 1) and exclusion criteria will be deliberately broad as we seek to identify quantitative, qualitative, mixed-methods and non-empirical documents.

Table 1. Inclusion criteria for identified literature

**Categories**	**Inclusion criteria**
Document types	All documents focused on palliative care for advanced heart failure patients.
Study design	All study designs. Non-empirical data (e.g. from opinion/commentary pieces) which help direct/shape theory development.
Types of settings	All documents about inpatient or outpatient or home-based care settings.
Types of participant	All adult patients (18 years and over). The majority of research has involved patients with advanced heart failure (defined as NYHA class III and IV).
Types of intervention	Any combination of palliative care strategies for the management of patients with advanced heart failure.
Outcome measures	All integrated palliative care and heart failure related outcome measures.

Table 1 shows the categories and details for inclusion criteria for identified literature.

The screening process will be piloted with small samples of documents until agreement on the application of the inclusion criteria is reached within the research team. CH will undertake screening of all titles and abstracts, and TM will independently review a 10% random sample of retrieved citations for quality control. If there are disagreements these will be resolved through discussion between members of the research team. In the event of disagreements remaining, these will be discussed and resolved by majority vote amongst the project research team. Documents included after title and abstract screening will then be screened again against inclusion criteria based on the full text. The same quality assurance process as described above will be used.

For peer review only

Additional searching

A vital part of conducting a realist synthesis involves the option to search for additional data to explain particular parts of the programme theory. Therefore, more searches will be conducted if we need more data to develop and test specific areas of the programme theory. Based on our understanding of integrated palliative care and heart failure to date, these could include areas like clinician/patient/informal carers attitude towards and understanding of palliative care, non-specialist palliative care education and training, having difficult conversations. These additional topics will increase the quantity of relevant data available for us to test the programme theory. The searches will be developed, piloted and refined by the Information Specialist (CD), along with the research team. These searches will differ from the 'formal searches' outlined above through being more exploratory and purposive, and from a range of different disciplines. Each additional search instigated, along with the inclusion and exclusion criteria, will be discussed by the research team to ensure alignment with objectives.

Step 3: Article selection

We are aware that there will be evidence that is of variable quality. However, it is because of this potential challenge that we have chosen to use a realist review approach. For example, full text documents will be selected based on relevance (whether data can contribute to theory building and/or testing) and rigour (whether methods used to generate the relevant data are credible and trustworthy). Assessments will also be made of the plausibility and coherence at the level of the programme theory [34]. To ensure consistency, a random sample of 10% of included papers will be assessed for relevance and discussed between TM and CH. CH will make decisions on the relevance and rigour of the remaining 90%. In the event of any uncertainty, the review team will discuss the relevance or rigour of the articles and any disagreements will be resolved through research team member discussions. A majority vote among the research team will be used to resolve any remaining disagreements.

Step 4: Extracting and organising data

CH will undertake organisation and data extraction. Full texts of the included articles will be imported into NVivo (a qualitative data management software tool). Relevant sections of texts relating to one or more parts of the programme theory will be coded in NVivo firstly by conceptual 'themes' and then as the synthesis progresses these will be developed into context-mechanism-outcome (CMO) configurations (see Step 5 below), the appropriateness

For peer review only

of which is outlined in the literature [33] . Data on the characteristics of the documents will be extracted separately into an Excel spreadsheet. As a quality assurance process, a random 10% of the coding will be independently checked by JR, with any disagreement resolved by discussion within the team.

Step 5: Synthesis the evidence and drawing conclusions

A realist logic of analysis will be used to analyse the data from included documents. Interpretive cross-case comparison will be used to understand and explain how and why actual outcomes have happened e.g. by comparing integrated palliative care and heart failure interventions that have been successful against those which have not, in order to understand how context has influenced reported findings [33]. CH will be supported by GW, CD and TM to undertake this step using the following reasoning processes typically used in synthesising evidence in realist synthesis:

a) Juxtaposition of sources of evidence e.g. where evidence about behaviour change in one source allows insights into evidence about outcomes in another source

b) Reconciling of sources of evidence – where results differ in similar situations, these will be further examined to find explanations for these differences

c) Adjudication of sources of evidence – centred on methodological strengths or weaknesses

d) Consolidation of sources of evidence – where different outcomes occur in similar contexts, a reason can be developed as to how and why these outcomes happen differently.

In all realist reviews there is always a risk that the review team will be swamped by the sheer breadth and detail they could cover. As is usual and expected practice in realist reviews (as seen in Item 3 of the RAMESES quality standards for realist syntheses: https://www.ramesesproject.org/media/RS_qual_standards_researchers.pdf), we will progressively focus the review.

This progressive focussing will help prioritise those aspects of the programme theory and/or CMOCs that are most important to stakeholders and/or provide the most useful findings that can be more readily actioned in the NHS. Such issues will be brought to stakeholder group meetings and combined with feedback and advice from the content and methodological expertise of the project team.

For peer review only

During the review we will seek out data on costs and sustainability. Where such data exist, if possible and relevant we will develop CMOCs for these aspects. Any such findings we develop on costs and sustainability of integrated palliative and heart failure services will be provided at the last two stakeholder meetings along with recommendations for commissioners and providers.

Objective 2.

To provide recommendations for commissioners, practitioners, patients, informal carers and the public.

Our programme theory will be used to provide recommendations for the optimal configuration and implementation of integrated palliative care and heart failure services across those NHS organisations currently implementing or wishing to implement an integrated palliative care and heart failure intervention. We will co-produce recommendations with our key stakeholders (commissioners, local, national, international content experts, multidisciplinary practitioners, patients and the public) as outlined below under our dissemination strategy, to ensure they are feasible, acceptable, and meaningful in practice. Recommendations will also inform specific guidelines for local, national and international professional bodies [35,36], which recommend an integrated palliative care and heart failure approach, on what works, for whom, how, why and in what contexts to bring about the desired outcomes.

For peer review only

Ethics and Dissemination

Dissemination

Our dissemination strategy will build on the co-production approach (involving PPI) that we have used throughout the synthesis process, which has included stakeholder involvement and engagement during the development of this research proposal and review process. Our approach will be consolidative, appreciating the varied forms of knowledge required to generate findings that are useful for informing decision making for policy, practice, and public audiences [37] follows:

1. Policymakers, decision-makers and commissioners of heart failure services.

2. NHS leaders, managers and practitioners involved in palliative care and heart failure provision

3. Members of the public, including those impacted by advanced heart failure

Different strategies will be employed based on the needs of each audience as follows:

1. Academic outputs, i.e. journal publications
2. A co-produced 'How to' user guide that outlines practical advice to optimise, tailor and implement interventions designed to integrate palliative care and heart failure that takes the local context into consideration.
3. User-friendly summaries of the synthesis findings using short animations to creatively convey complex findings. The intended impact of this creative approach is to widen the reach of the 'headline' findings of the realist synthesis to relevant stakeholders in a visually engaging way.

We will draw on the expertise within the stakeholder group to identify the key people for disseminating to each audience, ensuring outputs are tailored and relevant for their respective needs.

Ethics

Ethical approval is not required for this project and has been confirmed by the Research Governance and Ethics department at Queen's University Belfast.

For peer review only

Author Contributions

Tracey McConnell and Joanne Reid conceived the study. Tracey McConnell and Joanne Reid wrote the original protocol for this study with input from Loreena Hill, Geoff Wong and Claire Duddy. Clare Howie prepared the protocol as a manuscript for publication. John Burden, Bob Jones and Bob Ruane as members of the Patient and Public Involvement group provided input on drafting and editing of this protocol. All authors have read and approved the final manuscript.

Funding

This project is funded by the National Institute for Health Research (NIHR)[NIHR131800 Integrating Palliative Care and Heart Failure: a Realist Synthesis (PalliatHeartSynthesis)].

Disclaimer

The views expressed are those of the authors and not necessarily those of the NIHR or the Department of Health and Social Care.

Competing interests statement

Geoff Wong is Deputy Chair of the United Kingdom's National Institute of Health Research Health Technology Assessment Prioritisation Committee: Integrated Community Health and Social Care Panel (A) and a member of Methods Group (A). All other authors declare they have no competing interests.

For peer review only

References

1 World Health Organisation. WHO Global Status Report on Noncommunicable Diseases 2014. 2014. http://www.who.int/nmh/publications/ncd-status-report2014/en/

2 Ponikowski P, Anker SD, AlHabib KF, *et al.* Heart failure: preventing disease and death worldwide. *ESC Hear Fail* 2014;**1**:4–25. doi:10.1002/ehf2.12005

3 Savarese G, Lund LH. Global Public Health Burden of Heart Failure. *Card Fail Rev* 2017;**3**:7–11. doi:10.1007/s11886-002-0048-y

4 Ambrosy, Andrew P, Fonarow, Gregg C, Javed B, *et al.* The Global Health and Economic Burden of Hospitalizations for Heart Failure. *J Am Coll Cardiol* 2014;**63**:1123–33. doi:10.1016/j.jacc.2013.11.053

5 Cook C, Cole G, Asaria P, *et al.* The annual global economic burden of heart failure. *Int J Cardiol* 2014;**171**:368–76. doi:https://doi.org/10.1016/j.ijcard.2013.12.028

6 Morrison RS, Dietrich J, Ladwig S, *et al.* Palliative Care Consultation Teams Cut Hospital Costs For Medicaid Beneficiaries. *Health Aff* 2011;**30**:454–63. doi:10.1377/hlthaff.2010.0929

7 Gonzalez-Jaramillo V, Fuhrer V, Gonzalez-Jaramillo N, *et al.* Impact of home-based palliative care on health care costs and hospital use: A systematic review. *Palliat Support Care* 2021;**19**:474–87. doi:DOI: 10.1017/S1478951520001315

8 Sahlen K-G, Boman K, Brännström M. A cost-effectiveness study of person-centered integrated heart failure and palliative home care: Based on a randomized controlled trial. *Palliat Med* 2015;**30**:296–302. doi:10.1177/0269216315618544

9 End of Life Care. Report by the Comptroller and Auditor General [HC 1043 Session 2007-2008] London (2008) National Audit Office.

10 Doherty LC, Fitzsimons D, McIlfatrick SJ. Carers' needs in advanced heart failure: A systematic narrative review. *Eur J Cardiovasc Nurs* 2016;**15**:203–12. doi:10.1177/1474515115585237

11 Broese van Groenou MI, De Boer A. Providing informal care in a changing society. *Eur J Ageing* 2016;**13**:271–9. doi:10.1007/s10433-016-0370-7

12 Fitzsimons D, Doherty LC, Murphy M, *et al.* Inadequate Communication Exacerbates

For peer review only

the Support Needs of Current and Bereaved Caregivers in Advanced Heart Failure and Impedes Shared Decision-making. *J Cardiovasc Nurs* 2019;**34**.https://journals.lww.com/jcnjournal/Fulltext/2019/01000/Inadequate_Communication_Exacerbates_the_Support.5.aspx

13 Datla S, Verberkt CA, Hoye A, *et al.* Multi-disciplinary palliative care is effective in people with symptomatic heart failure: A systematic review and narrative synthesis. *Palliat Med* 2019;**33**:1003–16. doi:10.1177/0269216319859148

14 Siouta N, van Beek K, Preston N, *et al.* Towards integration of palliative care in patients with chronic heart failure and chronic obstructive pulmonary disease: a systematic literature review of European guidelines and pathways. *BMC Palliat Care* 2016;**15**:18. doi:10.1186/s12904-016-0089-4

15 Rogers JG, Chetan PB, Mentz RJ, *et al.* Palliative Care in Heart Failure. *J Am Coll Cardiol* 2017;**70**:331–41. doi:10.1016/j.jacc.2017.05.030

16 O'Donnell AE, Schaefer KG, Stevenson LW, *et al.* Social Worker–Aided Palliative Care Intervention in High-risk Patients With Heart Failure (SWAP-HF): A Pilot Randomized Clinical Trial. *JAMA Cardiol* 2018;**3**:516–9. doi:10.1001/jamacardio.2018.0589

17 Sobanski PZ, Alt-Epping B, Currow DC, *et al.* Palliative care for people living with heart failure: European Association for Palliative Care Task Force expert position statement. *Cardiovasc Res* 2020;**116**:12–27. doi:10.1093/cvr/cvz200

18 Hill L, Prager Geller T, Baruah R, *et al.* Integration of a palliative approach into heart failure care: a European Society of Cardiology Heart Failure Association position paper. *Eur J Heart Fail* 2020;**22**:2327–39. doi:10.1002/ejhf.1994

19 Anderson H, Ward C, Eardley A, *et al.* The concerns of patients under palliative care and a heart failure clinic are not being met. *Palliat Med* 2001;**15**:279–86. doi:10.1191/026921601678320269

20 Seferović PM, Jankowska E, Coats AJS, *et al.* The Heart Failure Association Atlas: rationale, objectives, and methods. *Eur J Heart Fail* 2020;**22**:638–45. doi:10.1002/ejhf.1768

21 McIlvennan CK, Allen LA. Palliative care in patients with heart failure. *Br Med J*

For peer review only

2016;**353**:i1010. doi:10.1136/bmj.i1010

22 Diop MS, Rudolph JL, Zimmerman KM, *et al.* Palliative Care Interventions for Patients with Heart Failure: A Systematic Review and Meta-Analysis. *J Palliat Med* 2016;**20**:84–92. doi:10.1089/jpm.2016.0330

23 Sahlollbey N, Kai Seng Lee C, Shirin A, *et al.* The impact of palliative care on clinical and patient-centred outcomes in patients with advanced heart failure: a systematic review of randomized controlled trials. *Eur J Heart Fail* 2020;**22**:2340–6.

24 Singh GK, Ivynian SE, Davidson PM, *et al.* Elements of Integrated Palliative Care in Chronic Heart Failure Across the Care Continuum: A Scoping Review. *Hear Lung Circ* 2021;:1–10. doi:10.1016/j.hlc.2021.08.012

25 McConnell T, Diffin J, Fitzsimons D, *et al.* Palliative care and heart failure: Can implementation science help where the evidence alone has failed? *Eur J Cardiovasc Nurs* 2019;**19**:190–1. doi:10.1177/1474515119894215

26 Datla S, Verberkt CA, Hoye A, *et al.* Multi-disciplinary palliative care is effective in people with symptomatic heart failure: A systematic review and narrative synthesis. *Palliat Med* 2019;**33**:1003–16. doi:10.1177/0269216319859148

27 Lewin WH, Schaefer KG. Integrating palliative care into routine care of patients with heart failure: models for clinical collaboration. *Heart Fail Rev* 2017;**22**:517–24. doi:10.1007/s10741-017-9599-2

28 United Nations Department of Economic and Social Affairs Population Division. *World Population Ageing 2019*. 2019. http://www.un.org/esa/population/publications/worldageing19502050/pdf/65executive summary spanish.pdf%0Ahttp://link.springer.com/chapter/10.1007/978-94-007-5204-7_6

29 Ageing and Health. World Health Organisation. 2018.https://www.who.int/news-room/fact-sheets/detail/ageing-and-health

30 Braunschweig F, Cowie MR, Auricchio A. What are the costs of heart failure? *EP Eur* 2011;**13**:ii13–7. doi:10.1093/europace/eur081

31 Hill L, Carson MA, Vitale C. Care plans for the older heart failure patient. *Eur Hear J Suppl* 2019;**21**:L32–5. doi:10.1093/eurheartj/suz243

For peer review only

32 McDonagh TA, Metra M, Adamo M, *et al.* 2021 ESC Guidelines for the diagnosis and treatment of acute and chronic heart failure. *Eur Heart J* 2021;:1–128. doi:10.1093/eurheartj/ehab368

33 Wong G, Greenhalgh T, Westhorp G, *et al.* RAMESES publication standards: realist syntheses. *BMC Med* 2013;**11**:21. doi:10.1186/1741-7015-11-21

34 Emmel N, Greenhalgh J, Manzano A, Monaghan M, Dalkin S. *Doing Realist Research.* London: : Sage 2018.

35 National Institute for Care and Excellence (NICE). Chronic heart failure in adults. NICE Quality Standard [QS9]. 2011.

36 Braun LT, Grady KL, Kutner JS, *et al.* Palliative Care and Cardiovascular Disease and Stroke: A Policy Statement From the American Heart Association/American Stroke Association. *Circulation* 2016;**134**:e198–225. doi:10.1161/CIR.0000000000000438

37 Bowen S, Graham I. Integrated Knowledge Translation. In: *Knowledge Translation in Health Care*. John Wiley & Sons 2013. 14–23.

For peer review only

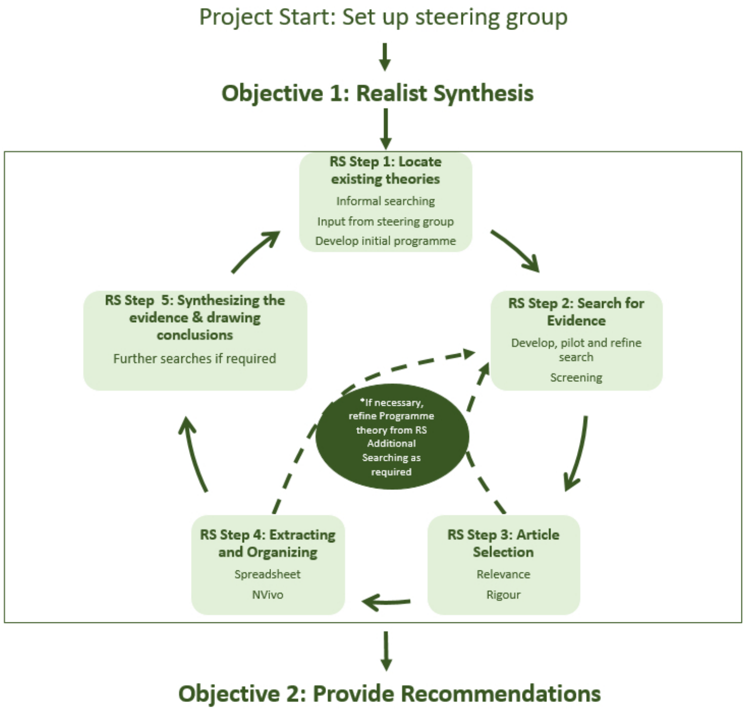

Figure 1. Project flow diagram using Pawson's (2005) five iterative steps

656x476mm (38 x 38 DPI)

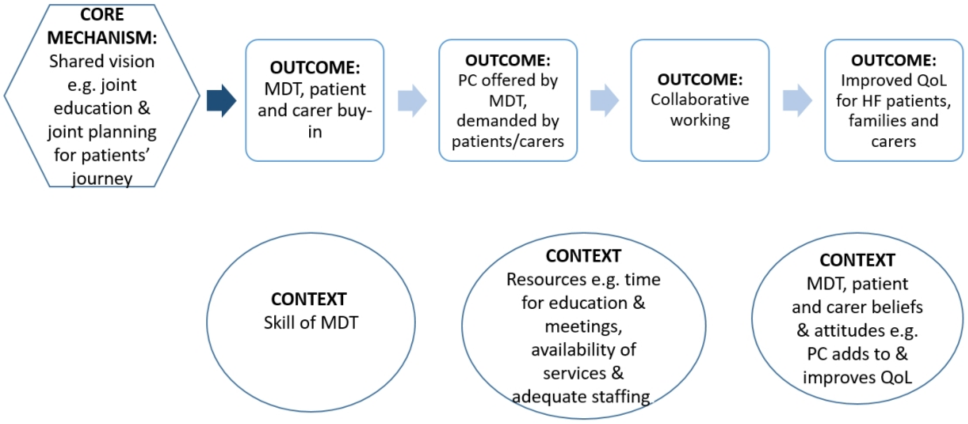

Figure 2. Preliminary initial programme theory

940x425mm (38 x 38 DPI)

Wong *et al. BMC Medicine* 2013, **11**:21
http://www.biomedcentral.com/1741-7015/11/21

BMC Medicine

GUIDELINE Open Access

RAMESES publication standards: realist syntheses

Geoff Wong[1*], Trish Greenhalgh[1], Gill Westhorp[2], Jeanette Buckingham[3] and Ray Pawson[4]

Abstract

Background: There is growing interest in realist synthesis as an alternative systematic review method. This approach offers the potential to expand the knowledge base in policy-relevant areas - for example, by explaining the success, failure or mixed fortunes of complex interventions. No previous publication standards exist for reporting realist syntheses. This standard was developed as part of the RAMESES (Realist And MEta-narrative Evidence Syntheses: Evolving Standards) project. The project's aim is to produce preliminary publication standards for realist systematic reviews.

Methods: We (a) collated and summarized existing literature on the principles of good practice in realist syntheses; (b) considered the extent to which these principles had been followed by published syntheses, thereby identifying how rigor may be lost and how existing methods could be improved; (c) used a three-round online Delphi method with an interdisciplinary panel of national and international experts in evidence synthesis, realist research, policy and/or publishing to produce and iteratively refine a draft set of methodological steps and publication standards; (d) provided real-time support to ongoing realist syntheses and the open-access RAMESES online discussion list so as to capture problems and questions as they arose; and (e) synthesized expert input, evidence syntheses and real-time problem analysis into a definitive set of standards.

Results: We identified 35 published realist syntheses, provided real-time support to 9 on-going syntheses and captured questions raised in the RAMESES discussion list. Through analysis and discussion within the project team, we summarized the published literature and common questions and challenges into briefing materials for the Delphi panel, comprising 37 members. Within three rounds this panel had reached consensus on 19 key publication standards, with an overall response rate of 91%.

Conclusion: This project used multiple sources to develop and draw together evidence and expertise in realist synthesis. For each item we have included an explanation for why it is important and guidance on how it might be reported. Realist synthesis is a relatively new method for evidence synthesis and as experience and methodological developments occur, we anticipate that these standards will evolve to reflect further methodological developments. We hope that these standards will act as a resource that will contribute to improving the reporting of realist syntheses.
To encourage dissemination of the RAMESES publication standards, this article is co-published in the Journal of Advanced Nursing and is freely accessible on Wiley Online Library (http://www.wileyonlinelibrary.com/journal/jan).
Please see related article http://www.biomedcentral.com/1741-7015/11/20 and http://www.biomedcentral.com/1741-7015/11/22

Keywords: realist synthesis, realist review, publication standards

Background

Academics and policymakers are increasingly interested in 'policy-friendly' approaches to evidence synthesis. Such approaches seek to illuminate issues and understand contextual influences on whether, why and how interventions might work [1,2]. A number of different approaches have been used to try to achieve this goal. At present there is lack of clarity on which methods are best suited for which questions or problems and this has been the subject of debate [3-6] and further research [7]. Realist synthesis is a theory-driven approach that is becoming increasingly popular.

* Correspondence: grckwong@gmail.com
[1]Centre for Primary Care and Public Health, Queen Mary University of London, 58 Turner Street, London E1 2AB, UK
Full list of author information is available at the end of the article

BioMed Central
© 2013 Wong et al; licensee BioMed Central Ltd. This is an open access article distributed under the terms of the Creative Commons Attribution License (http://creativecommons.org/licenses/by/2.0), which permits unrestricted use, distribution, and reproduction in any medium, provided the original work is properly cited.

Wong et al. BMC Medicine 2013, 11:21
http://www.biomedcentral.com/1741-7015/11/21

What is a realist synthesis?

In this section we briefly describe the realist synthesis method. The realist research question is often summarized as "What works for whom under what circumstances, how and why?" Realist inquiry is based on a realist philosophy of science and considers the interaction between context, mechanism and outcome. From a realist perspective, intervention X is not thought of as having effect size Y with confidence interval Z. Rather, intervention X (for example, a program introduced by policymakers who seek to create a particular outcome) alters context (for example, by making new resources available), which then triggers mechanism(s), which produce both intended and unintended outcomes. Intervention X may work well in one context but poorly or not at all in another context.

Realist inquiry seeks to unpack the context - mechanism - outcome relationship, thereby explaining examples of success, failure and various eventualities in between. Theoretical explanations of this kind are referred to as "middle-range theories" (that is, ones which "...involve abstraction... but [are] close enough to observed data to be incorporated in propositions that permit empirical testing" [8].

The basis of realist inquiry is a realist philosophy, whose key tenets are as follows:

1. There is a [social] reality that cannot be measured directly (because it is processed through our brains, language, culture and so on), but can be known indirectly.

Realism thus sits, broadly speaking, between positivism ('there is a real world which we can apprehend directly through observation') and constructivism ('given that all we can know has been interpreted through human senses and the human brain, we cannot know for sure what the nature of reality is').

2. Social programs (including complex interventions) may change the macro social context (for example, by introducing legislation). They may also change the resources or opportunities available to participants and, in that sense, change the meso- or micro-level context for those participants.

3. To understand the relationship between context and outcome, realism uses the concept of mechanisms, one definition of which is "...underlying entities, processes, or [social] structures which operate in particular contexts to generate outcomes of interest" [9].

In common with other theory-driven review methods, the realist approach offers the potential for insights that go beyond the narrowly experimental paradigm of the randomized controlled trial [10-12]. It can do so in relation to complex, complicated or simpler interventions (for example, even a simple intervention, such as a drug, is prescribed, dispensed and taken - or not - in a particular social, cultural and economic context).

"Realist synthesis" was first described by Ray Pawson in 2002 [13], updated in an ESRC (Economic and Social Research Council) commissioned monograph in 2004 [14], published as a book in 2006 [1] and summarizsed in a short methods paper in 2005 [15]. Since this paper is deliberately focused on publication standards, we strongly recommend that those unfamiliar with the realist approach consult these or other relevant methodological sources.

A realist synthesis (or realist review - these terms are synonymous) applies realist philosophy to the synthesis of findings from primary studies that have a bearing on a single research question or set of questions. Methodologically, reviewers may begin by eliciting from the literature the main ideas that went into the making of a class of interventions (the program theory). This program theory sets out how and why a class of intervention is thought to 'work' to generate the outcome(s) of interest. The pertinence and effectiveness of each constituent idea is then tested using relevant evidence (qualitative, quantitative, comparative, administrative and so on) from the primary literature on that class of programs. In this testing, the ideas within a program theory are re-cast and conceptualized in realist terms.

For each idea, reviewers seek out the contextual (C) influences that are hypothesized to have triggered the relevant mechanism(s) (M) to generate the outcome(s) (O) of interest. Synthesis consists of comparing 'how the programme was supposed to operate' to the 'empirical evidence on the actuality in different situations' - all along C-M-O lines. Analytic purchase comes from the ability to describe and understand the many contingencies that affect the likelihood of such interventions generating their intended outcomes. This in turn provides guidance about what policy makers or practitioners might put in place to change the context or provide resources in such a way as to most likely trigger the right mechanism(s) to produce the desired outcome.

Why are publication standards needed?

Publication standards are common (and, increasingly, expected) - in health services research - see, for example, CONSORT for randomized controlled trials [16], AGREE for clinical guidelines [17], PRISMA for Cochrane-style systematic reviews [18] and SQUIRE for quality improvement studies [19]. For realist syntheses, publication standards are particularly important as this method is relatively new and concerns have been expressed about the rigor with which some realist reviews have been carried out and reported [20]. Publication standards are needed to ensure that users of reviews are provided with relevant and necessary information to enable them to assess the quality and rigor of a review.

In our experience, there is considerable confusion among researchers, journal editors, peer reviewers and

For peer review only - http://bmjopen.bmj.com/site/about/guidelines.xhtml

funders about what counts as a high quality realist review and what, conversely, counts as a flawed review. Even though experts still differ on detailed conceptual methodological issues, the increasing popularity of this method prompted a study to develop baseline standards from which, we anticipate, further developments in theory and methodology of this approach will occur.

Aim

The aim of this paper is to produce preliminary publication standards for realist syntheses.

Methods

The methods we used to develop these reporting standards have already been published [20]. In brief, we purposively recruited an international group of experts to our online Delphi panel. Aiming to achieve maximum variety in the relevant sectors, disciplines and expert perspectives represented, we sought panel members working in realist research, evidence synthesis, publication, reviewer training and health policy. Prior to the start of our Delphi panel, with input from an expert informaticist (JB), we collated and summarized existing literature on the principles of good practice in realist synthesis, created a database of such published syntheses, and built relationships with teams who were undertaking ongoing syntheses. Through discussion within the project team, we considered the extent to which the principles had been followed by published and in-progress reviews, thereby identifying how rigor may be lost and how existing methods could be improved.

Our analysis of existing realist syntheses formed the basis of the briefing materials for the first round of the Delphi panel. In addition, we drew on our collective experience in training and supporting realist syntheses teams and an email discussion list on realist and meta-narrative methodology [21] to further inform the contents of our briefing document. Both the research team and panel members contributed draft items for the publication standards, and these were refined using the online Delphi process as previously described [20]. We ran the Delphi panels between September 2011 and March 2012.

Description of panel and items

In all, we recruited 37 individuals from 27 organizations in 6 countries. These comprised: researchers in public or population health researchers (8); evidence synthesis (6); health services research (8); international development (2); education (2); and also research methodologists (6), publishing (1), nursing (2) and policy and decision making (2). In round 1, 22 Delphi panel members provided suggestions of items that should be included in the publication standards. In rounds 2 and 3 our panel members were asked to rate each potential item for relevance and clarity. The response rates across all items for rounds 2 and 3 were 93% and 89%, respectively. Consensus was reached within three rounds on both the content and wording of 19 items within the publication standards. Table 1 provides an overview of these items.

Scope of the publication standards

These publication standards are intended to help researchers, authors, journal editors, and policy and decision makers to know and understand what should be reported in the write-up of a realist synthesis. They are not intended to provide detailed guidance on how to conduct such a synthesis; for this, we direct interested readers to summary articles [15,22] or various publications on methods [1,11,14,23]. This publication standard applies only to realist syntheses. A list of publication guidelines for other review methods can be found on the EQUATOR Network's website [24], but at present none of these relate specifically to realist syntheses. As part of the RAMESES project we are also developing quality standards and training materials for realist syntheses, which will be submitted as a separate publication. Publication standards for meta-narrative reviews (also covered in the RAMESES project) have been addressed in a separate article.

How to use these publication standards

The layout of this document has drawn on previous methodological publications and, in particular, on the 'Explanations and Elaborations' document of the PRISMA statement [18]. Each item is followed by an example drawn from published reviews and a rationale for its inclusion. The purpose of the example text is to illustrate how an item might be reported in a write up. However, potentially relevant contextual information may have been omitted, so it may be necessary to consult the original paper from which the example text was drawn. The standards set out what might be expected for each item, but authors will still need to exercise judgement about how much information to include. The purpose of the details reported should be to ensure that the description and explanation provided is coherent and plausible, both against the guidance set out within an item and for the overall purpose of the realist synthesis.

While this publication standard is modeled on the PRISMA statement, the items within are not identical. This publication standard, developed to apply only to realist syntheses, has some overlap with the PRISMA statement. Items 1 to 3, 15, 16 and 19 in this statement broadly match the purpose of items 1 to 3, 24, 25 and 27 in the PRISMA statement. For items 4 to 14, while there is some overlap in purpose with some PRISMA statement items, different or additional reporting is needed due to the nature of realist syntheses. Other items (5, 12, 13, 15, 16, 19

For peer review only

Wong et al. BMC Medicine 2013, 11:21
http://www.biomedcentral.com/1741-7015/11/21

Table 1 List of items to be included when reporting a realist synthesis

**TITLE**	
1	In the title, identify the document as a realist synthesis or review
ABSTRACT	
2	While acknowledging publication requirements and house style, abstracts should ideally contain brief details of: the study's background, review question or objectives; search strategy; methods of selection, appraisal, analysis and synthesis of sources; main results; and implications for practice.
INTRODUCTION	
3 Rationale for review	Explain why the review is needed and what it is likely to contribute to existing understanding of the topic area.
4 Objectives and focus of review	State the objective(s) of the review and/or the review question(s). Define and provide a rationale for the focus of the review.
METHODS	
5 Changes in the review process	Any changes made to the review process that was initially planned should be briefly described and justified.
6 Rationale for using realist synthesis	Explain why realist synthesis was considered the most appropriate method to use.
7 Scoping the literature	Describe and justify the initial process of exploratory scoping of the literature.
8 Searching processes	While considering specific requirements of the journal or other publication outlet, state and provide a rationale for how the iterative searching was done. Provide details on all the sources accessed for information in the review. Where searching in electronic databases has taken place, the details should include, for example, name of database, search terms, dates of coverage and date last searched. If individuals familiar with the relevant literature and/or topic area were contacted, indicate how they were identified and selected.
9 Selection and appraisal of documents	Explain how judgements were made about including and excluding data from documents, and justify these.
10 Data extraction	Describe and explain which data or information were extracted from the included documents and justify this selection.
11 Analysis and synthesis processes	Describe the analysis and synthesis processes in detail. This section should include information on the constructs analyzed and describe the analytic process.
RESULTS	
12 Document flow diagram	Provide details on the number of documents assessed for eligibility and included in the review with reasons for exclusion at each stage as well as an indication of their source of origin (for example, from searching databases, reference lists and so on). You may consider using the example templates (which are likely to need modification to suit the data) that are provided.
13 Document characteristics	Provide information on the characteristics of the documents included in the review.
14 Main findings	Present the key findings with a specific focus on theory building and testing.
DISCUSSION	
15 Summary of findings	Summarize the main findings, taking into account the review's objective(s), research question(s), focus and intended audience(s).
16 Strengths, limitations and future research directions	Discuss both the strengths of the review and its limitations. These should include (but need not be restricted to) (a) consideration of all the steps in the review process and (b) comment on the overall strength of evidence supporting the explanatory insights which emerged. The limitations identified may point to areas where further work is needed.
17 Comparison with existing literature	Where applicable, compare and contrast the review's findings with the existing literature (for example, other reviews) on the same topic.
18 Conclusion and recommendations	List the main implications of the findings and place these in the context of other relevant literature. If appropriate, offer recommendations for policy and practice.
19 Funding	Provide details of funding source (if any) for the review, the role played by the funder (if any) and any conflicts of interests of the reviewers.

and 23) in the PRIMSA statement have no equivalent in the RAMESES publication standards for realist reviews.

The order in which items are reported may vary. Realist syntheses are not 'linear' reviews. Some of the processes that are listed may legitimately take place in parallel or have to be revisited at a later date as a review progresses. As a general rule, if a recommended item is excluded from the write-up of a realist synthesis, a justification should be provided.

The RAMESES publication standards for realist syntheses

Item 1: Title

In the title, identify the document as a realist synthesis or review.

For peer review only - http://bmjopen.bmj.com/site/about/guidelines.xhtml

Example

"Human resource management interventions to improve health workers' performance in low and middle income countries: a realist review." [25]

Explanation

Our background searching has shown that some realist reviews are not flagged as such in the title and may also be inconsistently indexed and, hence, are more difficult to locate during searching. The terms 'realist synthesis' and 'realist review' are both in widespread use. We asked our Delphi panel if they had a preferred term - 'realist synthesis' or 'review'. No consensus was reached by our Delphi panel on whether 'review' or 'synthesis' should be the preferred term, and there seemed no good reason to impose one or other term.

Item 2: Abstract

While acknowledging that requirements and house style may differ between journals, abstracts should ideally contain brief details of the study's background, review question or objectives; search strategy; methods of selection, appraisal, analysis and synthesis of sources; main results; and implications for practice.

Example

"Background

Legislation is one of the most powerful weapons for improving population health and is often used by policy and decision makers. Little research exists to guide them as to whether legislation is feasible and/or will succeed. We aimed to produce a coherent and transferable evidence based framework of threats to legislative interventions to assist the decision making process and to test this through the 'case study' of legislation to ban smoking in cars carrying children.

Methods

We conceptualised legislative interventions as complex social interventions and so used the realist synthesis method to systematically review the literature for evidence. 99 articles were found through searches on five electronic databases (MEDLINE, HMIC, EMBASE, PsychINFO, Social Policy and Practice) and iterative purposive searching. Our initial searches sought any studies that contained information on smoking in vehicles carrying children. Throughout the review we continued where needed to search for additional studies of any type that would conceptually contribute to helping build and/or test our framework.

Results

Our framework identified a series of transferable threats to public health legislation. When applied to smoking bans in vehicles; problem misidentification, public support; opposition; and enforcement issues were particularly prominent threats. Our framework enabled us to understand and explain the nature of each threat and to infer the most likely outcome if such legislation were to be proposed in a jurisdiction where no such ban existed. Specifically, the micro-environment of a vehicle can contain highly hazardous levels of second hand smoke. Public support for such legislation is high amongst smokers and non-smokers and their underlying motivations were very similar - wanting to practice the Millian principle of protecting children from harm. Evidence indicated that the tobacco industry was not likely to oppose legislation and arguments that such a law would be 'unenforceable' were unfounded.

Conclusion

It is possible to develop a coherent and transferable evidence based framework of the ideas and assumptions behind the threats to legislative intervention that may assist policy and decision makers to analyse and judge if legislation is feasible and/or likely to succeed." [26]

Explanation

Apart from the title, an abstract is the only source of information accessible to searchers unless the full paper is obtained. The information in it must allow reviewers and/or users to decide if the review is relevant to their needs.

Introduction section

The following items should be reported in the introduction section.

Item 3: Rationale for review

Explain why the review is needed and what it is likely to contribute to existing understanding of the topic area.

Example

"A number of reviews on the subject have tried to examine evidence to improve the operationalization of interventions by CHWs [community health workers], including for child health. Lehmann *et al.* (Reference x1) and Lewin *et al.* (Reference x1) have reviewed evidence on CHW interventions in LMIC [low-middle income countries] and Haines *et al.* (Reference x1) have particularly so for child health. Lewin *et al.* (Reference x1) found lay health workers to be effective in specific areas in child health, when compared to usual care. Haines *et al.* (Reference x1) highlight the contextual nature of CHW's performance. Both caution that CHW interventions are not the panacea for all that ails the health systems in LMIC and that large scale CHW programmes should be initiated with great caution. Both raise questions about the applicability of findings to different settings and about the conditions under which CHW interventions should be implemented." [27]

For peer review only

Wong et al. BMC Medicine 2013, 11:21
http://www.biomedcentral.com/1741-7015/11/21

Explanation

As with all research, a background section explaining what is already known and what the researchers considered to be the 'knowledge gaps' is a helpful orientation.

Item 4: Objectives and focus of review

State the objective(s) of the review and/or the review question(s). Define and provide a rationale for the focus of the review.

Example

"The overriding question for the review was: Does moving from high-poverty neighborhoods to lower-poverty neighborhoods improve health? More specifically: What were the key health outcomes? Who experienced these outcomes? What appeared to be the mechanisms and associated context leading to the outcomes? As the review proceeded, it became clear that one of the only relatively consistent and statistically significant positive health outcomes was an improvement in mental health for adult women, children and adolescent girls. In this paper a review of mental health outcomes of MTO [Moving To Opportunity] is presented, along with some insights about the mechanisms and contexts through which the intervention appears to have impacted mental health." [28]

Explanation

A realist research question contains some or all of the elements of 'What works, how, why, for whom, to what extent and in what circumstances, in what respect and over what duration?' and applies realist logic to address the question (see Item 11).

Because a realist synthesis may generate a large number of avenues that might be explored and explained, and because resources and timescale are invariably finite, the expectation is that the review must be 'contained' by progressively focusing both its breadth (how wide an area?) and depth (how much detail?). This important process may involve discussion and negotiation with, for example, content experts, funders and/or users. It is typical and legitimate for the synthesis' objectives, question and/or the breadth and depth of the review to evolve as the review progresses. How and why it evolved is usually worth reporting.

Methods section

The following items should be reported in the methods section.

Item 5: Changes in the review process

Any changes made to the review that was initially planned should be briefly described and justified.

Example

"As the review progressed we became aware of various data suitability limitations (see Discussion) and the emergence of two prominent demi-regularities prompted us to narrow our review focus to the two candidate theories discussed below." [29]

Explanation

A realist synthesis can (and, in general, should) evolve over the course of the review. For example, changes to the research question or its scope are likely to have an impact on many of the synthesis' subsequent processes. However, this does not mean the synthesis can meander uncontained. An accessible summary of what was originally planned (for example, as described in an initial protocol) and how and why this differed from what was done should be provided as this may assist interpretation.

Item 6: Rationale for using realist synthesis

Explain why realist synthesis was considered the most appropriate method to use.

Example

"Previous reviews sought to understand PR [participatory research] and provide practical recommendations (References x6) and to assess the value of PR to research goals, health status, and systems change (References x6). Nonetheless, the assessment of outcomes remains weak (Reference x4), partly because the methodologies used have generally failed to embrace the complexity of programs or address mechanisms of change (Reference x1). ...

To handle such complexity, we chose a realist approach (Reference x1) because it provides a rationale and tools for synthesizing complex, difficult-to-interpret evidence from community-based programs." [30]

Explanation

Realist synthesis is a theory-driven method that is firmly rooted in a realist philosophy of science. It places particular emphasis on understanding causation (in this case, understanding how programs and policies generate outcomes through human decisions) and how causal mechanisms are shaped and constrained by social context. This makes it particularly suitable for reviews of certain topics and questions - for example, complex social programs that involve human decisions and actions. It also makes realist synthesis *less* suitable than other review methods for certain topics and questions - for example, those which seek primarily to determine the average effect size of a simpler intervention administered in a single or limited range of conditions. In our analysis of 37 published realist syntheses, the most common limitation was inadequate engagement with realist explanatory principles and the implications these have, first, for understanding programs and how they work, and second, for cumulating evidence and explanation.

Some realist syntheses published to date have deliberately adapted the method as first described by Pawson.

For peer review only

Sometimes, adaptations may be entirely justifiable, but at other times they may indicate a poor grasp of realist methodology. To enable judgement to be made on adaptations, the description and rationale for adaptations should be provided. Such information will allow criticism, debate and counter criticism among review teams and users on the suitability of such adaptations, and may well facilitate methodological development.

Item 7: Scoping the literature

Describe and justify the initial process of exploratory scoping of the literature.

Example

"To develop our framework on the threats to the programme theory of public health legislation we started out by conducting a rapid review of broad areas of public health legislation (covering everything from gun amnesties to food labelling) trying to uncover what had been the sticking points in legislation and how (if at all) they had been circumvented. This outline review led to the construction of a provisional framework for reviewing the family of legislative interventions (as described in Figure 1). .... . Beginning with this framework and through discussions (and with reference to other interested stakeholders) we focused on a subset of themes that seemed most relevant in respect to the intervention in question. In our case, we deliberately sought input from the NICE officer seconded to our project." [26]

Explanation

This step is used to build an understanding of the topic area. For example, this step may be used to identify provisional program theories, the names/titles of programs within scope and key authors in the area. Initial attempts to make sense of a topic area may involve informal 'browsing' of the literature and also consulting with experts and stakeholders.

Item 8: Searching process

While considering specific requirements of the journal or other publication outlet, state and provide a rationale for how the iterative searching was done. Provide details on all the sources accessed for information in the synthesis. For example, where electronic databases have been searched, details should include, for example, the name of the database, search terms, dates of coverage and date last searched. If individuals familiar with the relevant literature and/or topic area were contacted, indicate how they were identified and selected.

Example

"...the literature search was iterative and ongoing throughout the project. An initial search was conducted of various academic databases, such as Academic Search Premier, Arts and Humanities Citation Index, Canadian Research Index, as well as through various search engines, such as Prowler, Novanet, Google and Google Scholar. Search terms included: Moving to Opportunity [MTO]; housing intervention; housing mobility; housing health effects; low-

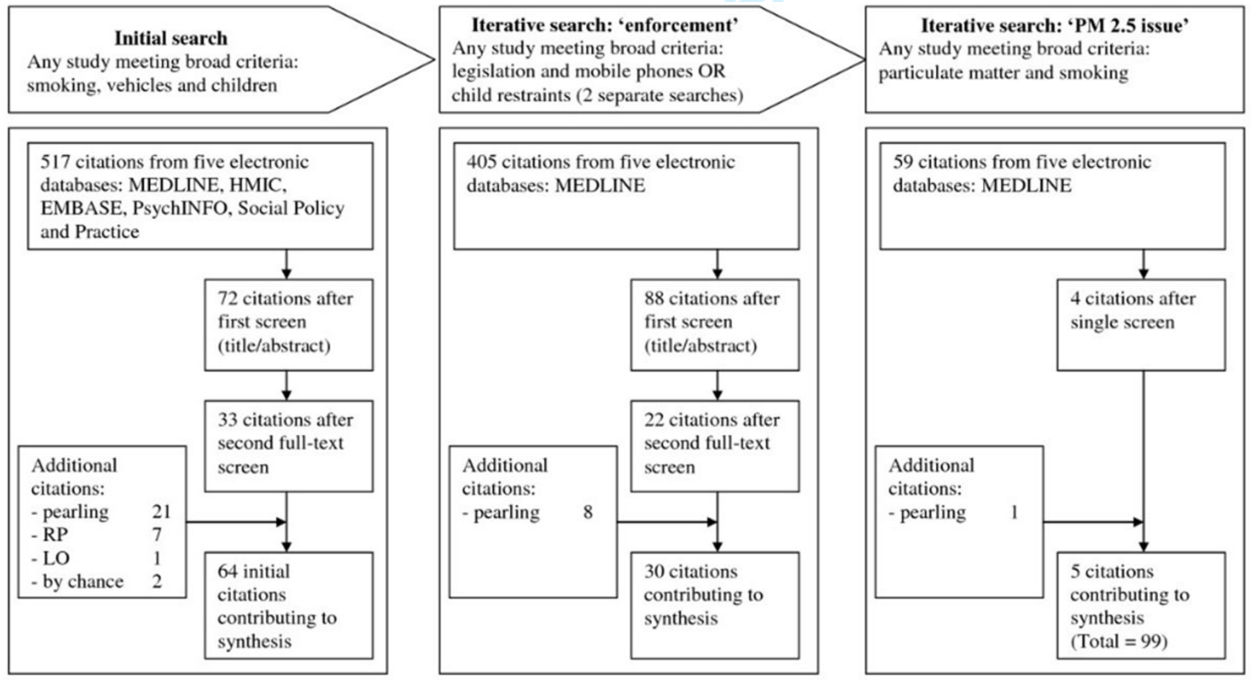

Figure 1 Flow diagram illustrating search process and article disposition [26].

For peer review

Wong et al. BMC Medicine 2013, 11:21
http://www.biomedcentral.com/1741-7015/11/21

poverty neighborhood/community; high-poverty neighborhood/community; neighborhood/community health; poverty neighborhood/community; poverty community effects; poverty housing; poverty health; and housing health. A "snowball" approach was used in which one reference led to others. Other evaluations were revealed through correspondence with Dr. Jeffrey Kling, one of the principal MTO researchers." [28]

Explanation

Searching should be guided by the objectives and focus of the synthesis, and revised iteratively in the light of emerging data. Data relevant to a realist synthesis may lie in a broad range of sources that may cross traditional disciplinary, program and sector boundaries. The search phase is thus likely to involve searching for different sorts of data, or studies from different domains, with which to test different aspects of any provisional theory.

Search methods using forward and backward citation tracking may be particularly valuable in finding the documents necessary to develop and then test provisional theories. Realist syntheses do not exclude sources solely on the basis of their study design; hence, 'methodological filters' (for example, to identify randomized controlled trials) may add little to the search and could potentially miss relevant papers.

Searching is likely to be iterative because, as the synthesis progresses, new or refined elements of theory may be required to explain particular findings, or to examine specific aspects of particular processes. As new elements of theory are included, searches for evidence to support, refute or refine those elements may be required. If undertaken, the process used for any such additional searches should be clearly documented. A single pre-defined search is unlikely to be sufficient and may suggest insufficient reflection on emerging findings.

Sufficient detail should be given to enable the reader to judge whether searching was likely to have located sources needed for theory building and/or testing.

Item 9: Selection and appraisal of documents

Explain how judgements were made about including and excluding data from documents, and justify these.

Example

"Three tools were developed (for identification, selection, and appraisal) in March, June, and October 2009, respectively. Modifications were made during each stage after piloting. Each stage processed a different type of data: citations in identification; full-text papers in selection; and sets of publications in appraisal.

...The identification tool consisted of three questions. This step funnelled the number of citations from 7,167 to 594.

The librarian (JH) retrieved the 594 full-text papers, which were read by two independent reviewers, using a selection tool initially comprised of six questions in June 2009, with an additional two questions added in October 2009. ...

Two hundred articles remained from 594 after filtering them through the selection tool. Due to the complexity of the dataset, we decided at this stage to further limit the scope of our review to community-based settings, and to participatory interventions. Our rationale was that: PR in all forms (community-based PR, organizational PR, action research) was too diverse to be assessed within one review; the complexity of PR benefits from community-based research provided a manageable set of studies; intervention research demonstrated more complexity of outcomes than non-intervention research, and would be best suited for analysis using realist review methods; and the pool of studies needed to be reduced to a manageable size for an in depth realist synthesis (analysis). Adding two questions reduced the pool to 83 studies.....

Contact with principal investigators of all full-text papers retained after selection was undertaken because descriptions of programs, methods and findings of PR interventions were found to be commonly described across a number of publications pertaining to the same intervention. It was thus necessary to confirm that we had complete sets of papers in order to fairly appraise projects according to the realist review approach. ... For each study, we then sent our list of papers to the corresponding author or PI, and asked them to confirm that we had the complete set, or to send us additional documents. ... Only those sets of studies in which the contacted researcher responded to our request were retained for appraisal.

.... The appraisal tool consisted of three questions. An additional 11 sets were eliminated after screening with the tool below, which left a total of 23 sets, comprising 276 documents that were retained for synthesis. See Appendix 4: ... for a complete breakdown of the number of cases retained at each stage." [30]

Explanation

Realist synthesis is not a technical process - that is, following a set protocol will not guarantee that a review will be robust. Rather, it requires a series of judgements about the relevance and robustness of particular data for the purposes of answering a specific question.

Within any document, there may be several pieces of data that serve different purposes, such as helping to build one theory, refining another theory and so on. Therefore, the selection (for inclusion or exclusion) and appraisal of the contribution of pieces of data within a document cannot be based on an overall assessment of study or

For peer review only - http://bmjopen.bmj.com/site/about/guidelines.xhtml

document quality. An appraisal of the contribution of any section of data (within a document) should be made on two criteria:

- *Relevance* - whether it can contribute to theory building and/or testing; and
- *Rigor* - whether the method used to generate that particular piece of data is credible and trustworthy.

A wide range of documents can potentially contribute to a realist synthesis. For example, outcome and impact studies, qualitative interviews, ethnography, questionnaire surveys, mixed-method case studies, and close reading of policies, business plans, websites, project initiation documents and 'gray literature' write-ups of programs may all contribute in different ways of identifying and elucidating program theories. Because of this range and realist review's focus on relevance and rigor, it can initially be difficult to 'whittle down' the number of documents that are potentially eligible for inclusion in a review. This process can only occur as the data sources are analyzed in detail. Thus, in practice, the selection and appraisal stage may need to run in parallel with the analysis stage.

It is unlikely that authors will be able to provide an in-depth description of each decision involved, but the broad processes used to determine relevance and assess rigor (for example, using quality standards appropriate to particular kinds of research to appraise documents or sections of documents; discussion and/or debate within a review team of a document's findings; or consulting experts about technical aspects of methods or findings) should be described. While the description of the processes followed will not allow the reader to draw firm conclusions about judgements made, it will give an indication of the coherence, plausibility and appropriateness of the processes used to inform those judgements.

Item 10: Data extraction

Describe and explain which data or information were extracted from the included documents and justify this selection.

Example

"In order to identify key elements of importance to the success or failure of an intervention in a certain context using a realist perspective, information was gathered on the intervention, the context and the actual "working of the intervention" or the mechanisms. As we intended to discuss the strength of the evidence and the usefulness of the application of realist principles to already published studies, we developed a process of data analysis that was comprehensive and as objective and transparent as possible. Therefore, a data analysis matrix was developed by the team of authors (see Annex 2). During the development of this matrix, the team extensively discussed and defined terms (such as context, mechanisms and outcome) and evaluation levels (such as process, output and outcome)." [31]

Explanation

In a realist synthesis, data extraction assists analysis and synthesis. Reporting on what was extracted and why can add to the transparency of the synthesis process.

The extracted data may consist of descriptions (for example, of the detail of what was done in a program), findings (for example, cure rates, mortality) or explanations about how and why the program may have worked in particular contexts. Of particular interest to the realist reviewer are data that support the use of realist logic to answer the review's question(s) - for example, data on context, mechanisms and outcome configurations, demi-regularities, middle-range and/or program theories. Realist synthesis is used for a wide range of research questions, so it is impossible to be prescriptive about what data should be extracted. However, the link between the research question and the category of data extracted should be clear.

Item 11: Analysis and synthesis processes

Describe the analysis and synthesis processes in detail. This section should include information on the constructs analyzed and describe the analytic process.

Example

"Data synthesis was undertaken either by RP and/or GW and synthesis results were regularly shared and discussed within the review team to ensure validity and consistency in the inferences made. Specifically (where relevant), we attempted to identify prominent recurrent patterns of contexts and outcomes (demi-regularities) in the data and then sought to explain these through the means (mechanisms) by which they occurred. For example, we noted that in our included articles self-reported public support for a ban on smoking in vehicles carrying children was often found to be high amongst smokers. During data synthesis we would then aim to provide an explanation of this demi-regularity through the identification of mechanism(s). As we delved further into our included articles and beyond (through our aforementioned purposive searching) for an explanation, data emerged that smokers harboured within them the wish to want to protect children from harm and also regret at having started smoking. We interpreted these as (realist) mechanisms and, for the former, were able to find substantive (middle-range) theory in the form of the Millean principle [Reference x1] to explain its interaction with context to influence outcomes. When additional studies were sought to enable programme theory testing, data handling processes .... were repeated." [26]

For peer review only - http://bmjopen.bmj.com/site/about/guidelines.xhtml

Wong et al. BMC Medicine 2013, 11:21
http://www.biomedcentral.com/1741-7015/11/21

Explanation

In a realist synthesis, the analysis and synthesis processes occur iteratively and may be sequential or in parallel. At the center of any realist analysis is the application of a realist philosophical 'lens' to data. A realist analysis of data specifically seeks to analyze data using realist concepts. Specifically, realism adheres to a generative explanation for causation - that is, an outcome (O) of interest was generated by relevant mechanism(s) (M) being triggered in context (C). Within or across the included documents, recurrent patterns of outcomes (or demi-regularities) and their associated mechanisms and contexts (CMO configurations) are likely to occur.

During synthesis the goal is to make sense of the analyzed data using theory, at one of two levels. First, theory (or theories) may be sought, developed and/or refined to explain how it is that a program (or part of a program) achieves its outcomes (that is, the mechanism(s) operating within a program) and the contexts in which those mechanisms do and do not fire. This provides a realist program theory. Second, theory (or theories) may be sought, developed and/or refined to explain, at a somewhat more general level, the pattern of contexts, mechanisms and outcomes. A full realist analysis addresses both these levels and attempts to make sense of the relationship between these two levels. Syntheses which address only one level may also be considered realist syntheses assuming that they apply and demonstrate application of a realist philosophy of science. The level(s) of analysis chosen will depend on the review's focus. The theories used may have been developed and/or refined from the data and/or be refinement of existing substantive theory.

The key analytic process in realist review involves iterative testing and refinement of theoretically based explanations using empirical findings in data sources. Reviewers may draw on any appropriate analytic techniques to undertake this testing. Explanation and justification for the choice of techniques should be provided.

Ideally a description should be provided on how all the individuals involved in the review have been involved in the analysis and synthesis processes, and how these evolved as the review took shape.

Results section

The following items should be reported in the results section.

Item 12: Document flow diagram

Provide details on the number of documents assessed for eligibility and included in the review with reasons for exclusion at each stage, as well as an indication of their source of origin (for example, from searching databases, reference lists and so on). You may consider using the example provided (which is likely to need modification to suit the data) in Figure 1.

Example

"See Figure 1: Flow diagram illustrating search process and article disposition." [26]

Explanation

A flow diagram provides an accessible summary of the sequence of steps and gives an indication of the volume of data included and excluded at each step.

Item 13: Document characteristics

Provide information on the characteristics of the documents included in the synthesis.

Example

"Additional File 1 summarises ..., the context, the intervention, the mechanisms triggered and the reported outcomes. Additional File 1 shows that in all the trials, more than one type of intervention was applied to improve CHWs [community health workers] performance. It also shows that the outcomes are reported not in terms of CHW performance, but rather in terms of the consequences of their performance on specific health outcomes." [27]

Explanation

A clear summary of the characteristics of included sources can add to the transparency of the synthesis and some characteristics may help readers judge the coherence and plausibility of inferences. Examples of possibly relevant characteristics of documents that may be worth reporting include, where applicable: full citation, country of origin, study design, summary of key main findings, use made of document in the synthesis and relationship of documents to each other (for example, there may be more than one document reporting on an intervention). While considering specific requirements of any particular publication, reviewers may wish to tabulate key characteristics.

Item 14: Main findings

Present the key findings with a specific focus on theory building and testing.

Example

"Using this theoretical concept, we hypothesized that equitable partnerships, with the stakeholders' participation throughout the project, succeed largely through synergy. Through the synthesis process using CMO configuring, we refined the theory by demonstrating that synergy is both an outcome and a context for partnership development - so that when synergy generated positive outcomes (e.g., enhanced trust or improved data collection), those outcomes generated new synergy. Expanding this logic, we demonstrated how partnership synergy created momentum over time, producing resilience in the face of obstacles as well as sustaining health-related goals, extending

For peer review only

programs and infrastructure, and creating new and unexpected ideas and activities." [30]

Explanation

The defining feature of a realist synthesis is the nature of the theory(ies) it offers. Such a theory explains why a social program/intervention generates particular outcomes in particular contexts, in terms of one or more mechanisms - that is how the program's infrastructure and resources trigger particular decisions or behaviors in human participants. Program theories are usually 'middle-range' - that is, specific enough to generate propositions that can be tested about aspects of the program but sufficiently abstract to be applicable to other programs. Mechanisms are contingent: they are causal processes that have a tendency to occur in a particular set of conditions, but which do not always occur (because the circumstances have to be right for any particular mechanism to operate, and because many mechanisms can operate concurrently, sometimes cancelling each other out).

The validity of a review which is described as 'realist' and which talks about program theories or mechanisms but which expresses these as simple and linear relationships between variables should be questioned.

The findings of a realist synthesis consist largely of inferences about the links between context, mechanism and outcome and the theory(ies) that seek to account for these links. It is important that where inferences are made these are clearly articulated. Where possible, especially for key findings, it is important to include an explanation to show how these inferences were arrived at.

Transparency of the synthesis process can be demonstrated, for example, by including such things as a detailed worked example, verbatim quotes from primary sources, and (if appropriate) an exploration of disconfirming data (that is, findings which appeared to refute the program theory but which, on closer analysis, could be explained by other contextual influences).

When presenting inferences about context-mechanism-outcome configurations, reviewers should be clear about what they have categorized as context, what as mechanism and what as outcome. In a realist synthesis a mechanism involves the interaction between particular inputs (or resources) and human reasoning, which produces a particular outcome (or not).

More than one piece of data might be needed to support an inference. It is sometimes appropriate to build the argument for an inference as an unfolding narrative in which successive data sources increase the strength of the inference [32]. Provide enough details about each data item to identify its source and enable readers to make judgements about its relevance and rigor.

Discussion section

The following Items should be reported in the discussion section.

Item 15: Summary of findings

Summarize the main findings, taking into account the synthesis' objective(s), research question(s), focus and intended audience(s).

Example

"This realist review of 249 primary studies has produced two key findings which are important, if somewhat unsurprising. First, Internet-based courses must engage their target group of learners to use the technology. This is likely to occur only if the technology is perceived as 'useful' (e.g.increases access to learning or saves time) and 'easy to use', though benefits in the former can outweigh challenges in the latter. Second, 'interactivity' is highly valued by learners. Learners wanted to be able to enter into a dialogue with the course tutor, fellow students and/or a virtual tutorial and obtain ongoing feedback on their understanding and performance." [29]

Explanation

In order to place the findings in the context of the wider literature and any specific policy need, it is necessary to summarize briefly what has been found. This section should be succinct and balanced, explaining the relevance of one or more key theories that emerged from the analysis and highlighting the strength of evidence for the main inferences. This should be done with careful attention to the needs of the main users of the synthesis.

Item 16: Strengths, limitations and future research directions

Discuss both the strengths of the review and its limitations. These should include (but need not be restricted to) (a) consideration of all the steps in the synthesis process and (b) comment on the overall strength of evidence supporting the explanatory insights that emerged.

The limitations identified may point to areas where further work is needed.

Example

"We explicitly chose to do a realist review of the RCTs [randomized controlled trials] to see what they could additionally yield. While the CHWs [community health workers] were an important component of the interventions being tested in the RCTs, none of the RCTs under review explicitly focused on performance of the CHW as an outcome. The RCTs under review offered a fair amount of information about the interventions, only some information about context - allowing us to formulate only generic hypotheses. ...

... Authors seldom described or discussed the mechanisms that explained their study outcomes. We realise that

For peer review only

the RCT design, the exacting reporting requirements and word limits of journals, restrict authors from sharing all their operational experiences. In addition RCTs tend to report average effects and not differential effects of interventions, and less so of the context and rarely of the mechanisms triggered by their interactions. This makes the RCTs less useful for answering the questions regarding how interventions work. These generic hypotheses seem to be recurring in the literature, however they have not been explicitly tested across contexts." [27]

Explanation

Realist synthesis may be constrained by time and resources, by the skill mix and collective experience of the research team, by the scope of the review's questions or objectives and/or by anticipated or unanticipated challenges in the data. These should be made explicit so that readers can interpret the findings in the light of them. A common challenge in realist synthesis is that in order to focus the synthesis, some material is omitted at each successive stage. Some aspects of the topic area, therefore, end up being reviewed in detail and rich explanatory insights produced for these. Other aspects are neglected (relatively or absolutely). It is thus inevitable that in generating illumination, the synthesis will also cast shadows. These should be highlighted in the discussion so as to indicate areas where other syntheses might focus.

Strengths and/or limitations associated with any modifications made to the synthesis process should also be reported and justified.

Item 17: Comparison with existing literature

Where applicable, compare and contrast the synthesis' findings with the existing literature (for example, other reviews) on the same topic.

Example

"We were unable to find any comparable attempt at providing an evidence-based-policy framework such as ours. However, we acknowledge that some sections of our framework may be found in sources we have not uncovered and also as tacit knowledge within the heads of seasoned practitioners (e.g. advocates or legislators). We do however hope that our attempts to develop and test it on our one 'case study' will make a primordial tool that will be useful to policy and decisions makers less well versed in the arena of public health legislation." [26]

Explanation

Comparing and contrasting the findings from a synthesis with the existing literature may help readers to put these into context. For example, this item might cover questions such as: How does this synthesis compare to other reviews (for example, were they theory-driven?); What does this synthesis add?; Which body of work in particular does it add to?; Has this synthesis reached the same or different conclusion to previous reviews?; and Has it answered a question previously identified as important in the field?

Item 18: Conclusion and recommendations

List the main implications of the findings and place these in the context of other relevant literature. If appropriate, offer recommendations for policy and practice.

Example

"Our realist review was based on a housing intervention in the United States, but the results can potentially be applied to urban centers in other nations that implement housing interventions that involve moving families. When a family moves, the experience is likely to be different for each member of the household, and differences in mental health outcomes of moving may occur (Reference x1). All communities, rich or poor, and irrespective of geographic location, should be viewed as complex systems, and as composed of people with social relationships that influence the functioning and health of community members." [28]

Explanation

A clear line of reasoning is needed to link the findings (Results section) with the implications (Discussion and/or Conclusion). If the synthesis is small and preliminary, or if the coherence and plausibility of evidence behind the inferences is weak or moderate, statements about implications for practice and policy should be appropriately guarded.

If recommendations are given, these should take into account the focus of the synthesis and needs of the intended audience and be presented appropriately. The explanations in realist analysis are highly dependent on contextual influences. It follows that recommendations must be contingent (for example, only under certain contexts will a particular mechanism be triggered to generate the desired outcome) rather than statements that X should or should not be done.

Item 19: Funding

Provide details of funding source (if any) for the synthesis, the role played by the funder (if any) and any conflicts of interests of the reviewers.

Example

"We gratefully acknowledge a financial contribution from the Dutch Development Cooperation (DGIS)." [25]

Explanation

The source of funding for a synthesis and/or personal conflicts of interests may influence the research question, methods, data analysis and conclusions. No review is a 'view from nowhere', and readers will be better able to interpret the review if they know why it was done and for which sponsor.

For peer review only

Wong et al. BMC Medicine 2013, 11:21
http://www.biomedcentral.com/1741-7015/11/21

If a synthesis is published, the process for reporting funding and conflicts of interest as set out by the publication concerned should be followed.

Discussion

We have developed these publication standards for realist synthesis (which we view as synonymous with realist review) by drawing together a range of sources - namely, existing published evidence, a Delphi panel and comment, discussion and feedback from a mailing list, training sessions and workshops. We hope these standards will lead to greater consistency and rigor of reporting and, thereby, make the outputs of realist synthesis more accessible, usable and helpful to different stakeholders.

This publication standard is not a detailed guide of how to undertake a realist synthesis. Other resources, both published (see Background) and in preparation, are better suited for this purpose. These standards have been developed as a guide to assist the quality of reporting of realist syntheses and the work of publishers, editors and reviewers. As part of the RAMESES project, we will be developing and disseminating both training materials and quality standards for realist synthesis [20].

Because realist synthesis is used for a broad range of topics and questions, and because it involves making judgements and inferences rather then checking against or following a technical checklist, it is impossible to be prescriptive about what exactly must be done in a review. The guiding principle is that transparency is important, as this will help readers to decide for themselves if the arguments for the judgements made were reasonable, both for the chosen topic and from a methodological perspective. We strongly encourage review authors to provide detail on what they have done and how - in particular with respect to the analytic processes used. These standards are intended to supplement rather than replace the exercise of judgement by editors, reviewers, readers and users of realist syntheses. We have tried to indicate in each item where judgement needs to be exercised.

The explanatory and theory-driven focus of realist syntheses means that detailed data may need to be reported in order to provide enough support for inferences and/or judgments made. While developing these publication standards, it became apparent that in some cases the word count limitations imposed by journals did not enable review teams to fully explain aspects of their synthesis - such as how judgments were made or inferences arrived at. Alternative ways of providing the necessary detail may need to be found, such as online appendices or additional files available from authors on request.

Previous efforts to develop publication standards have sometimes been criticized for being too 'ivory-tower' and failing to take account of real-world problems faced by reviewers. In an effort to redress this problem in the RAMESES project, we sought from the outset to engage not just senior academics but also junior and mid-career researchers, practitioners, policymakers and publishers in the development of the standards and to capture real-life challenges of ongoing realist syntheses as these emerged.

Conclusions

We have developed these publication standards for realist syntheses by drawing on a range of sources. Our hope is that these standards will lead to greater consistency and rigor of reporting and make the outputs of realist syntheses more accessible, usable and helpful to different stakeholders. Realist synthesis is a relatively new approach to evidence synthesis and with increasing use and methodological development, changes are likely to be needed to any publication standards. We hope to continue capturing and improving these publication standards, through our email list [21] and wider links and discussions with researchers and those who commission, sponsor, publish and use realist syntheses.

Abbreviations

ESRC: Economic and Social Research Council; RAMESES: Realist And MEta-narrative Evidence Syntheses: Evolving Standards).

Acknowledgements

This project was funded by the National Institute for Health Research Health Services and Delivery Research Programme (NIHR HS&DR) - project number 10/1008/07.

We thank the following individuals for their participation in the RAMESES Group and contributions to the Delphi panel:

Dave Baker, Sinai Hospital of Baltimore (Baltimore, USA); Marcello Bertotti, University of East London (London, UK); Allan Best, InSource (Vancouver, Canada); Margaret Cargo, University of South Australia (Adelaide, Australia); Simon Carroll, University of Victoria (Victoria, Canada); Colleen Davison, Queens University, (Kingston, Canada); Marjolein Dieleman, Royal Tropical Institute (Amsterdam, Netherlands); Tim Dornan, Maastricht University (Maastricht, Netherlands); Ruth Garside, Peninsula College of Medicine and Dentistry (Exeter, UK); Bradford Gray, Milbank Quarterly (New York, USA); Joanne Greenhalgh, University of Leeds (Leeds, UK); Lois Jackson, Dalhousie University (Halifax, Canada); Justin Jagosh, McGill University (Montreal, Canada); Monika Kastner, University of Toronto (Toronto, Canada); James Lamerton, Sunshine Coast Division of General Practice (Cotton Tree, Australia); Fraser MacFarlane, Queen Mary, University of London (London, UK); Bruno Marchal, Institute of Tropical Medicine (Antwerp, Belgium); Tracey McConnell, Queen's University (Belfast, UK); Gemma Moss, Institute of Education (London, UK); Douglas Noble, Queen Mary, University of London (London, UK); Patricia O'Campo, University of Toronto (Toronto, Canada); Mark Pearson, Peninsula College of Medicine and Dentistry (Exeter, UK); Pierre Pluye McGill University (Montreal, Canada); Henry Potts, University College London (London, UK); Barbara Riley, University of Waterloo, (Waterloo, Canada); Glenn Robert, Kings College London (London, UK); Jessie Saul, North American Research & Analysis, Inc (Faribault, USA); Paul Shekelle, RAND Corporation (Santa Monica, USA); Neale Smith, University of British Columbia (Vancouver, Canada); Sanjeev Sridharan, University of Toronto (Toronto, Canada); Deborah Swinglehurst, Queen Mary, University of London (London, UK); Nick Tilley, University College London (London, UK); Kieran Walshe, University of Manchester (Manchester, UK).

All the authors (except JB) were also members of the Delphi panel.

Author details

[1]Centre for Primary Care and Public Health, Queen Mary University of London, 58 Turner Street, London E1 2AB, UK. [2]Community Matters, P.O. Box 443, Mount Torrens, SA 5244, Australia. [3]John W. Scott Health Sciences Library, University of

For peer review only - http://bmjopen.bmj.com/site/about/guidelines.xhtml

Alberta, Edmonton, AB T6G 2R7, Canada. [4]Department of Social Research Methodology, University of Leeds, Leeds LS2 9JT, UK.

Authors' contributions

GWo carried out the literature review. JB searched the literature for realist syntheses. GWo, TG, GWe and RP analyzed the findings from the review and produced the materials for the Delphi panel. They also analyzed the results of the Delphi panel. GWo, TG, GWe and RP conceived of the study and participated in its design. GWo coordinated the study and ran the Delphi panel. All authors read and approved the final manuscript.

Competing interests

The authors declare that they have no competing interests. The views and opinions expressed therein are those of the authors and do not necessarily reflect those of the HS&DR program, NIHR, NHS or the Department of Health.

Received: 27 September 2013 Accepted: 29 January 2013
Published: 29 January 2013

References

1. Pawson R: *Evidence-based Policy: A Realist Perspective* London: Sage; 2006.
2. Pearson M, Coomber R: **The challenge of external validity in policy-relevant systematic reviews: a case study from the field of substance misuse.** *Addiction* 2010, **105**:136-145.
3. Dixon-Woods M, Agarwal S, Jones D, Young B, Sutton A: **Synthesising qualitative and quantitative evidence: a review of possible methods.** *J Health Serv Res Policy* 2005, **10**:45-53.
4. Barnett-Page E, Thomas J: **Methods for the synthesis of qualitative research: a critical review.** *BMC Med Res Methodol* 2009, **9**:59.
5. Pope P, Mays N, Popay J: *Synthesizing Qualitative and Quantitative Health Research: A Guide to Methods* Maidenhead, UK: Open University Press; 2007.
6. Gough D, Oliver S, Thomas J: *An Introduction to Systematic Reviews* London: Sage; 2012.
7. Kastner M, Tricco A, Soobiah C, Lillie E, Perrier L, Horsley T, Welch V, Cogo E, Antony J, Straus S: **What is the most appropriate knowledge synthesis method to conduct a review? Protocol for a scoping review.** *BMC Med Res Methodol* 2012, **12**:114.
8. Merton R: *On Theoretical Sociology. Five Essays, Old and New* New York: The Free Press; 1967.
9. Astbury B, Leeuw FL: **Unpacking black boxes: mechanisms and theory building in evaluation.** *Am J Eval* 2010, **31**:363-381.
10. Shepperd S, Lewin S, Straus S, Clarke M, Eccles M, Fitzpatrick R, Wong G, Sheikh A: **Can we systematically review studies that evaluate complex interventions?** *PLoS Med* 2009, **6**:e1000086.
11. Rycroft-Malone J, McCormack B, Hutchinson AM, DeCorby K, Bucknall TK, Kent B, Schultz A, Snelgrove-Clarke E, Stetler CB, Titler M, Wallin L, Wilson V: **Realist synthesis: illustrating the method for implementation research.** *Implement Sci* 2012, **7**:33.
12. Robert E, Ridde V, Marchal B, Fournier P: **Protocol: a realist review of user fee exemption policies for health services in Africa.** *BMJ Open* 2012, **2**: e000706.
13. Pawson R: **Evidence-based policy: the promise of 'realist synthesis'.** *Evaluation* 2002, **8**:340-358.
14. Pawson R, Greenhalgh T, Harvey G, Walshe K: *Realist Synthesis - an Introduction. ESRC Working Paper Series* London: Economic and Social Research Council; 2004.
15. Pawson R, Greenhalgh T, Harvey G, Walshe K: **Realist review–a new method of systematic review designed for complex policy interventions.** *J Health Serv Res Policy* 2005, **10(Suppl 1)**:21-34.
16. Moher D, Hopewell S, Schulz K, Montori V, Gotzsche P, Devereaux P, Elbourne D, Egger M, Altman D, for the CONSORT Group: **CONSORT 2010 explanation and elaboration: updated guidelines for reporting parallel group randomised trial.** *BMJ* 2010, **340**:c869.
17. Brouwers M, Kho M, Browman G, Burgers J, Cluzeau F, Feder G, Fervers B, Graham I, Grimshaw J, Hanna S, Littlejohns P, Makarski J, Zitzelsberger L, AGREE Next Steps Consortium: **AGREE II: advancing guideline development, reporting and evaluation in healthcare.** *Can Med Assoc J* 2010, **182**:E839-842.
18. Liberati A, Altman DG, Tetzlaff J, Mulrow C, Gøtzsche PC, Ioannidis JP, Clarke M, Devereaux PJ, Kleijnen J, Moher D: **The PRISMA statement for reporting systematic reviews and meta-analyses of studies that evaluate healthcare interventions: explanation and elaboration.** *BMJ* 2009, **339**: b2700.
19. Davidoff F, Batalden P, Stevens D, Ogrinc G, Mooney S, SQUIRE Development Group: **Publication guidelines for improvement studies in health care: evolution of the SQUIRE project.** *Ann Intern Med* 2008, **149**:670-676.
20. Greenhalgh T, Wong G, Westhorp G, Pawson R: **Protocol - realist and meta-narrative evidence synthesis: evolving standards (RAMESES).** *BMC Med Res Methodol* 2011, **11**:115.
21. RAMESES JISCM@il: [www.jiscmail.ac.uk/RAMESES].
22. Wong G, Greenhalgh T, Westhorp G, Pawson R: **Realist methods in medical education research: what are they and what can they contribute?** *Med Educ* 2012, **46**:89-96.
23. Wong G: **The internet in medical education: a worked example of a realist review.** In *Synthesising Qualitative Research: Choosing the Right Approach.* Edited by: Hannes K, Lockwood C. Chicester, UK: Wiley-Blackwell; 2011:.
24. EQUATOR Network: [http://www.equator-network.org/home/].
25. Dieleman M, Gerretsen B, van der Wilt GJ: **Human resource management interventions to improve health workers' performance in low and middle income countries: a realist review.** *Health Res Policy Syst* 2009, **7**:7.
26. Wong G, Pawson R, Owen L: **Policy guidance on threats to legislative interventions in public health: a realist synthesis.** *BMC Public Health* 2011, **11**:222.
27. Kane SS, Gerretsen B, Scherpbier R, Dal Poz M, Dieleman M: **A realist synthesis of randomised control trials involving use of community health workers for delivering child health interventions in low and middle income countries.** *BMC Health Serv Res* 2010, **10**:286.
28. Jackson L, Langille L, Lyons R, Hughes J, Martin D, Winstanley V: **Does moving from a high-poverty to lower-poverty neighborhood improve mental health? A realist review of 'Moving to Opportunity'.** *Health Place* 2009, **15**:961-970.
29. Wong G, Greenhalgh T, Pawson R: **Internet-based medical education: a realist review of what works, for whom and in what circumstances.** *BMC Med Educ* 2010, **10**:12.
30. Jagosh J, Macaulay AC, Pluye P, Salsberg J, Bush PL, Henderson J, Sirett E, Wong G, Cargo M, Herbert CP, Seifer SD, Green LW, Greenhalgh T: **Uncovering the benefits of participatory research: implications of a realist review for health research and practice.** *Milbank Q* 2012, **90**:311-346.
31. Dieleman M, Kane S, Zwanikken P, Gerretsen B: *Realist Review and Synthesis of Retention Studies for Health Workers in Rural and Remote Areas* Geneva, Switzerland: World Health Organization; 2011.
32. Pawson R, Wong G, Owen L: **Known knowns, known unknowns, unknown unknowns: the predicament of evidence-based policy.** *Am J Eval* 2011.

doi:10.1186/1741-7015-11-21
Cite this article as: Wong *et al.*: RAMESES publication standards: realist syntheses. *BMC Medicine* 2013 **11**:21.

Submit your next manuscript to BioMed Central and take full advantage of:

- **Convenient online submission**
- **Thorough peer review**
- **No space constraints or color figure charges**
- **Immediate publication on acceptance**
- **Inclusion in PubMed, CAS, Scopus and Google Scholar**
- **Research which is freely available for redistribution**

Submit your manuscript at
www.biomedcentral.com/submit

BioMed Central

For peer review only
