# Supplementary material for: Integrating palliative care and heart failure: a protocol for a realist synthesis (PalliatHeartSynthesis)
Source: BMJ Open. 2022 Jan 7;12(1):e058848. doi: 10.1136/bmjopen-2021-058848 (PMC8744113; doi:10.1136/bmjopen-2021-058848)
Supplement: Reviewer comments [file bmjopen-2021-058848.reviewer_comments.pdf]

PEER REVIEW HISTORY

BMJ Open publishes all reviews undertaken for accepted manuscripts. Reviewers are asked to complete a checklist review form (**http://bmjopen.bmj.com/site/about/resources/checklist.pdf**) and are provided with free text boxes to elaborate on their assessment. These free text comments are reproduced below.

ARTICLE DETAILS

**TITLE (PROVISIONAL)**	Integrating Palliative Care and Heart Failure: a protocol for a realist synthesis (PalliatHeartSynthesis)
**AUTHORS**	McConnell, Tracey; Burden, John; Duddy, Claire; Hill, Loreena; Howie, Clare; Jones, Bob; Ruane, Bob; Wong, Geoff; Reid, Joanne

This article was not externally reviewed at BMJ Open. Protocols that have been independently assessed prior to submission to BMJ Open are usually fast-tracked to publication on the grounds that further substantial changes will not be possible. This independent assessment will usually be external, independent review for both a substantial grant award from a non-commercial or government funder and ethics approval.
